# Supplementary material for: A statistical framework for high-content phenotypic profiling using cellular feature distributions
Source: Commun Biol. 2022 Dec 22;5:1409. doi: 10.1038/s42003-022-04343-3 (PMC9780213; doi:10.1038/s42003-022-04343-3)
Supplement: Supplementary file 2 — Supplementary Table and Figures [file 42003_2022_4343_MOESM2_ESM.pdf]

Supplementary information: Supplementary Table 1 and

Supplementary Figures 1 - 9

A statistical framework for high-content phenotypic profiling using cellular feature distributions

Yanthe E. Pearson[1], Stephan Kremb[1], Glenn L. Butterfoss[1], Xin Xie[1], Hala Fahs[1], Kristin C. Gunsalus[1,2*]

[1]Center for Genomics and Systems Biology, New York University Abu Dhabi, P. O. Box 129188, Abu Dhabi, United Arab Emirates

[2]Department of Biology and Center for Genomics and Systems Biology, New York University, New York, NY 10003, USA

* Corresponding author: Kristin C. Gunsalus
Email: kcg1@nyu.edu

Supplementary Table 1: R scripts and data files underlying each manuscript figure.

Supplementary Figure 1: Adjustment of positional effects and data standardization across different plates.

Supplementary Figure 2: Statistical distance measurement among replicates.

Supplementary Figure 3: Full feature cytological profile.

Supplementary Figure 4: Full feature EMD fingerprints of individual control samples.

Supplementary Figure 5: Summary of phenotypes for 65 compounds.

Supplementary Figure 6: Divergent phenotypes.

Supplementary Figure 7: Feature reduction.

Supplementary Figure 8: Comparison of cytological profiles.

Supplementary Figure 9: Radial plot fingerprints of 65 compounds.

Supplementary Table 1: R scripts and data files underlying each manuscript figure.

Manuscript Figure	Description	R script	Data files
Figure 2a	Heatmap of compound similarity based on Tanimoto distance	Figure2A_tanimoto.R	Tanimoto_figure2.csv
Figure 2c	Cell counts shown as heatmap form	figure2C_count_heatmap.R	raw_medians_fig2C.csv
Figure 2d	Cell counts shown as a scatterplot	figure2D_count_scatter.R	raw_medians_fig2C.csv
Figure 2e	Per well density curve of controls	Figure2E_dmso_curves.R	cell_data_DMSO_figure2g.csv
Figure 2f	Cell cycle dose response plot of mitoxantrone treated cells	Figure2F_mitoxantrone.R	cell_data_mitoxantrone_figure2f.csv
Figure 2g	Density plots showing quartiles for a group of compounds	figure2G_quartiles.R	Data_fig2G.csv
Figure 3a	Heatmap of control well medians	figure3A.R	nucfeatures_medians_fig3A.csv
Figure 3b	Summary of two-way ANOVA test	figure3B_anova.R	anova_output_fig3b.csv
Figure 3c	Heatmaps showing positional adjustment	figure3C_positioneffect.R	Well_medians_fig3c.csv
Figure 3d	Cell cycle distributions	figure3D.R	cell_data_fig3D.csv
Figure 4a	Plots for replicate feature distributions, both treatment and control	figure4A.R	cell_data_figure4A_control.csv<br>cell_data_figure4A_treatment1.csv<br>cell_data_figure4A_treatment2.csv
Figure 4b	Plots of density curves and CDF curves for two data samples	figure4B.R	No data files required
Figure 4c	Plot for distribution of statistical scores	figure4C-D.R	control_data_fig4C.csv<br>treatment_data_fig4C.csv
Figure 4d	Plot of sorted features	figure4C-D.R	treatment_replicate_test_allpanels_fig4D.csv<br>control_replicate_test_allpanels_fig4D.csv
Figure 5a	Plot of replicate distributions<br>R script Includes EMD calculation	figure5A.R	cell_data_figure5a_vincristine_control.csv
Figure 5b,c	Global control and individual treatment groups	figure5B-C.R	treated_cells_data_fig5B-C.csv<br>control_cells_data_fig5B-C.csv
Figure 5d,e Supplementary Figure 4	Full feature EMD fingerprints of individual control samples.	figure5D-E_fullfeature.R<br>radarchart2_new.R	raw_emd_profile174.csv
Figure 5f	Plot of Vincristine fingerprint	Figure5F_vincristine.R<br>radarchart2_new.R	ScaledEMDprofile_69feats_785treatments.csv
Figure 6a	Heatmap of similarity among treatments	Figure6A_heatmap.R	fullfeature_EMDprofile_785treatments.csv
Figure 6b	Run the umap and plot phenotypic trajectories while projecting cell count onto UMAP.	line_segments.R<br>Figure6B_umap.R	fullfeature_EMDprofile_785treatments.csv
Figure 7a	UMAP paths	line_segments.R<br>figure7A_umap_paths.R	UMAP_dimensions_69feats_785treatments_new .csv
Figure 7b	Cell count dose response	figure7B.R	cell_counts.csv
Figure 7c	Cell cycle distributions	Figure7C_cellcycle_four.R	figure7c_treatments.csv<br>figure7c_control.csv
Figure 7d	Radial plots (see Figure 5f)		

Supplementary Table 1: A summary of all R scripts and data files used for each figure in the manuscript. The files can be found on the GitHub within individual figure folders at https://github.com/GunsalusPiano/EMD.

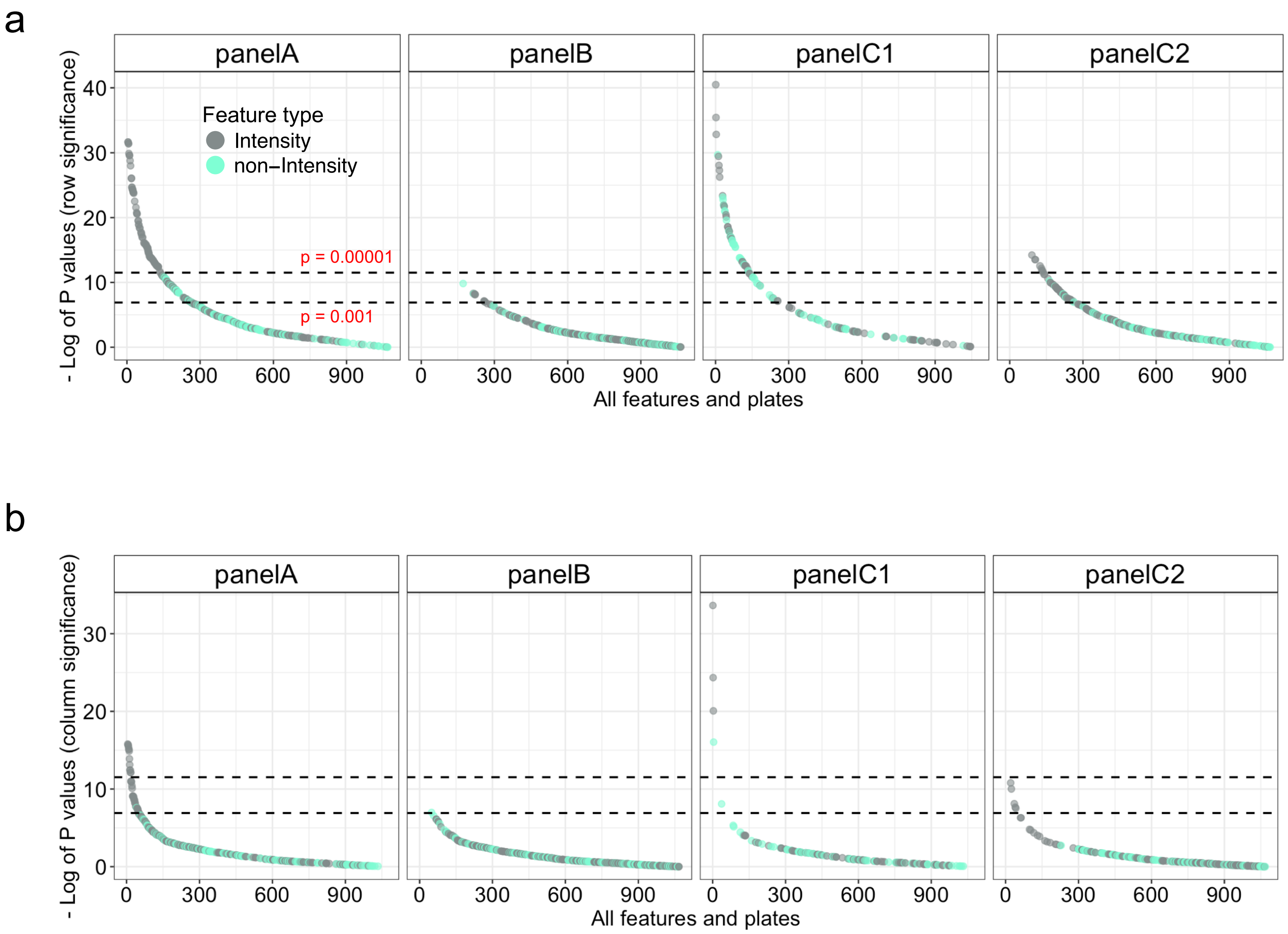

Supplementary Figure 1: Adjustment of positional effects and data standardization across different plates.

c

DMSO- control distributions

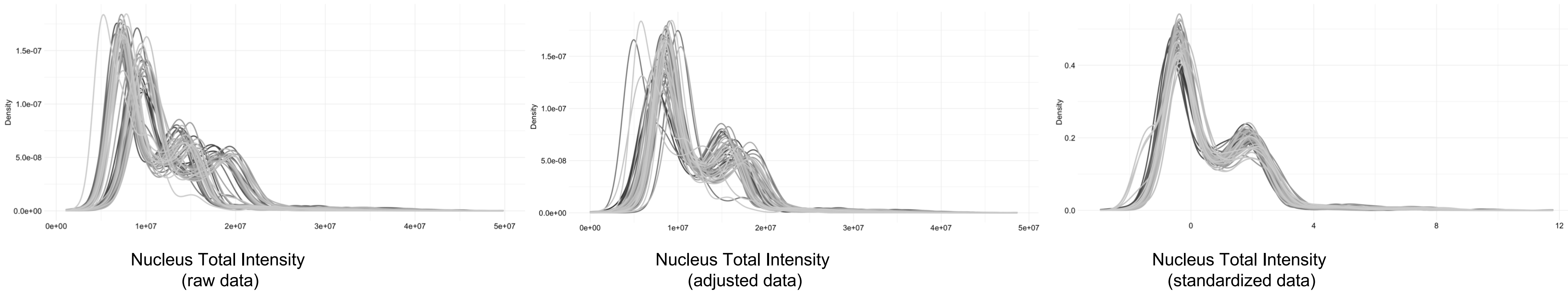

Supplementary Figure 1: Adjustment of positional effects and data standardization across different plates.

d

Plate 1 replicate 1: Cell cycle feature under different chemical perturbations

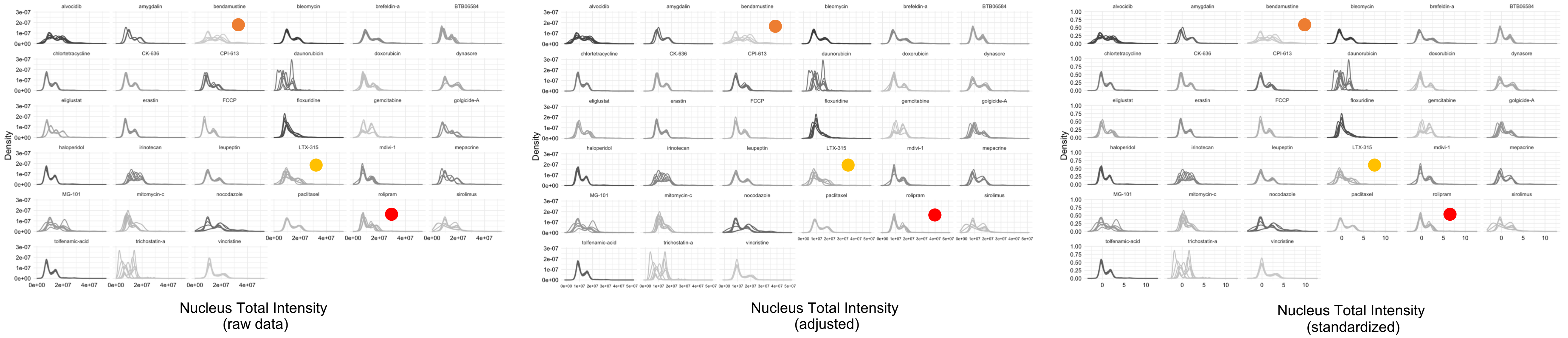

e

Plate 1 replicate 3: Cell cycle feature under different chemical perturbations

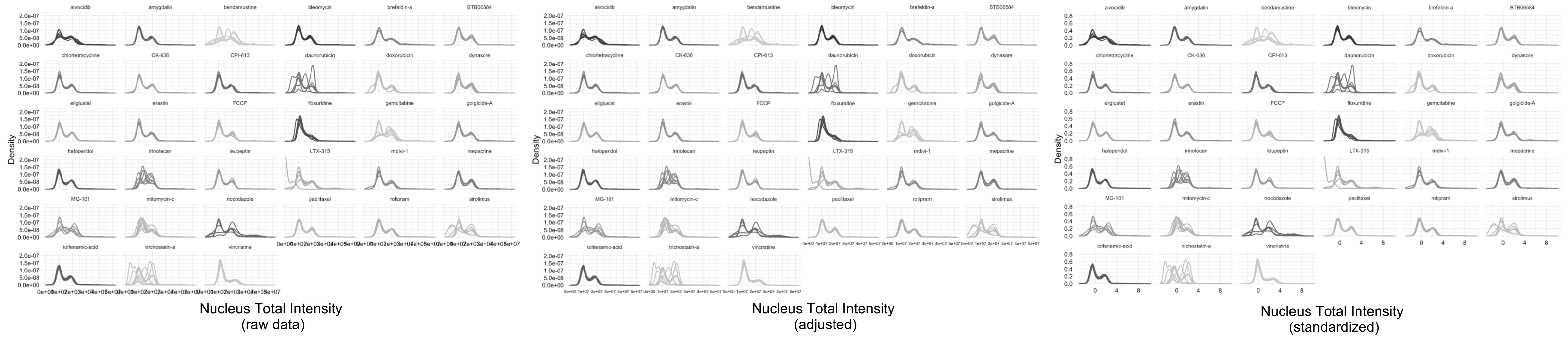

Supplementary Figure 1: Adjustment of positional effects and data standardization across different plates.

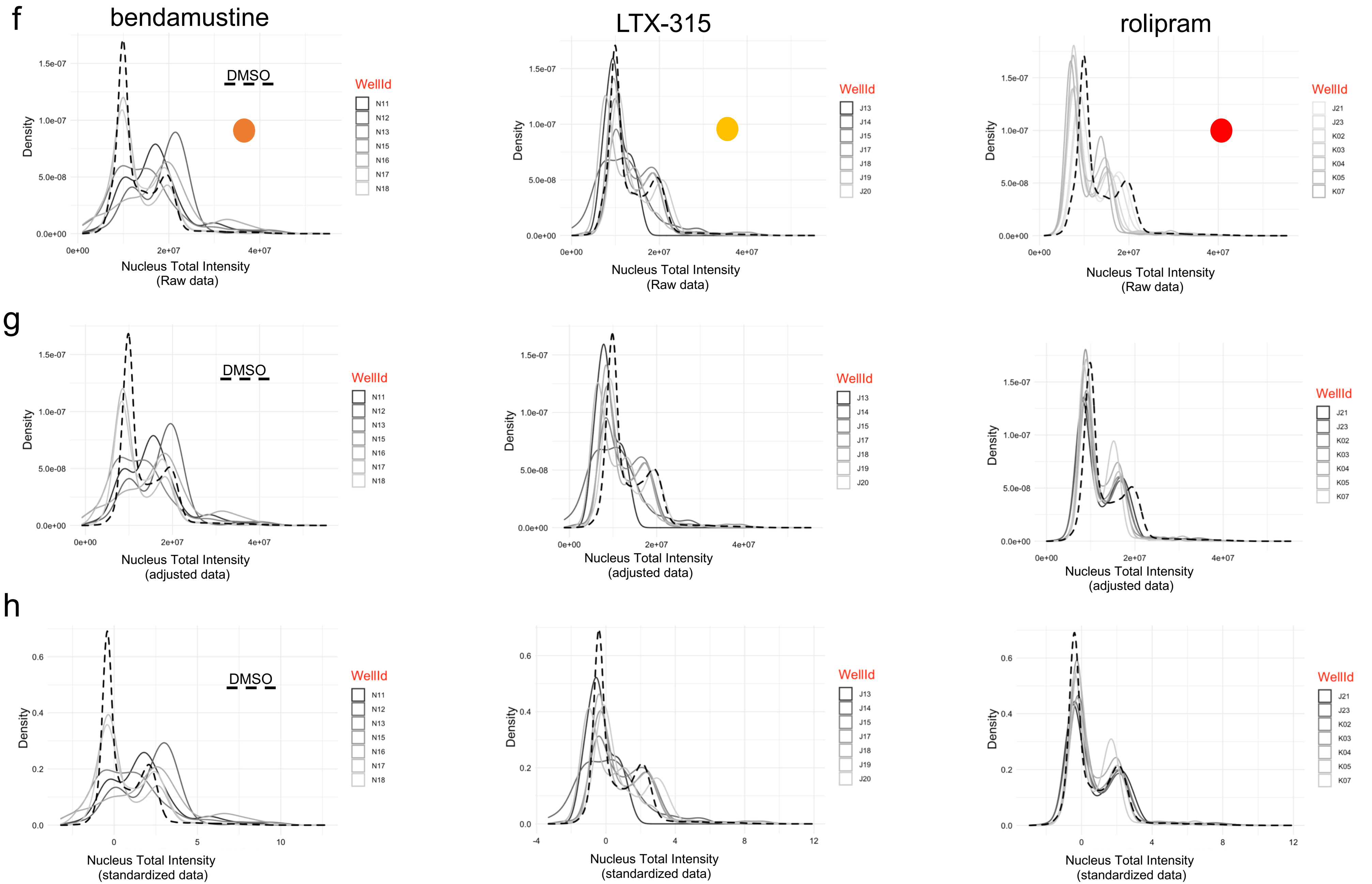

Supplementary Figure 1: Adjustment of positional effects and data standardization across different plates.

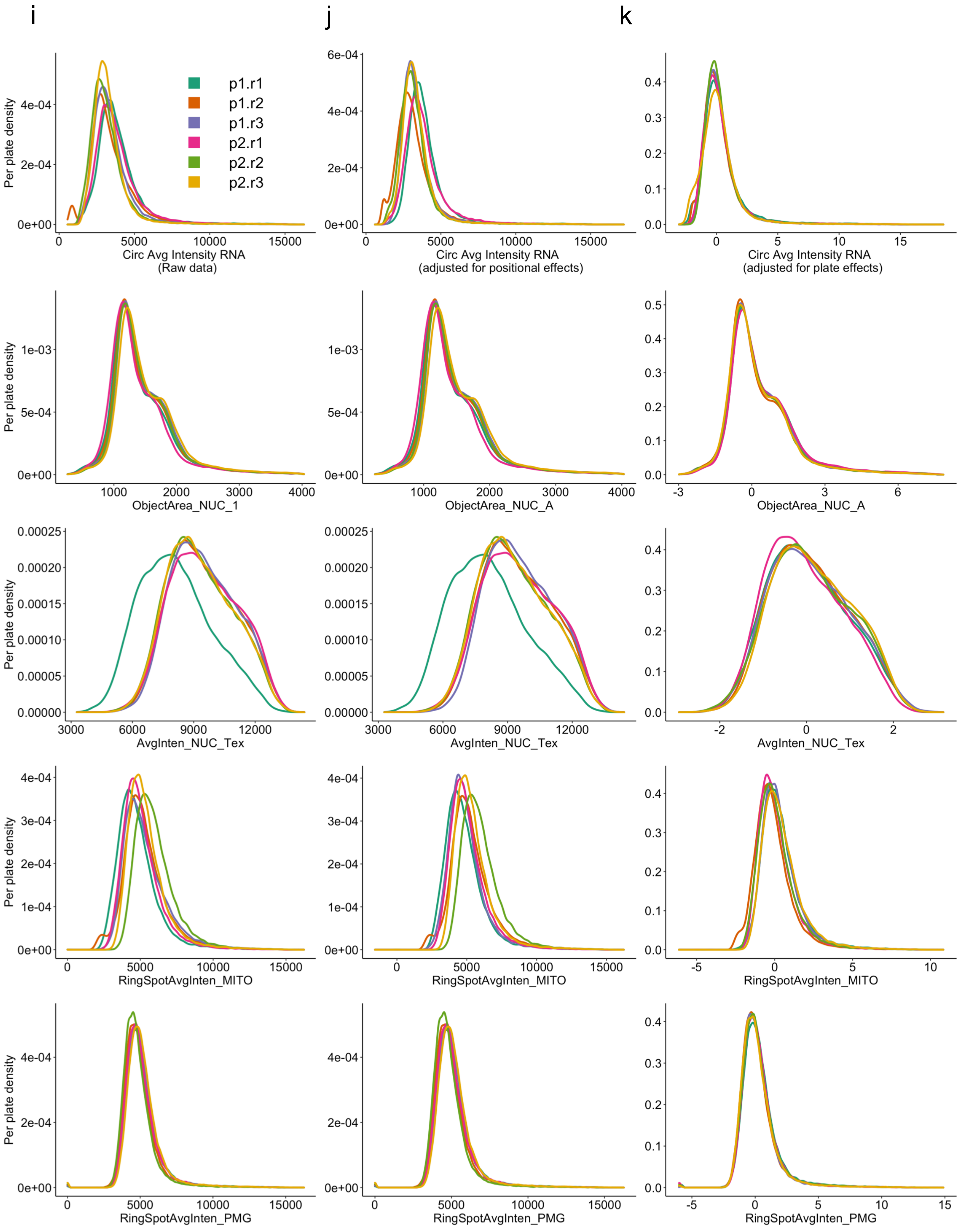

Supplementary Figure 1: Adjustment of positional effects and data standardization across different plates.

Supplementary Figure 1: Adjustment of positional effects and data standardization across different plates.

(**a** - **b**) Positional effect detection by two-way ANOVA is applied to control well medians, for all measured features and all plates. Plots show row (**a**) and column (**b**) dependencies for all control wells, ordered by decreasing significance (p-value increasing) for each assay panel. Features related to Intensity (gray) tend to be more sensitive to positional effects than non-Intensity (cyan) features related to area, texture, and shape.

(**c**) Per well distributions of raw, adjusted, and standardized DMSO-control cell populations from plate1 1 rep 1 (see Fig. 3d).

(**d** - **e**) Two replicate plates of cell cycle feature in response to 231 unique treatment conditions including 33 compounds at seven different concentrations. Three compounds bendamustine, LTX-315, and rolipram color labeled as orange (column 1), yellow (column 2) and red (column 3) demonstrate the data correction effects on cell feature distributions (see **f** - **h**).

(**i** - **k**) Correction for row and column positional effects and standardization of plate-to-plate variation for representative features (by row): RNA, Nucleus Area, Nucleus Texture, Mitochondria, Plasma membranes and Golgi (PMG).

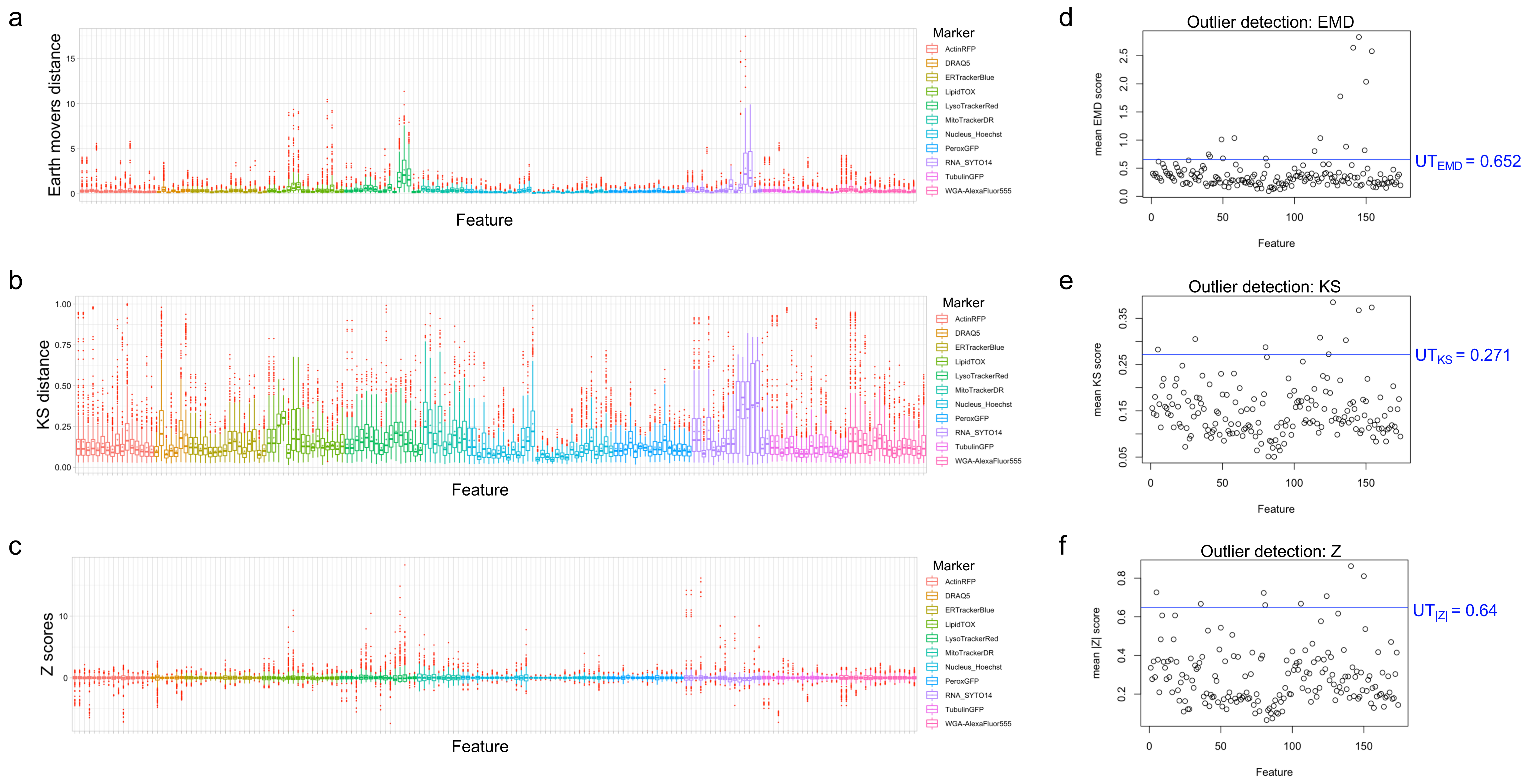

Supplementary Figure 2: Statistical distance measurement among replicates.

Supplementary Figure 2. Statistical distance measurement among replicates.

(**a** – **c**) Per-feature distributions for each of the three statistical metrics, color coded by cytological marker. (**a**) EMD scores are strictly positive and highly sensitive to feature distribution differences; (**b**) KS scores are bounded between 0 and 1; (**c**) robust Z-scores account for both positive and negative differences. EMD and robust Z-scores identify RNA (SYTO14), Lysosome (LysoTracker Green) channels (SYTO 14 and LysoTracker Green), and Lipid (LipidTOX) features with extreme outliers, whereas KS has difficulty discriminating noisy features.

(**d** – **f**) Outlier detection: Using per-feature averages and interquartile range (IQR) outlier detection (Upper threshold value (UT) = 1.5 × IQR + upper quartile, blue line) to identify features which fall too far from the expected range of values we find that EMD score (**d**) detects and separates outliers from the group more efficiently than KS (**e**) and robust Z-scores (**f**).

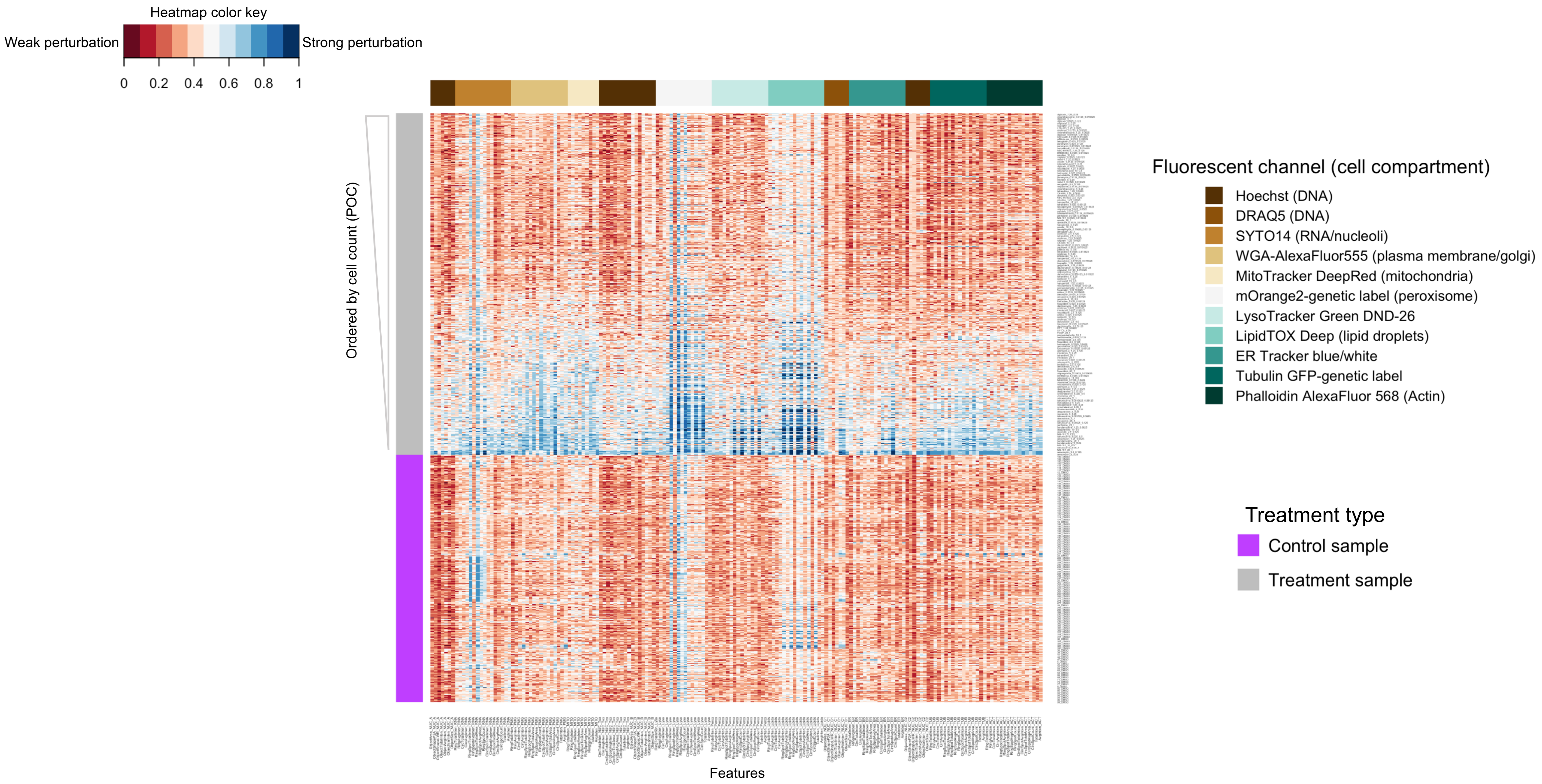

Supplementary Figure 3: Full feature cytological profile.
Heatmap summary of EMD profiles for all 455 treatments (grey rows); including 65 compounds at 7 concentrations each, and 330 DMSO-control samples (purple rows). The full profile is log transformed and features are min-max scaled to the range [0, 1]. Treatment profiles (grey rows) are further sorted by their cell count as percent of control (POC).

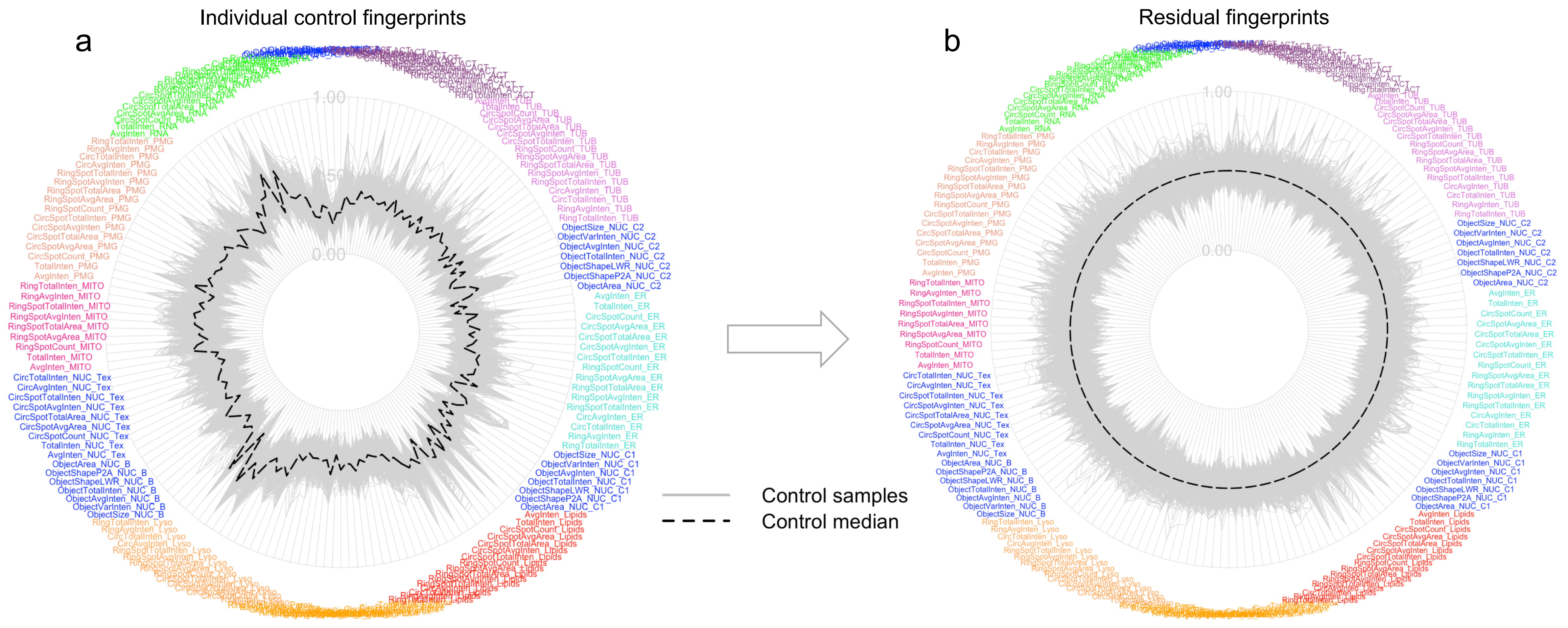

Supplementary Figure 4: Full feature EMD fingerprints of individual control samples.
(**a**) Radial plot of EMD scores spanning 174 measured features among individual controls (gray lines) and the median EMD score of all controls (black dashed line). (**b**) Radial plot of residual EMD scores of individual controls (gray lines) relative to the null control line (black dashed line). Residual score is defined as the difference between the score of the individual control and the median of all controls. Residual fingerprints naturally fluctuate around median zero (black dashed line), here the values have been offset by 0.5 to expand the plot for better visualization.

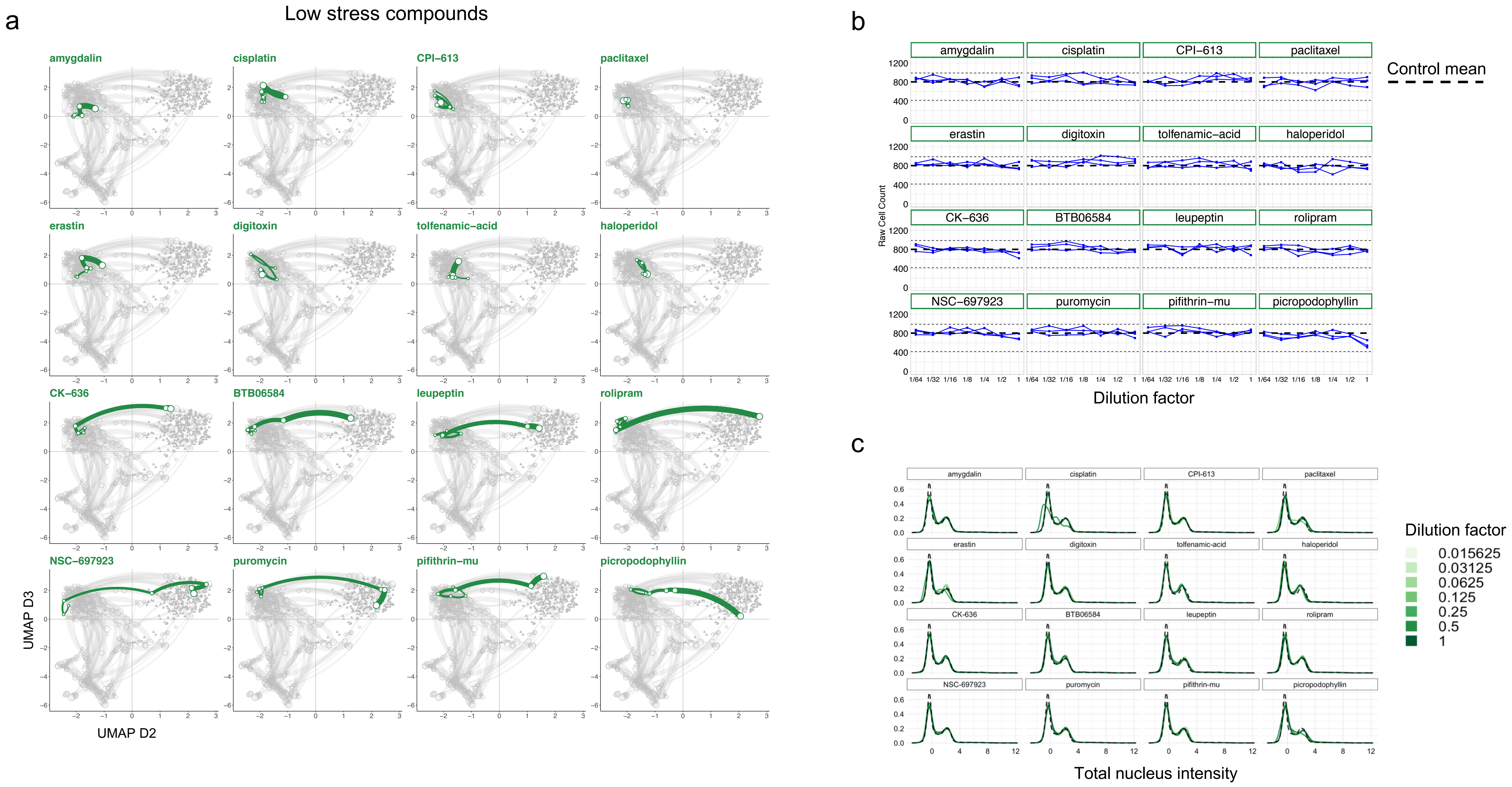

Supplementary Figure 5: Summary of phenotypes for 65 compounds.

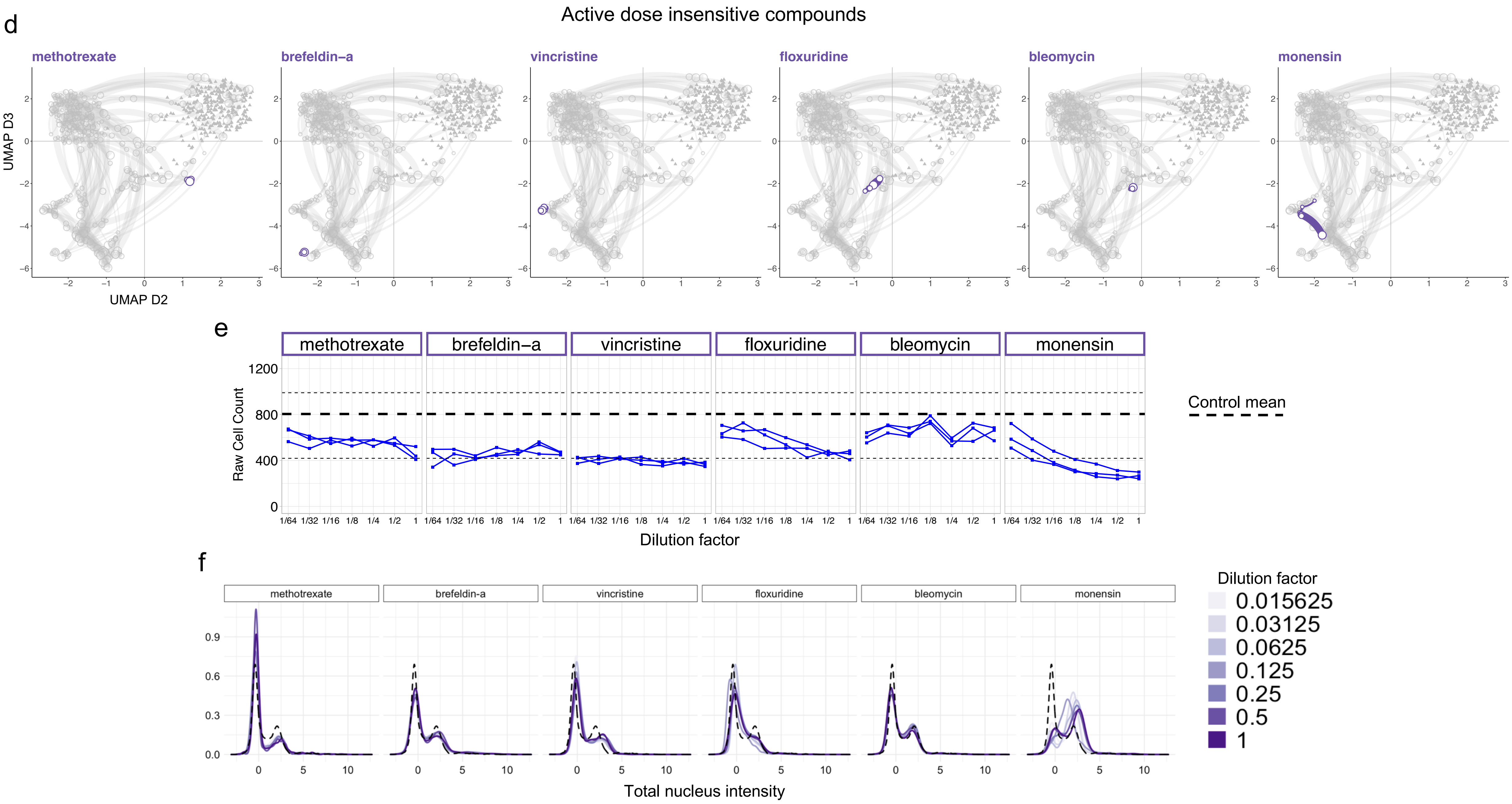

Supplementary Figure 5: Summary of phenotypes for 65 compounds.

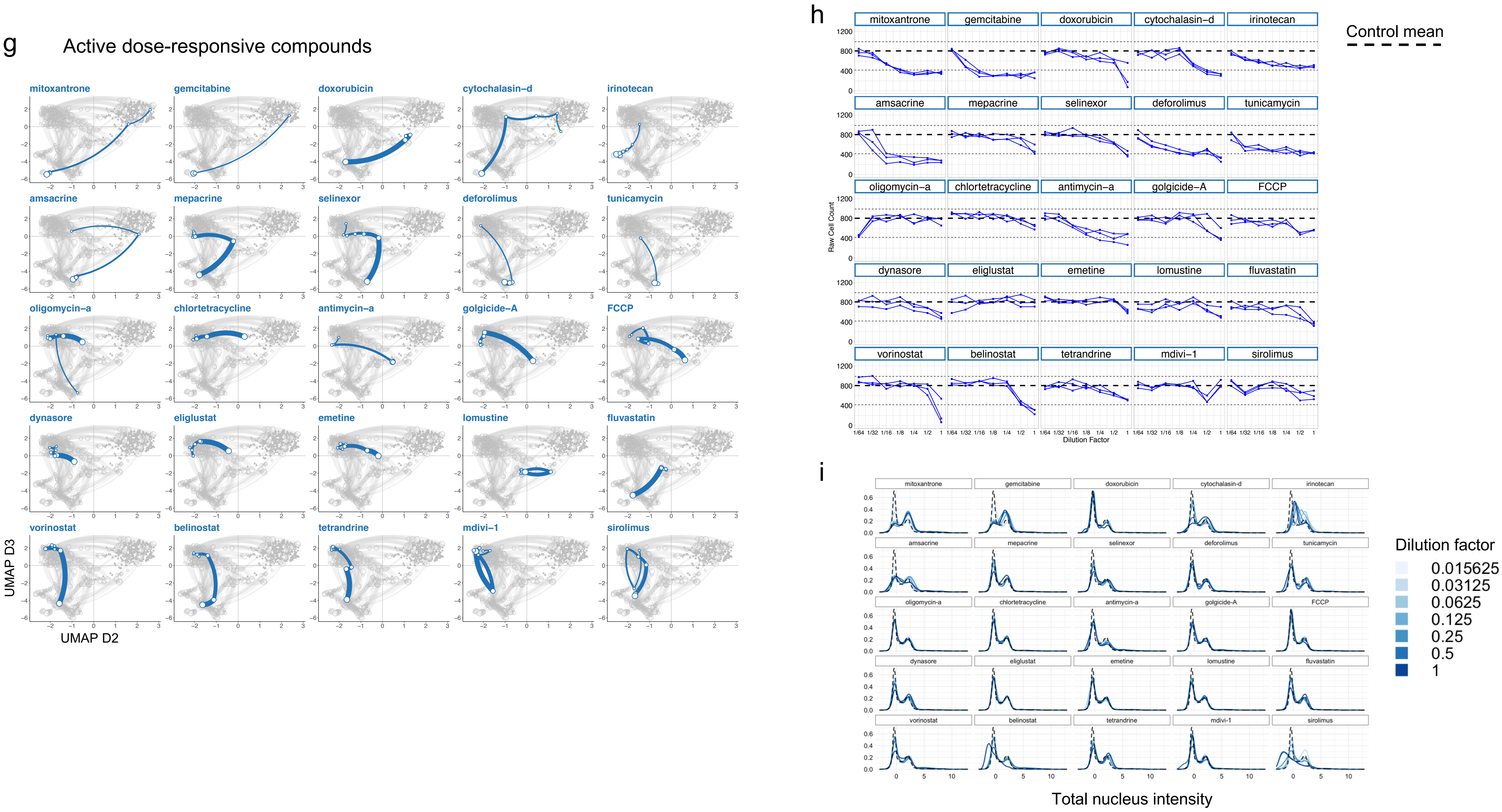

Supplementary Figure 5: Summary of phenotypes for 65 compounds.

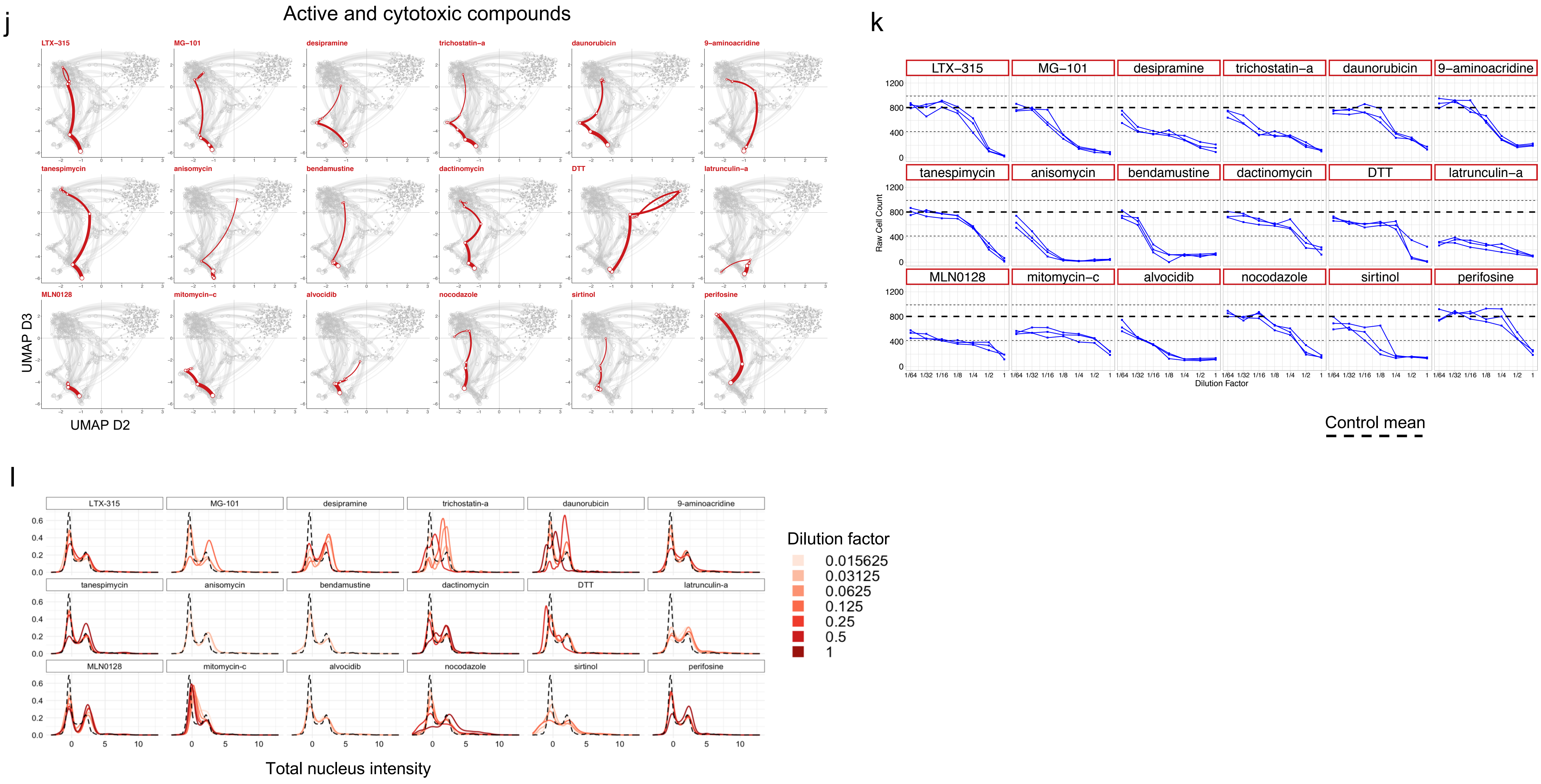

Supplementary Figure 5: Summary of phenotypes for 65 compounds.

Supplementary Figure 5: Summary of phenotypes for 65 compounds.

(**a** – **c**) 16 compounds elicited little bioactivity in U2OS cells. (**a**) Phenotypic trajectories of the “low stress” group. Profiles for some compounds at higher concentrations are not well separated from controls in the first 3 UMAP dimensions. The 16 compounds showed little or no effect on cell counts (**b**) and cell cycle distributions (**c**) across concentration gradients. In (**b**), each curve represents a replicate and three dashed lines represent max, min, and mean of control cell counts. In (**c**), cell cycle is measured by total nuclear intensity; the global control is shown as a dashed line.

(**d** – **f**) 6 compounds with diverse MOA induce phenotypic activity largely independent of dosage/concentration. (**d**) Phenotypic trajectories of the “active (dose-insensitive)” group. These compounds show a distinctive phenotype from the control and low stress group, and the phenotypic response varies little with dosage. These compounds show mildly reduced cell counts in comparison to the control (**e**) and elicit differing effects on the cell cycle (**f**). Visual cues for (**e**, **f**) are as in (**b**, **c**).

(**g** – **i**) 25 compounds elicit dose-dependent phenotypes and are characterized as the “active (dose-responsive)” group. (**g**) Phenotypic trajectories of these compounds travel from the “low stress” region to other areas in the UMAP. Some, but not all, compounds show concentration-dependent effects on cell counts (**h**) and cell cycle responses (**i**). Visual cues for (**h**, **i**) are as in (**b**, **c**).

(**j** – **l**) 18 compounds show a dose-dependent phenotypic change and become toxic at high concentrations. (**j**) Phenotypic trajectories of the “active and cytotoxic” group. These compounds show strong decreases in cell counts with increasing concentration (**k**) and elicit diverse cell cycle responses (**l**). Due to extreme cytotoxicity, cell cycle distributions are not available for some compounds at higher concentrations. Visual cues for (**k**, **l**) are as in (**b**, **c**).

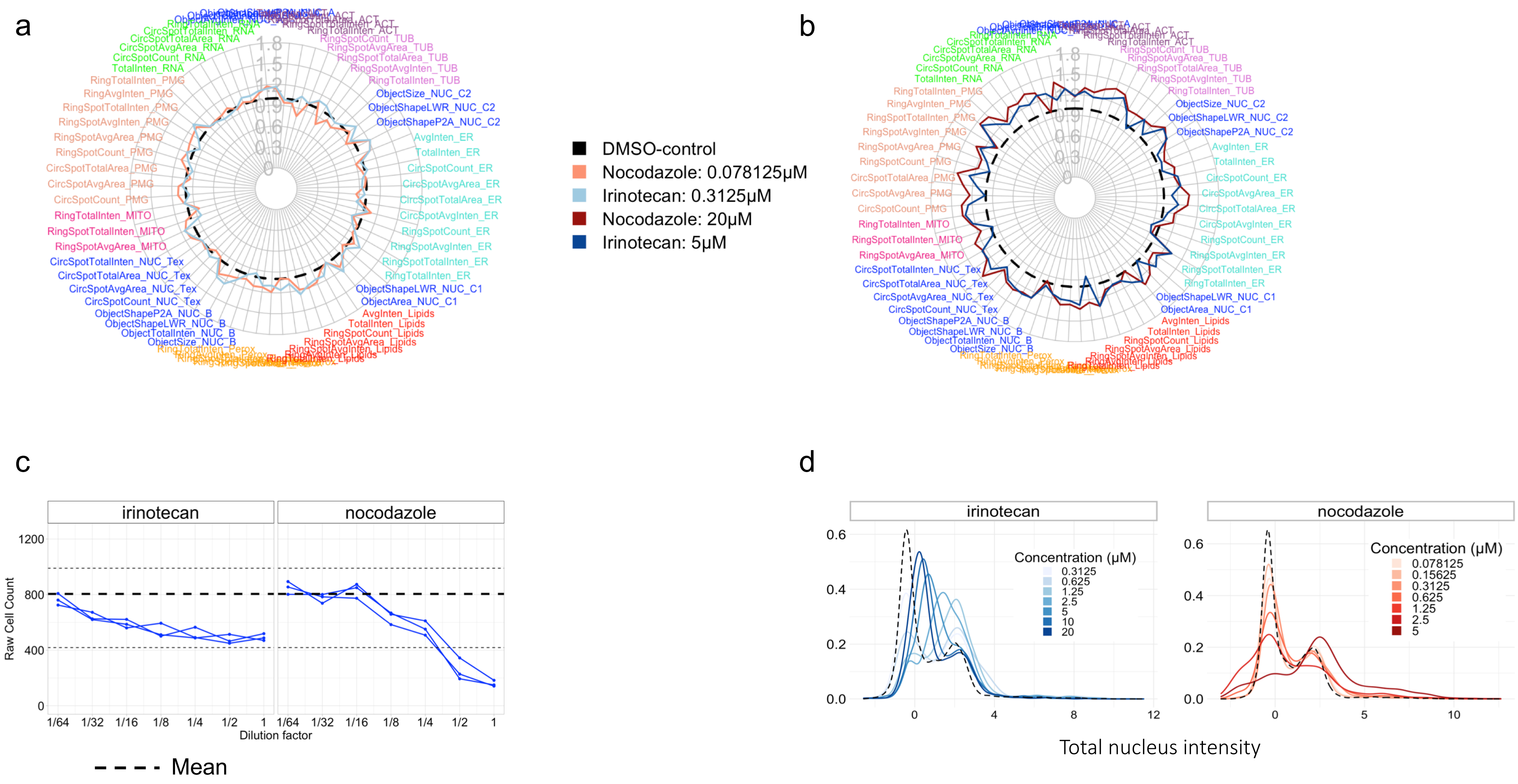

Supplementary Figure 6: Divergent phenotypes.

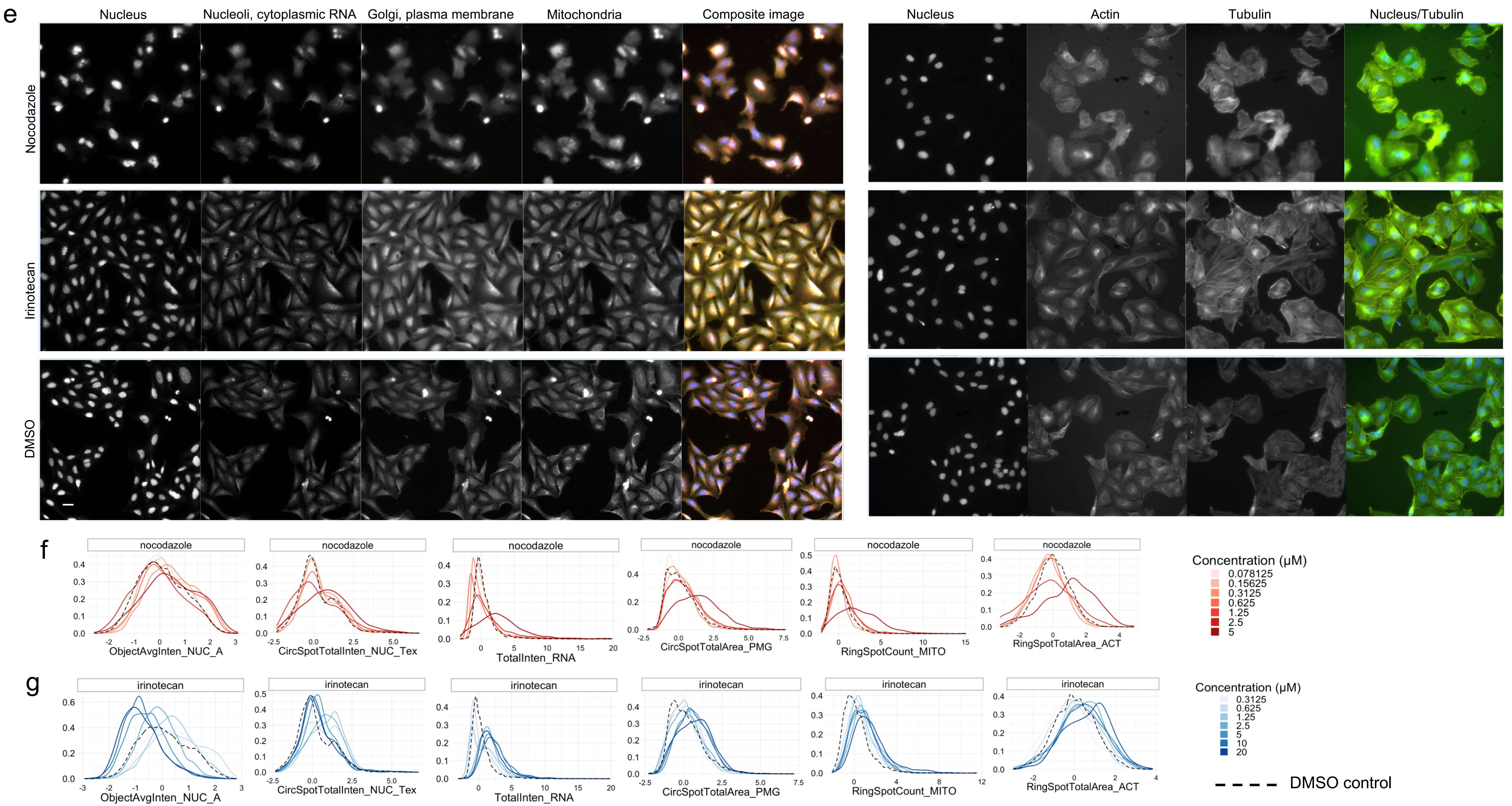

Supplementary Figure 6: Divergent phenotypes.

Supplementary Figure 6: Divergent phenotypes.
Phenotypic fingerprint of both Nocodazole and Irinotecan superimposed at their (**a**) lowest and (**b**) highest concentrations. (**c**) Cell count and (**d**) cell cycle dose response reveal increased levels of stress across the concentration gradient, with diverse cell cycle effects. Cell count is measured from three replicate plates, dashed lines represent the max, min and mean of the DMSO control. (**e**) Raw biological images of cells treated with 20uM of Nocodazole (top row), 5uM of Irinotecan (middle row), and DMSO (bottom row). Columns display a selection of channels imaged in the multi-panel assay. Scale bar: 20 μm. (**f**) Cell feature distributions display diverse phenotypic responses to Nocodazole and (**g**) Irinotecan.

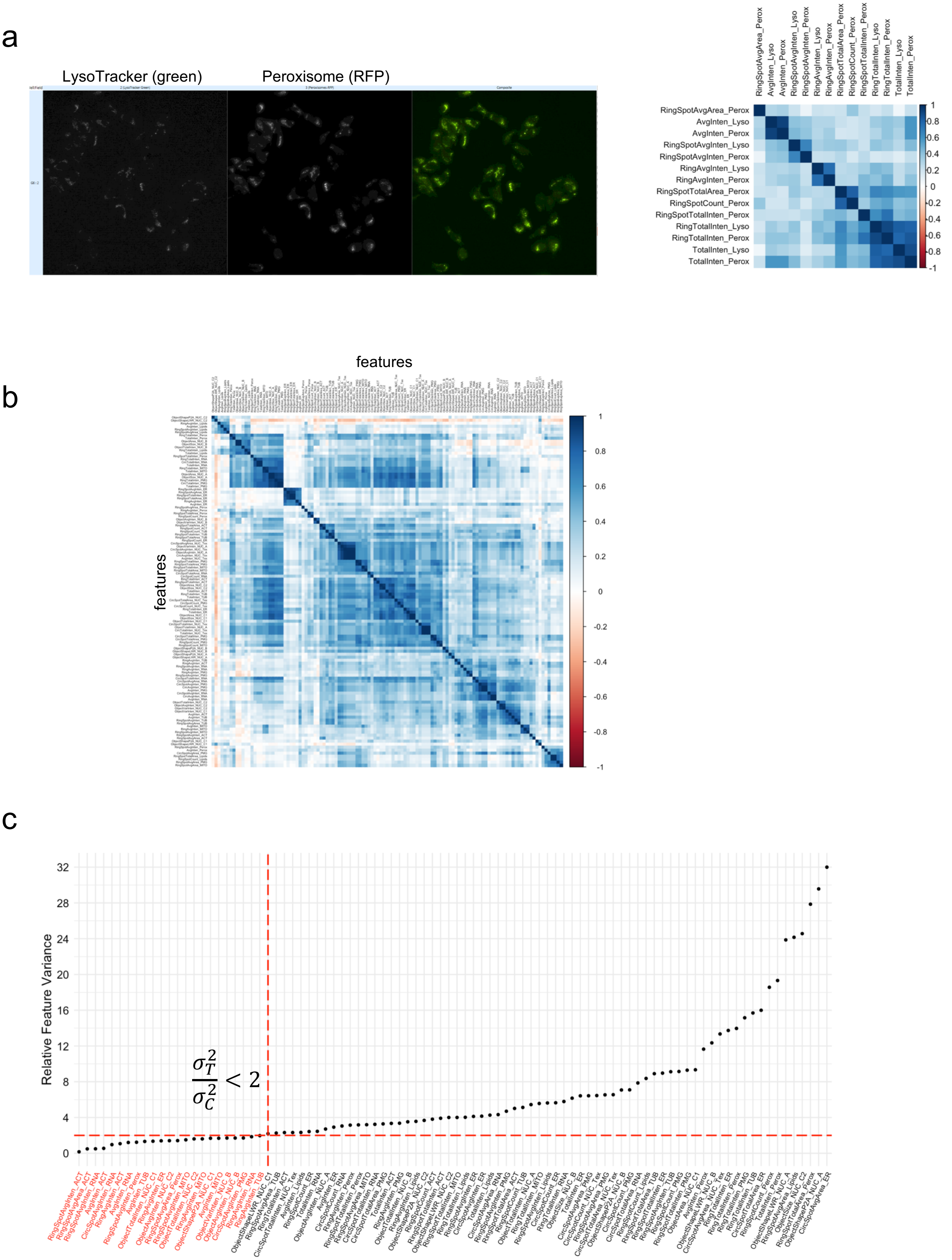

Supplementary Figure 7. Feature reduction.

Supplementary Figure 7. Feature reduction.
(**a**) The lysosome and peroxisome reporters are highly correlated due to overlap in their fluorescent emission spectra arising from the weak lysosomal staining by LysoTracker Green. The right-hand panel shows the linear correlation between the parameters of these two channels. (**b**) Linear correlation analysis of all remaining features. For any pair of highly correlated features (correlation coefficient > 0.9), the feature showing a larger mean correlation with all other features is removed. (**c**) Relative feature activity: per-feature variance is calculated for both control and treatment samples. Features whose variance among treatments is less than twice the variance of the control are deemed “inactive”.

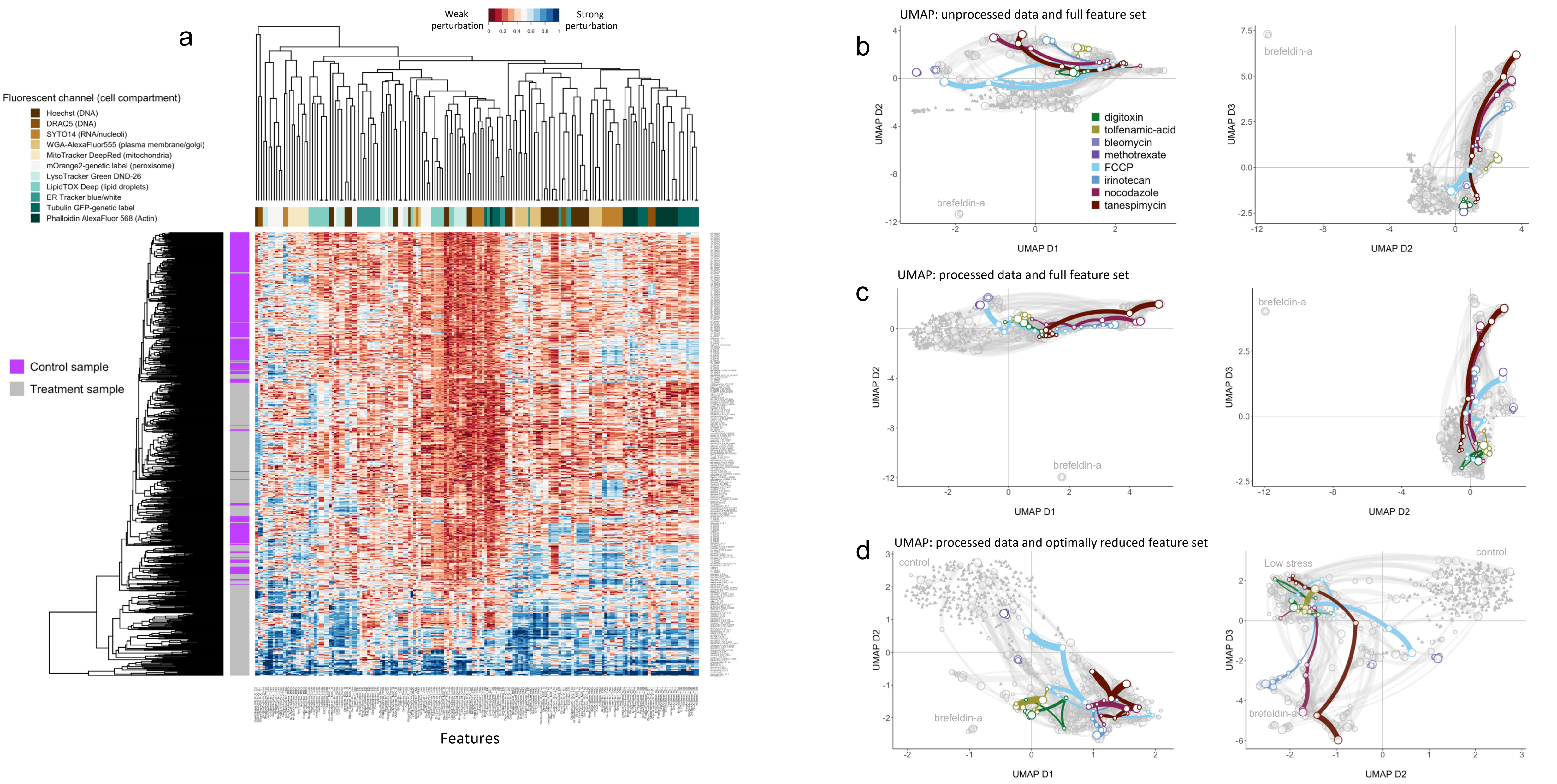

Supplementary Figure 8: Comparison of cytological profiles

(**a - b**) EMD profiles without adjusting for positional effects and plate to plate variation. (**a**) Heatmap visualization of similarity by hierarchical clustering shows several treatment groups (grey) clustered with the controls (purple). Dimension reduction by UMAP of (**b**) unprocessed EMD profiles and (**c**) processed full feature EMD profiles strongly separates the brefeldin-a cluster from other treatments. (**c**) UMAP of processed full feature profile also fails to separate the low stress cluster from the control when compared to the (**d**) UMAP of the processed and optimally reduced feature profile (described in Fig. 7a).

Supplementary Figure 9: Radial plot fingerprints of 65 compounds
Radial fingerprints of residual EMD scores for 65 compounds at multiple concentrations, relative to the control median (black dotted line). Labels for the reduced 69 feature set are color-coded by corresponding cellular marker. Per compound legends include compound name, concentration and dilution factor (*compound_concentration_dilution factor*). Lowest and highest concentrations are colored yellow and dark purple respectively.

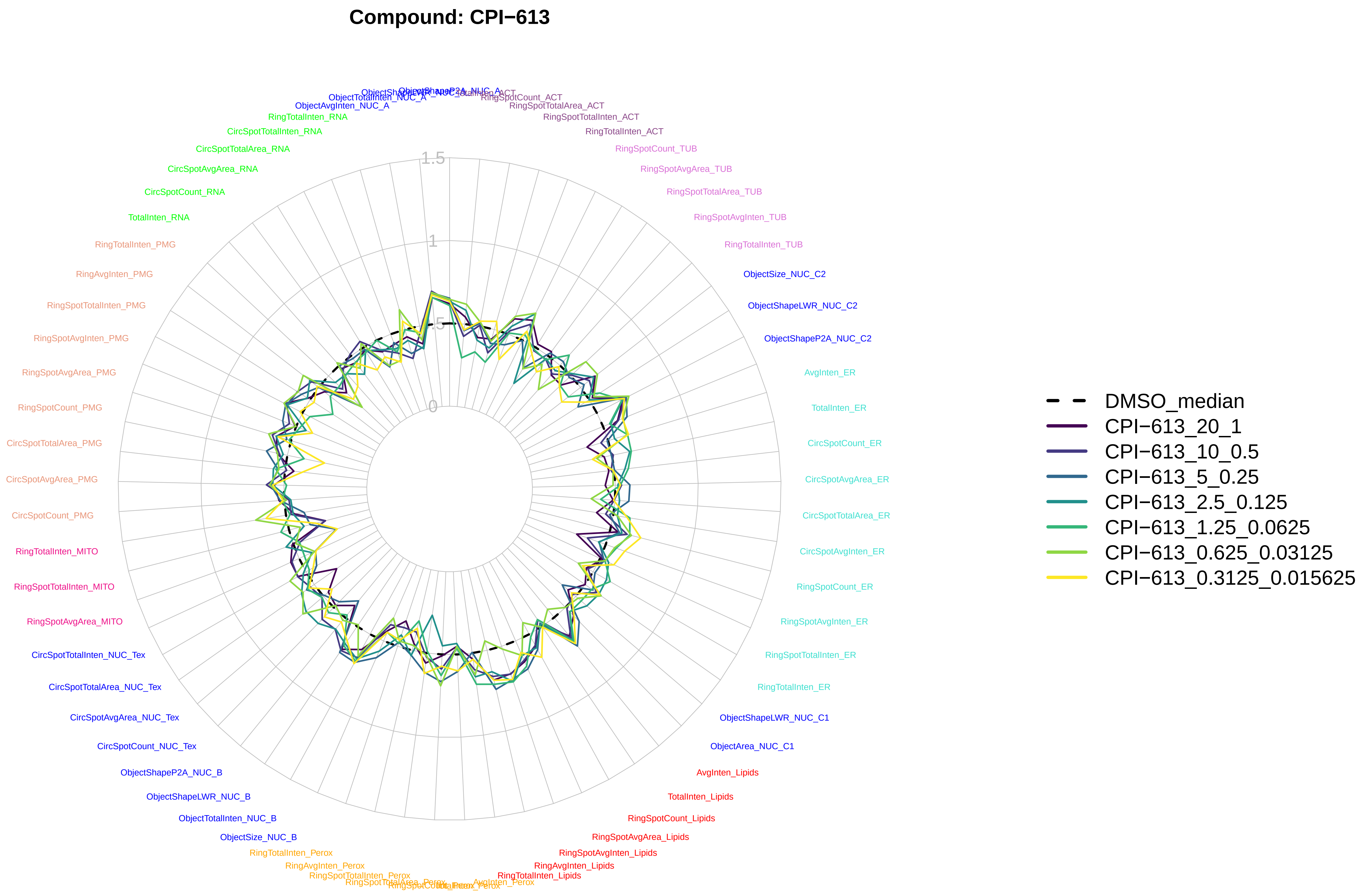

Compound: CPI−613
ObjectTotalInten_NUC_A
ObjectAvgInten_NUC_A
RingTotalInten_RNA
CircSpotTotalInten_RNA
CircSpotTotalArea_RNA
CircSpotAvgArea_RNA
CircSpotCount_RNA
TotalInten_RNA
RingTotalInten_PMG
RingAvgInten_PMG
RingSpotTotalInten_PMG
RingSpotAvgInten_PMG
RingSpotAvgArea_PMG
RingSpotCount_PMG
CircSpotTotalArea_PMG
CircSpotAvgArea_PMG
CircSpotCount_PMG
RingTotalInten_MITO
RingSpotTotalInten_MITO
RingSpotAvgArea_MITO
CircSpotTotalInten_NUC_Tex
CircSpotTotalArea_NUC_Tex
CircSpotAvgArea_NUC_Tex
CircSpotCount_NUC_Tex
ObjectShapeP2A_NUC_B
ObjectShapeLWR_NUC_B
ObjectTotalInten_NUC_B
ObjectSize_NUC_B
RingTotalInten_Perox
RingAvgInten_Perox
RingSpotTotalInten_Perox
AvgInten_Perox
RingTotalInten_Lipids
RingAvgInten_Lipids
RingSpotAvgInten_Lipids
RingSpotAvgArea_Lipids
RingSpotCount_Lipids
TotalInten_Lipids
AvgInten_Lipids
ObjectArea_NUC_C1
ObjectShapeLWR_NUC_C1
RingTotalInten_ER
RingSpotTotalInten_ER
RingSpotAvgInten_ER
RingSpotCount_ER
CircSpotAvgInten_ER
CircSpotTotalArea_ER
CircSpotAvgArea_ER
CircSpotCount_ER
TotalInten_ER
AvgInten_ER
ObjectShapeP2A_NUC_C2
ObjectShapeLWR_NUC_C2
ObjectSize_NUC_C2
RingTotalInten_TUB
RingSpotAvgInten_TUB
RingSpotTotalArea_TUB
RingSpotAvgArea_TUB
RingSpotCount_TUB
RingTotalInten_ACT
RingSpotTotalInten_ACT
RingSpotTotalArea_ACT
RingSpotCount_ACT
1.5
1
0
DMSO_median
CPI−613_20_1
CPI−613_10_0.5
CPI−613_5_0.25
CPI−613_2.5_0.125
CPI−613_1.25_0.0625
CPI−613_0.625_0.03125
CPI−613_0.3125_0.015625

Compound: digitoxin

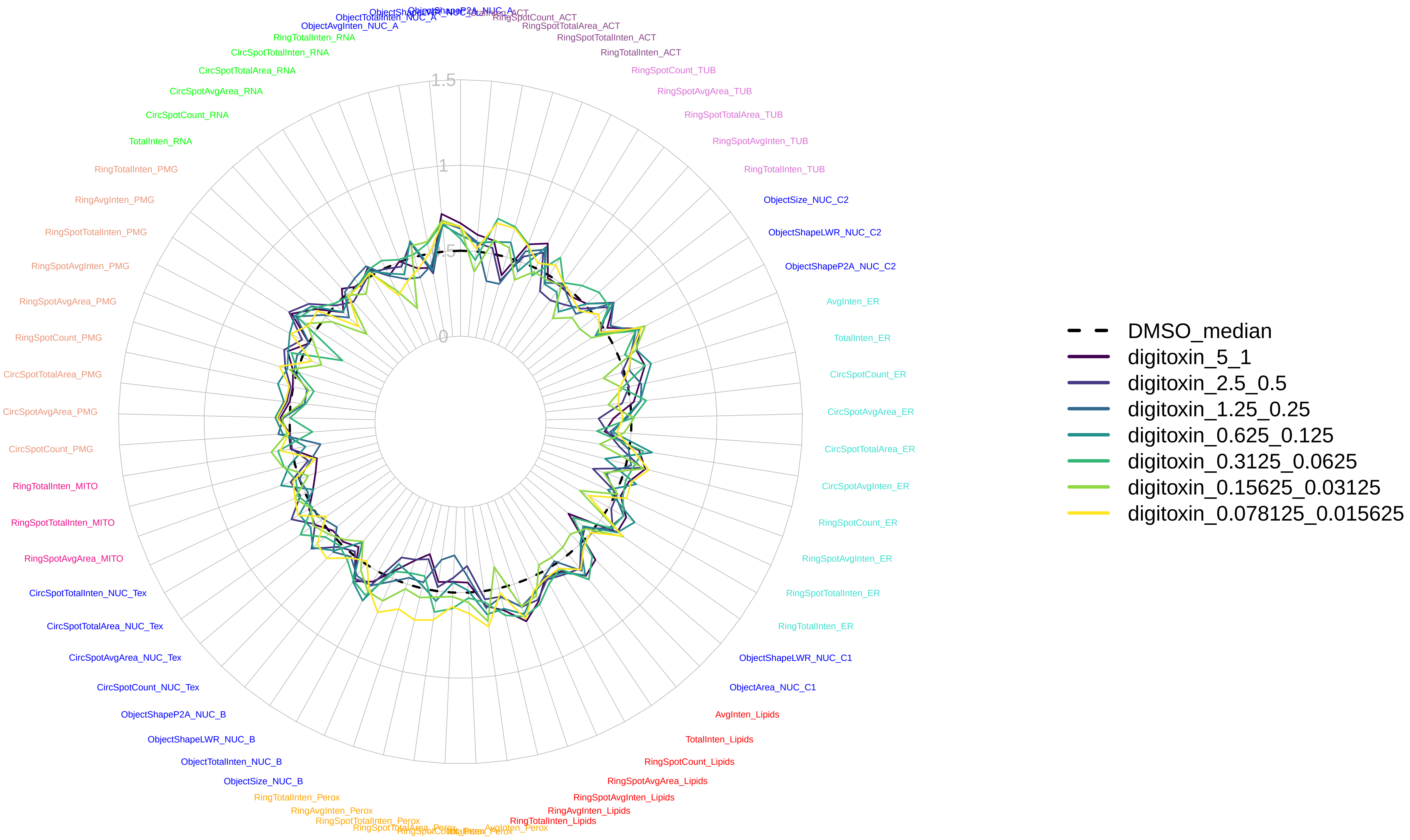

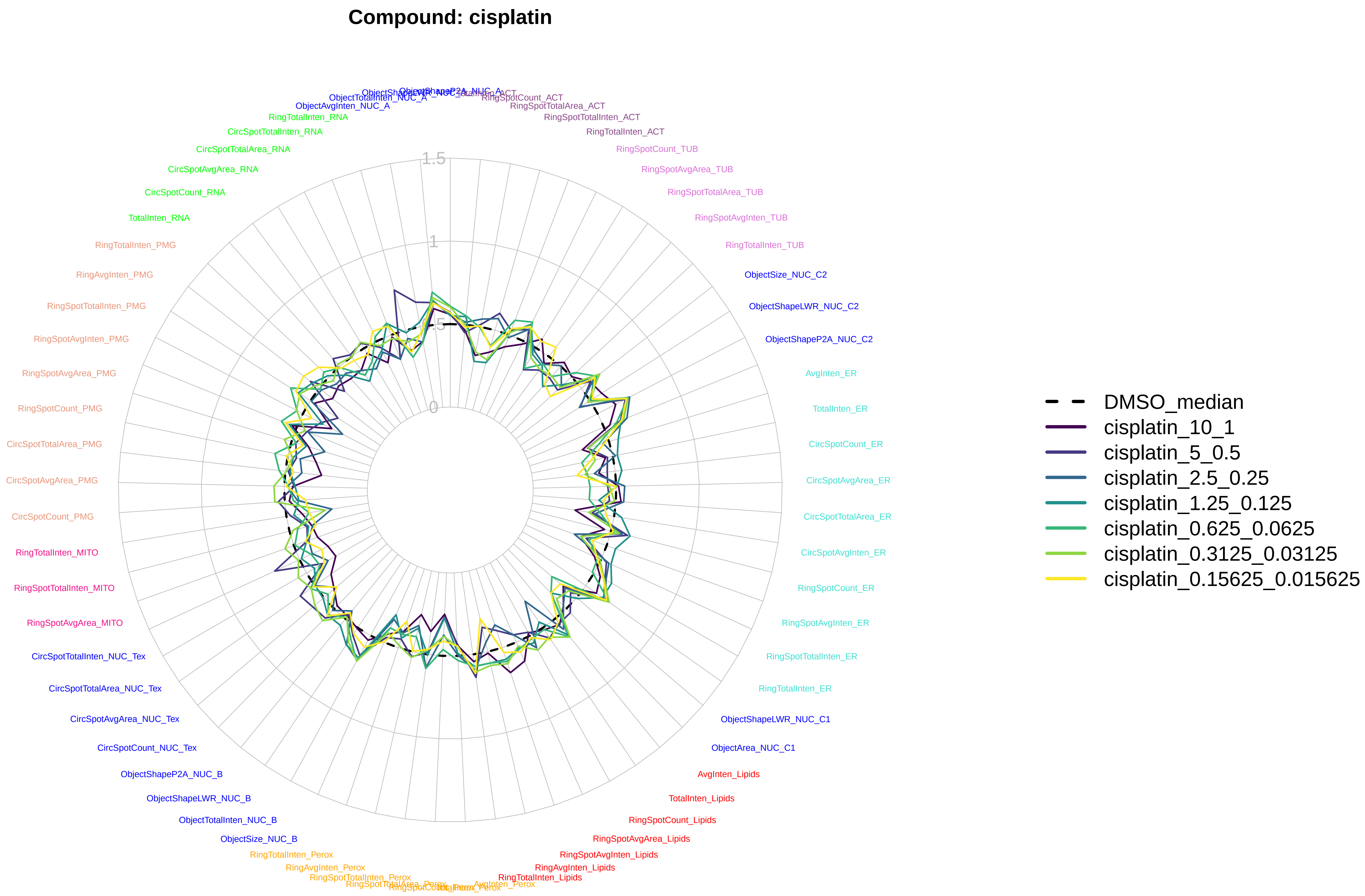
Compound: cisplatin
ObjectAvgInten_NUC_A
RingTotalInten_RNA
CircSpotTotalInten_RNA
CircSpotTotalArea_RNA
CircSpotAvgArea_RNA
CircSpotCount_RNA
TotalInten_RNA
RingTotalInten_PMG
RingAvgInten_PMG
RingSpotTotalInten_PMG
RingSpotAvgInten_PMG
RingSpotAvgArea_PMG
RingSpotCount_PMG
CircSpotTotalArea_PMG
CircSpotAvgArea_PMG
CircSpotCount_PMG
RingTotalInten_MITO
RingSpotTotalInten_MITO
RingSpotAvgArea_MITO
CircSpotTotalInten_NUC_Tex
CircSpotTotalArea_NUC_Tex
CircSpotAvgArea_NUC_Tex
CircSpotCount_NUC_Tex
ObjectShapeP2A_NUC_B
ObjectShapeLWR_NUC_B
ObjectTotalInten_NUC_B
ObjectSize_NUC_B
RingTotalInten_Perox
RingAvgInten_Perox
RingSpotCount_ACT
RingSpotTotalArea_ACT
RingSpotTotalInten_ACT
RingTotalInten_ACT
RingSpotCount_TUB
RingSpotAvgArea_TUB
RingSpotTotalArea_TUB
RingSpotAvgInten_TUB
RingTotalInten_TUB
ObjectSize_NUC_C2
ObjectShapeLWR_NUC_C2
ObjectShapeP2A_NUC_C2
AvgInten_ER
TotalInten_ER
CircSpotCount_ER
CircSpotAvgArea_ER
CircSpotTotalArea_ER
CircSpotAvgInten_ER
RingSpotCount_ER
RingSpotAvgInten_ER
RingSpotTotalInten_ER
RingTotalInten_ER
ObjectShapeLWR_NUC_C1
ObjectArea_NUC_C1
AvgInten_Lipids
TotalInten_Lipids
RingSpotCount_Lipids
RingSpotAvgArea_Lipids
RingSpotAvgInten_Lipids
RingAvgInten_Lipids
RingTotalInten_Lipids
1.5
1
.5
0
DMSO_median
cisplatin_10_1
cisplatin_5_0.5
cisplatin_2.5_0.25
cisplatin_1.25_0.125
cisplatin_0.625_0.0625
cisplatin_0.3125_0.03125
cisplatin_0.15625_0.015625

Compound: 9−aminoacridine

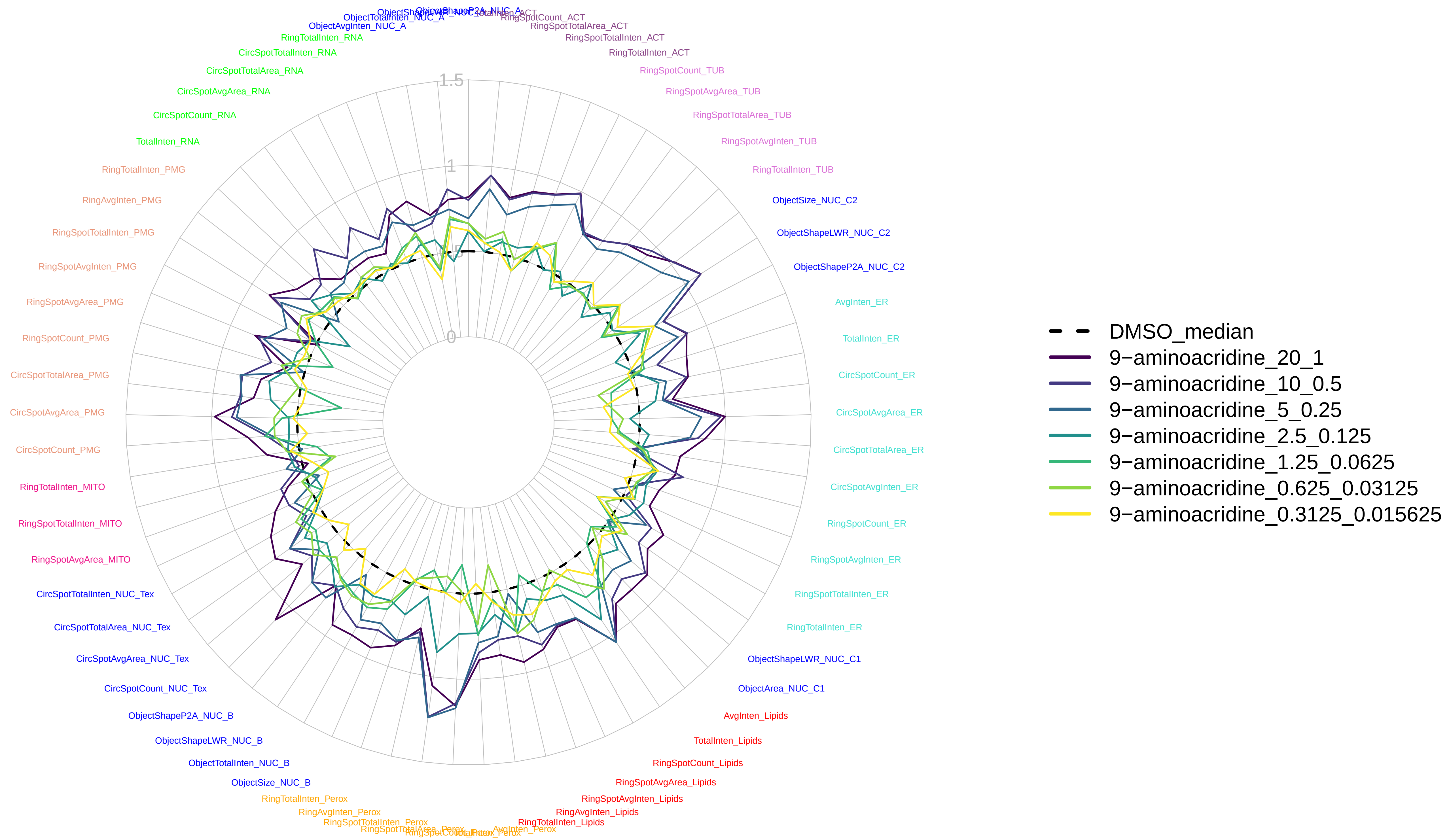

Compound: chlortetracycline

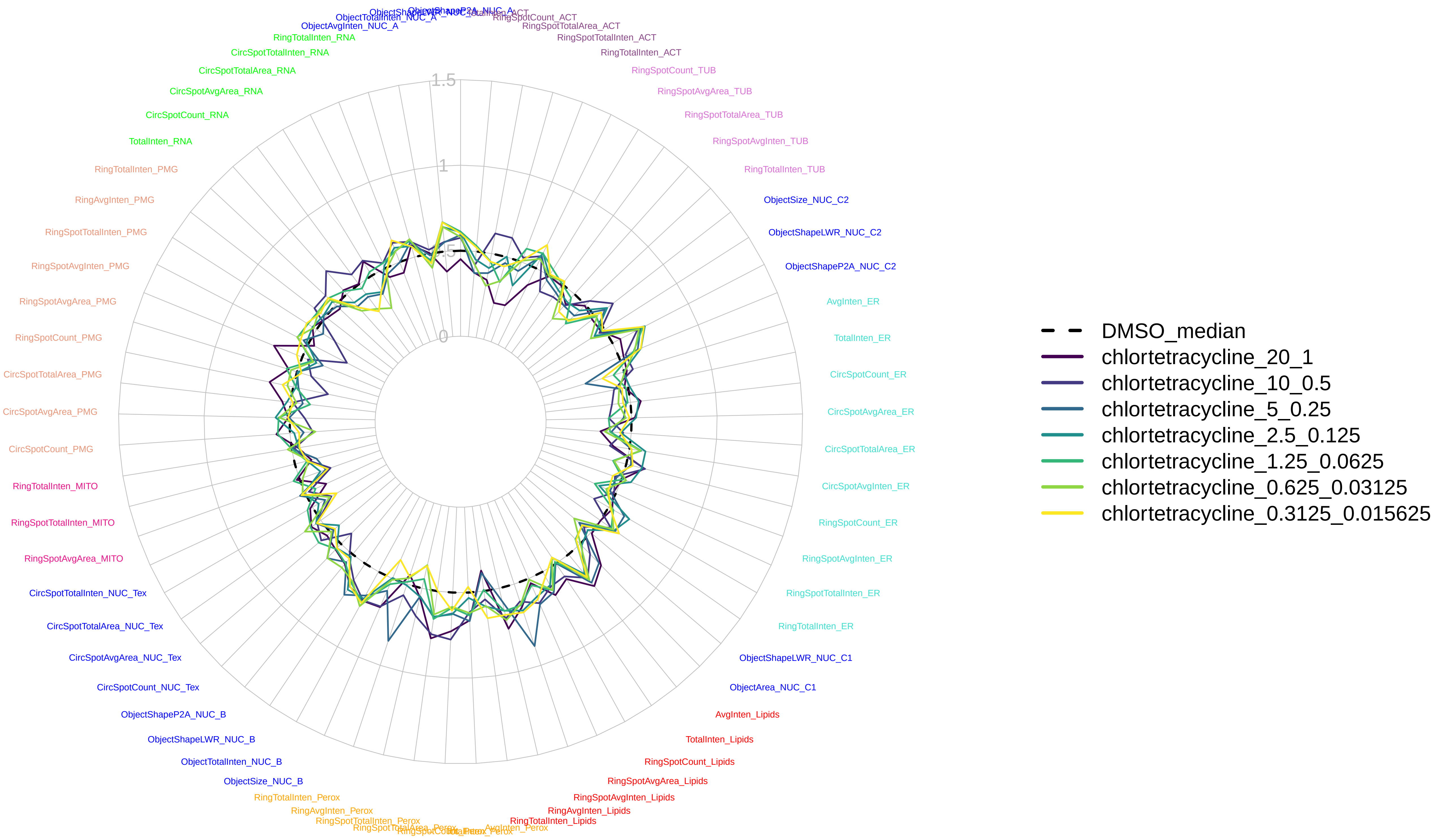

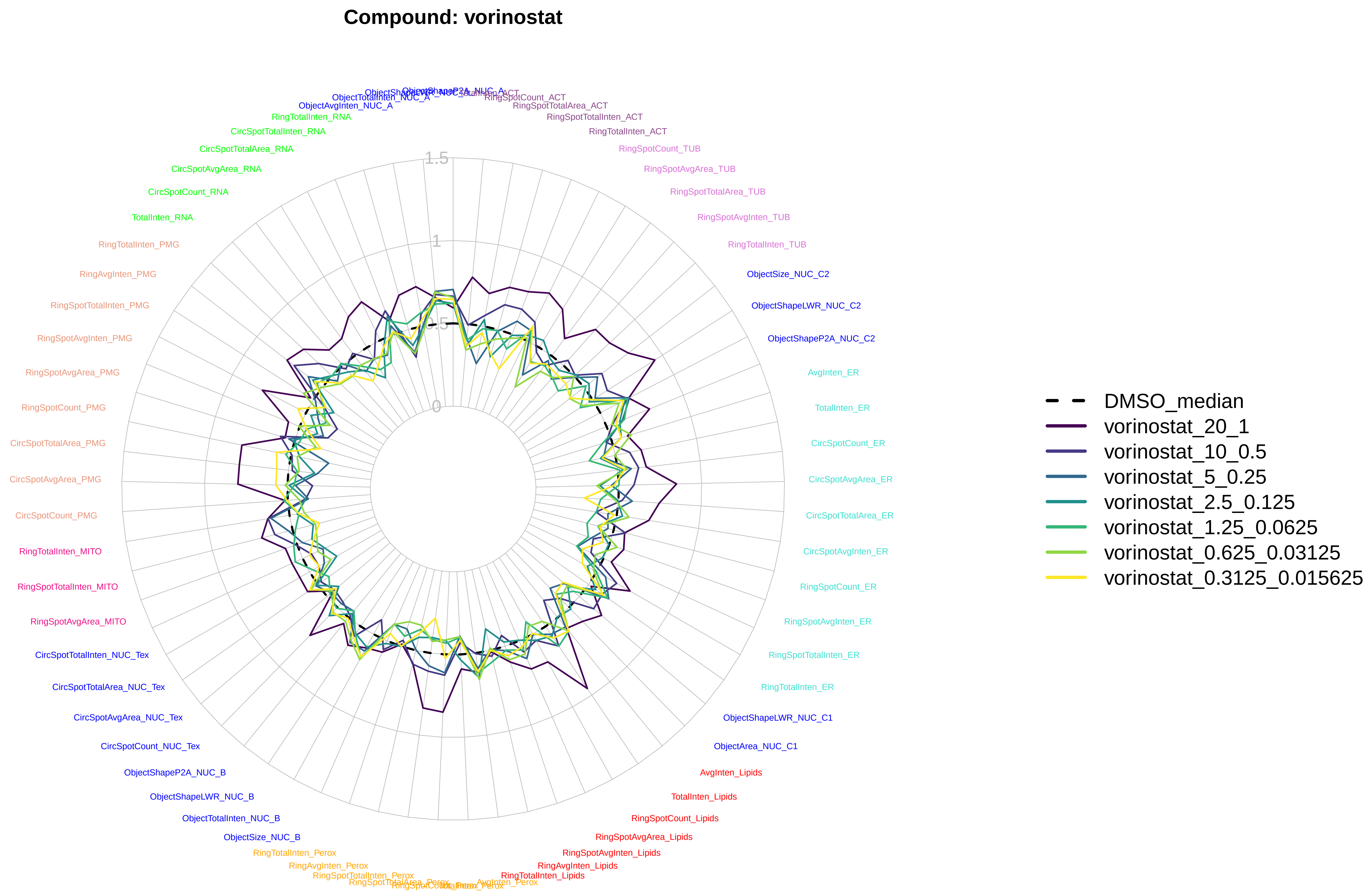

Compound: vorinostat
ObjectTotalInten_NUC_A
ObjectAvgInten_NUC_A
RingSpotCount_ACT
RingSpotTotalArea_ACT
RingSpotTotalInten_ACT
RingTotalInten_ACT
RingSpotCount_TUB
RingSpotAvgArea_TUB
RingSpotTotalArea_TUB
RingSpotAvgInten_TUB
RingTotalInten_TUB
ObjectSize_NUC_C2
ObjectShapeLWR_NUC_C2
ObjectShapeP2A_NUC_C2
AvgInten_ER
TotalInten_ER
CircSpotCount_ER
CircSpotAvgArea_ER
CircSpotTotalArea_ER
CircSpotAvgInten_ER
RingSpotCount_ER
RingSpotAvgInten_ER
RingSpotTotalInten_ER
RingTotalInten_ER
ObjectShapeLWR_NUC_C1
ObjectArea_NUC_C1
AvgInten_Lipids
TotalInten_Lipids
RingSpotCount_Lipids
RingSpotAvgArea_Lipids
RingSpotAvgInten_Lipids
RingAvgInten_Lipids
RingTotalInten_Lipids
AvgInten_Perox
RingSpotTotalArea_Perox
RingSpotTotalInten_Perox
RingAvgInten_Perox
RingTotalInten_Perox
ObjectSize_NUC_B
ObjectTotalInten_NUC_B
ObjectShapeLWR_NUC_B
ObjectShapeP2A_NUC_B
CircSpotCount_NUC_Tex
CircSpotAvgArea_NUC_Tex
CircSpotTotalArea_NUC_Tex
CircSpotTotalInten_NUC_Tex
RingSpotAvgArea_MITO
RingSpotTotalInten_MITO
RingTotalInten_MITO
CircSpotCount_PMG
CircSpotAvgArea_PMG
CircSpotTotalArea_PMG
RingSpotCount_PMG
RingSpotAvgArea_PMG
RingSpotAvgInten_PMG
RingSpotTotalInten_PMG
RingAvgInten_PMG
RingTotalInten_PMG
TotalInten_RNA
CircSpotCount_RNA
CircSpotAvgArea_RNA
CircSpotTotalArea_RNA
CircSpotTotalInten_RNA
RingTotalInten_RNA
1.5
1
0.5
0
DMSO_median
vorinostat_20_1
vorinostat_10_0.5
vorinostat_5_0.25
vorinostat_2.5_0.125
vorinostat_1.25_0.0625
vorinostat_0.625_0.03125
vorinostat_0.3125_0.015625

Compound: BTB06584

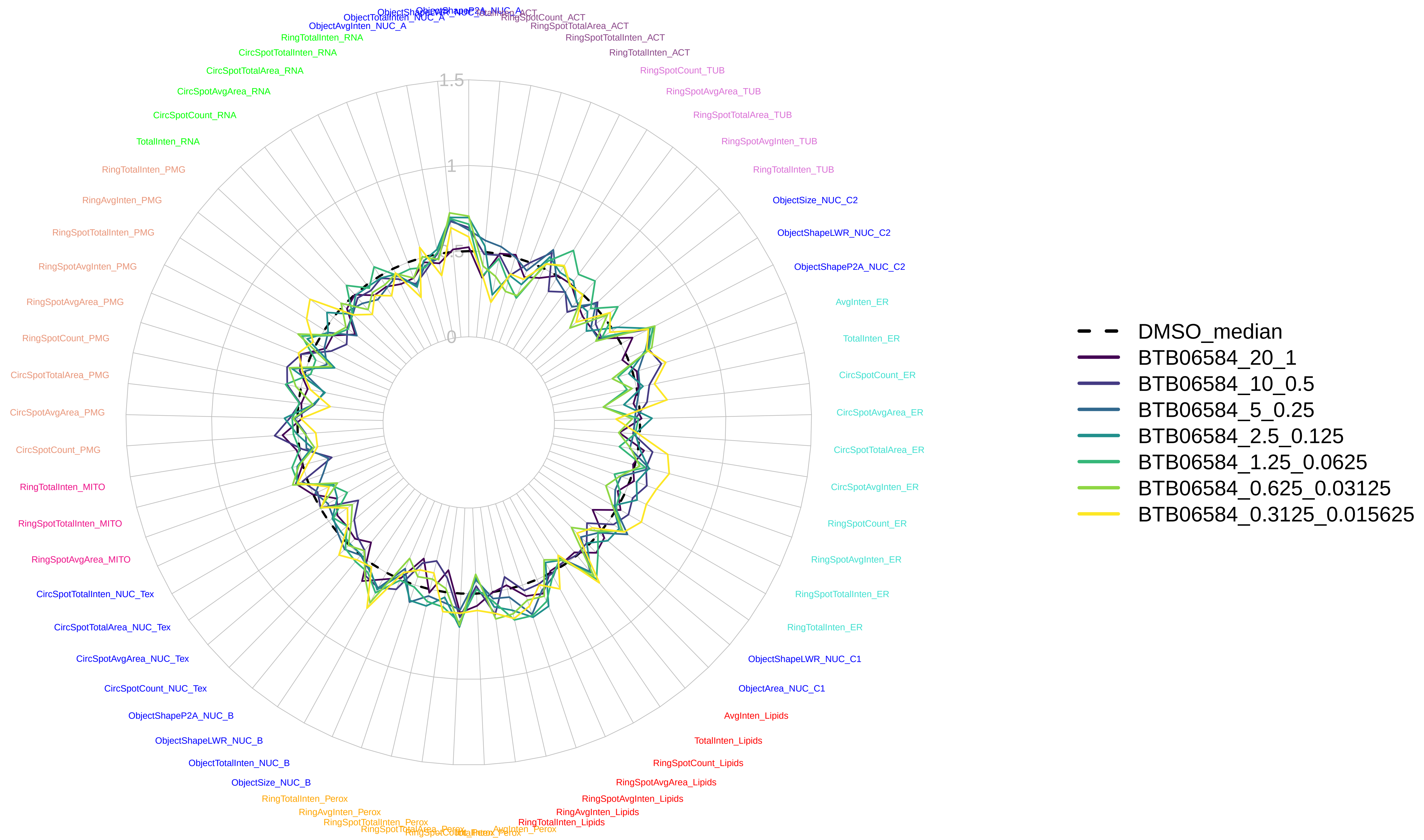

Compound: pifithrin−mu

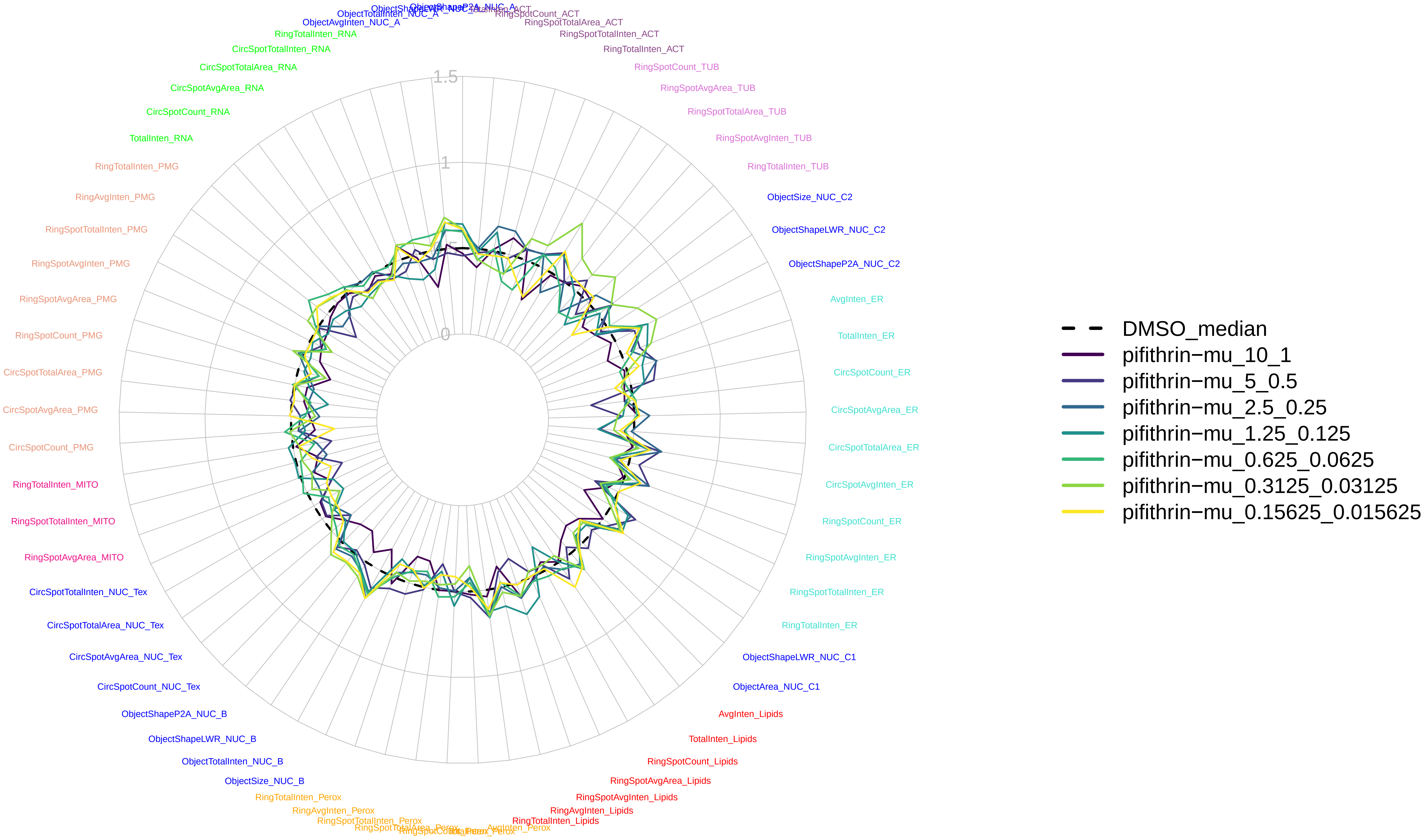

Compound: belinostat

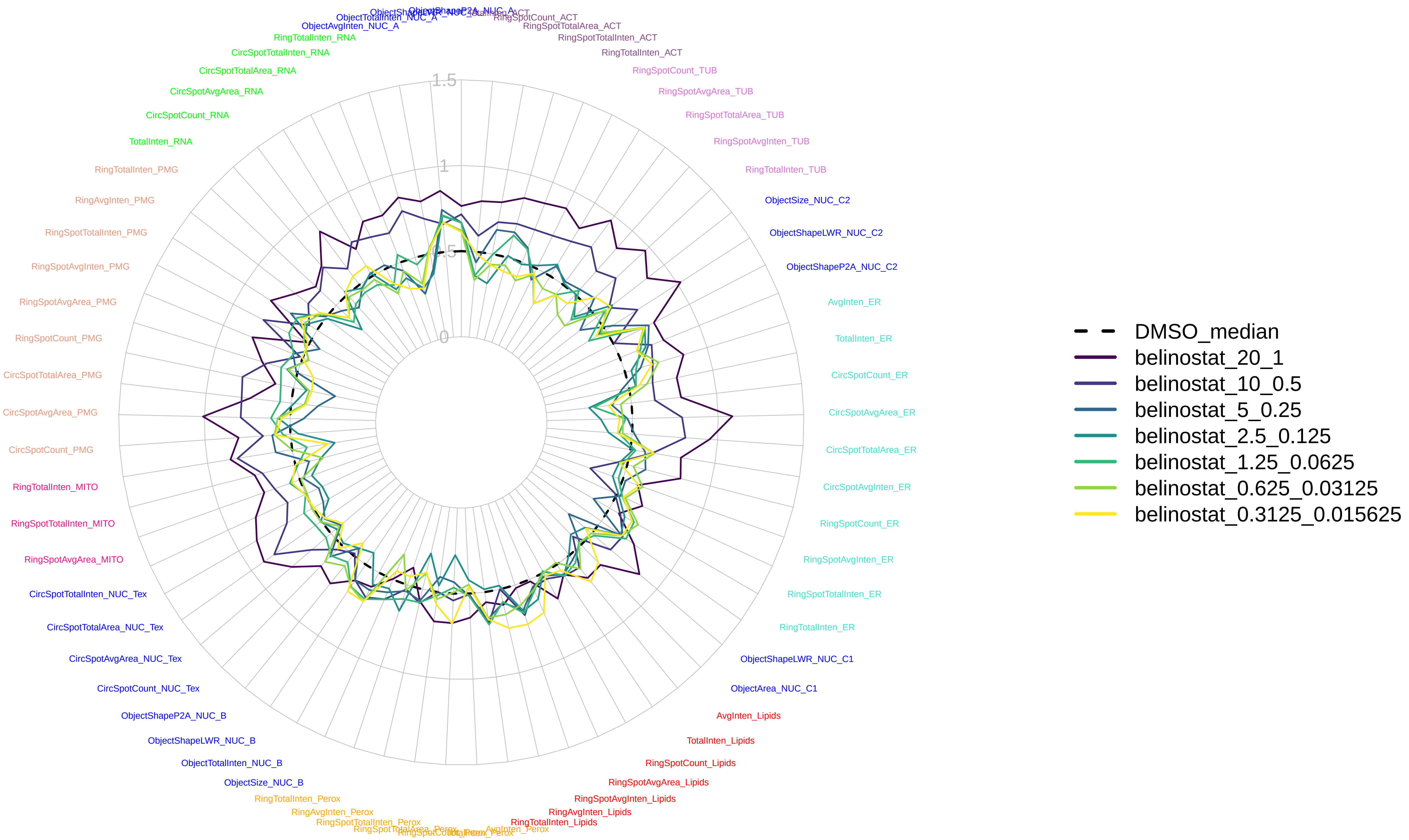

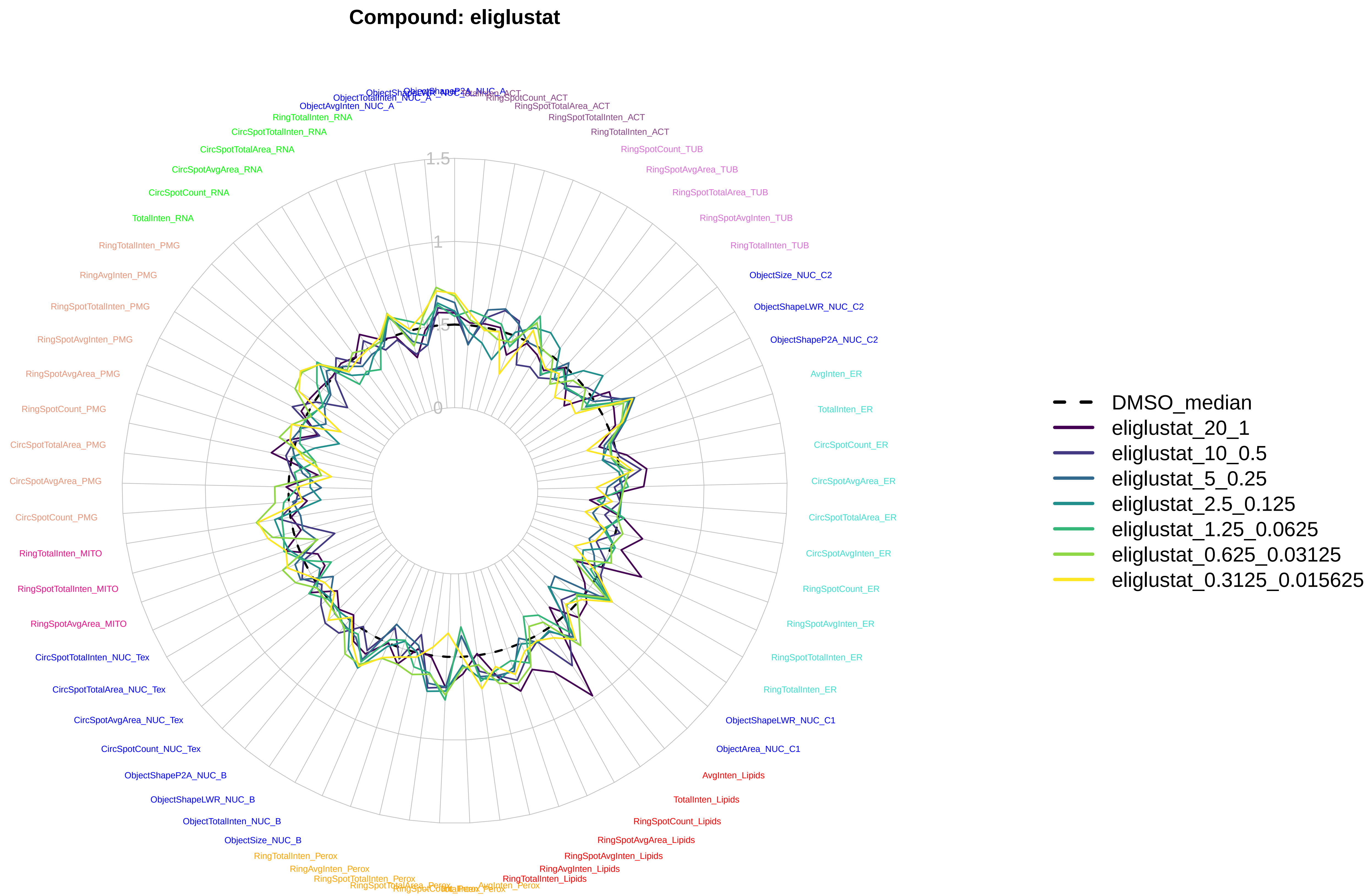

Compound: eliglustat
1.5
1
0.5
0
ObjectShapeP2A_NUC_A
ObjectTotalInten_NUC_A
ObjectAvgInten_NUC_A
RingTotalInten_RNA
CircSpotTotalInten_RNA
CircSpotTotalArea_RNA
CircSpotAvgArea_RNA
CircSpotCount_RNA
TotalInten_RNA
RingTotalInten_PMG
RingAvgInten_PMG
RingSpotTotalInten_PMG
RingSpotAvgInten_PMG
RingSpotAvgArea_PMG
RingSpotCount_PMG
CircSpotTotalArea_PMG
CircSpotAvgArea_PMG
CircSpotCount_PMG
RingTotalInten_MITO
RingSpotTotalInten_MITO
RingSpotAvgArea_MITO
CircSpotTotalInten_NUC_Tex
CircSpotTotalArea_NUC_Tex
CircSpotAvgArea_NUC_Tex
CircSpotCount_NUC_Tex
ObjectShapeP2A_NUC_B
ObjectShapeLWR_NUC_B
ObjectTotalInten_NUC_B
ObjectSize_NUC_B
RingTotalInten_Perox
RingAvgInten_Perox
RingSpotTotalInten_Perox
AvgInten_Perox
RingTotalInten_Lipids
RingAvgInten_Lipids
RingSpotAvgInten_Lipids
RingSpotAvgArea_Lipids
RingSpotCount_Lipids
TotalInten_Lipids
AvgInten_Lipids
ObjectArea_NUC_C1
ObjectShapeLWR_NUC_C1
RingTotalInten_ER
RingSpotTotalInten_ER
RingSpotAvgInten_ER
RingSpotCount_ER
CircSpotAvgInten_ER
CircSpotTotalArea_ER
CircSpotAvgArea_ER
CircSpotCount_ER
TotalInten_ER
AvgInten_ER
ObjectShapeP2A_NUC_C2
ObjectShapeLWR_NUC_C2
ObjectSize_NUC_C2
RingTotalInten_TUB
RingSpotAvgInten_TUB
RingSpotTotalArea_TUB
RingSpotAvgArea_TUB
RingSpotCount_TUB
RingTotalInten_ACT
RingSpotTotalInten_ACT
RingSpotTotalArea_ACT
RingSpotCount_ACT
DMSO_median
eliglustat_20_1
eliglustat_10_0.5
eliglustat_5_0.25
eliglustat_2.5_0.125
eliglustat_1.25_0.0625
eliglustat_0.625_0.03125
eliglustat_0.3125_0.015625

Compound: tolfenamic−acid

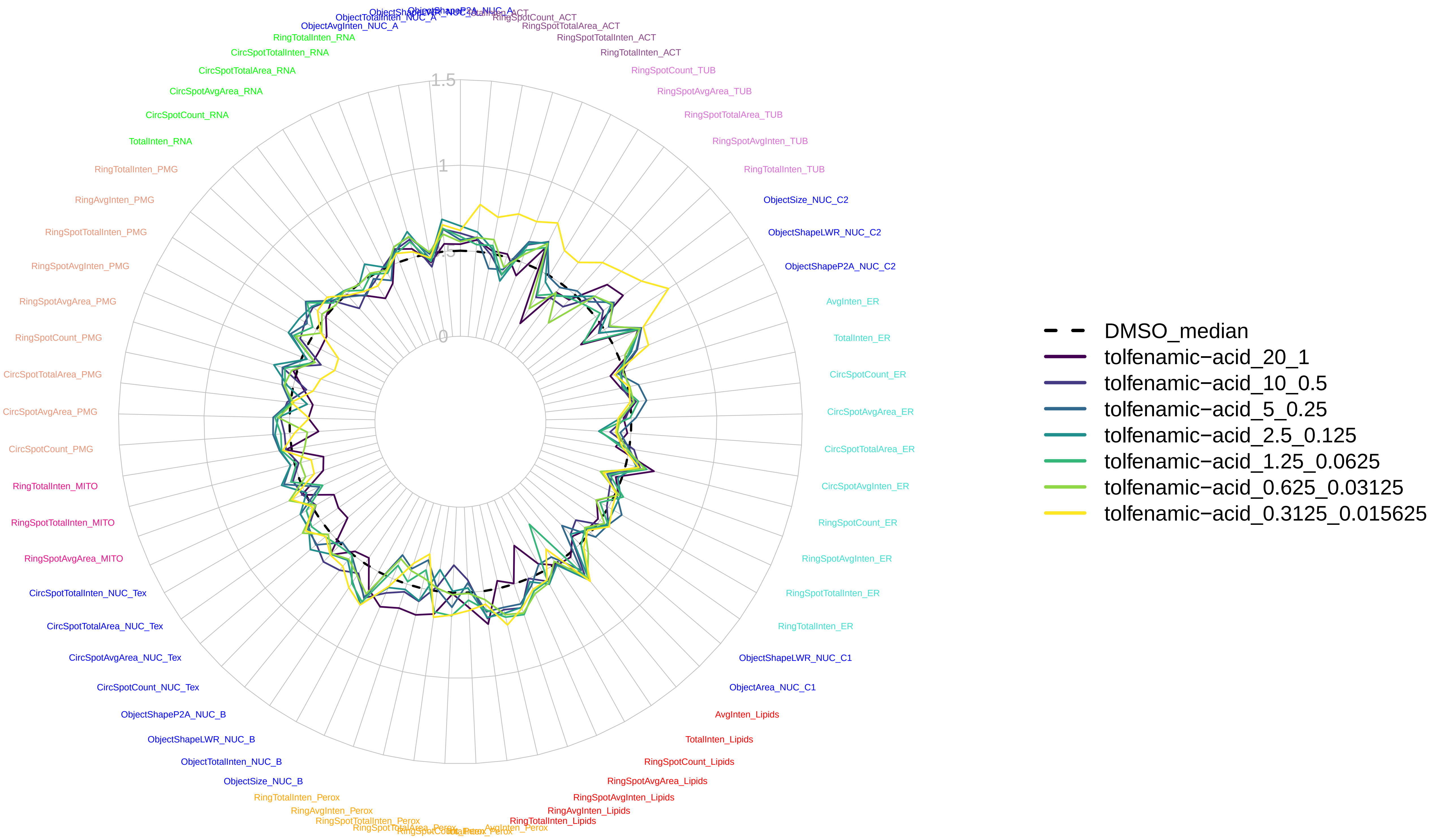

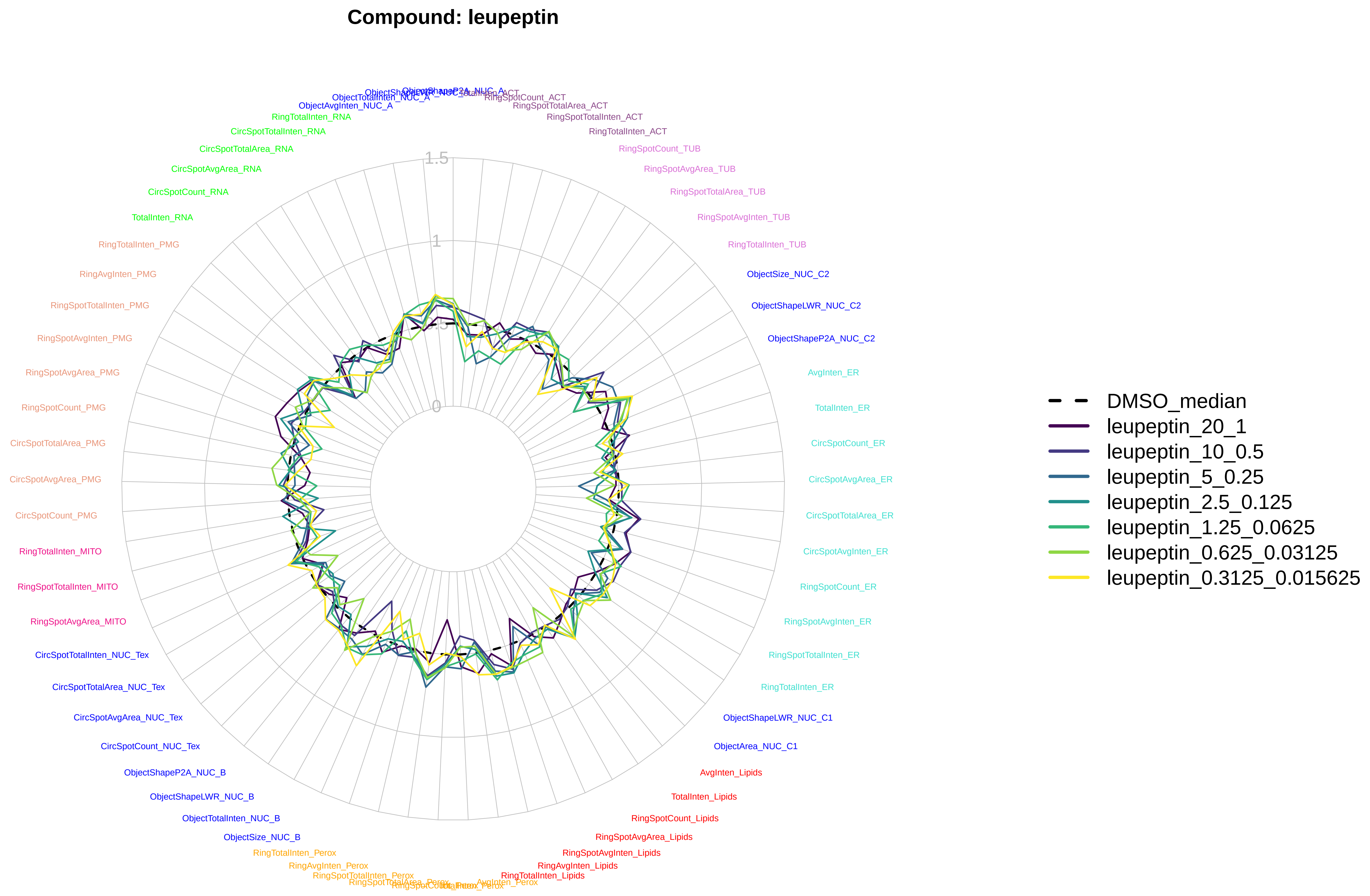
Compound: leupeptin
1.5
1
0.5
0
ObjectTotalInten_NUC_A
ObjectAvgInten_NUC_A
RingSpotCount_ACT
RingSpotTotalArea_ACT
RingSpotTotalInten_ACT
RingTotalInten_ACT
RingSpotCount_TUB
RingSpotAvgArea_TUB
RingSpotTotalArea_TUB
RingSpotAvgInten_TUB
RingTotalInten_TUB
ObjectSize_NUC_C2
ObjectShapeLWR_NUC_C2
ObjectShapeP2A_NUC_C2
AvgInten_ER
TotalInten_ER
CircSpotCount_ER
CircSpotAvgArea_ER
CircSpotTotalArea_ER
CircSpotAvgInten_ER
RingSpotCount_ER
RingSpotAvgInten_ER
RingSpotTotalInten_ER
RingTotalInten_ER
ObjectShapeLWR_NUC_C1
ObjectArea_NUC_C1
AvgInten_Lipids
TotalInten_Lipids
RingSpotCount_Lipids
RingSpotAvgArea_Lipids
RingSpotAvgInten_Lipids
RingAvgInten_Lipids
RingTotalInten_Lipids
AvgInten_Perox
RingSpotTotalInten_Perox
RingAvgInten_Perox
RingTotalInten_Perox
ObjectSize_NUC_B
ObjectTotalInten_NUC_B
ObjectShapeLWR_NUC_B
ObjectShapeP2A_NUC_B
CircSpotCount_NUC_Tex
CircSpotAvgArea_NUC_Tex
CircSpotTotalArea_NUC_Tex
CircSpotTotalInten_NUC_Tex
RingSpotAvgArea_MITO
RingSpotTotalInten_MITO
RingTotalInten_MITO
CircSpotCount_PMG
CircSpotAvgArea_PMG
CircSpotTotalArea_PMG
RingSpotCount_PMG
RingSpotAvgArea_PMG
RingSpotAvgInten_PMG
RingSpotTotalInten_PMG
RingAvgInten_PMG
RingTotalInten_PMG
TotalInten_RNA
CircSpotCount_RNA
CircSpotAvgArea_RNA
CircSpotTotalArea_RNA
CircSpotTotalInten_RNA
RingTotalInten_RNA
DMSO_median
leupeptin_20_1
leupeptin_10_0.5
leupeptin_5_0.25
leupeptin_2.5_0.125
leupeptin_1.25_0.0625
leupeptin_0.625_0.03125
leupeptin_0.3125_0.015625

Compound: amygdalin

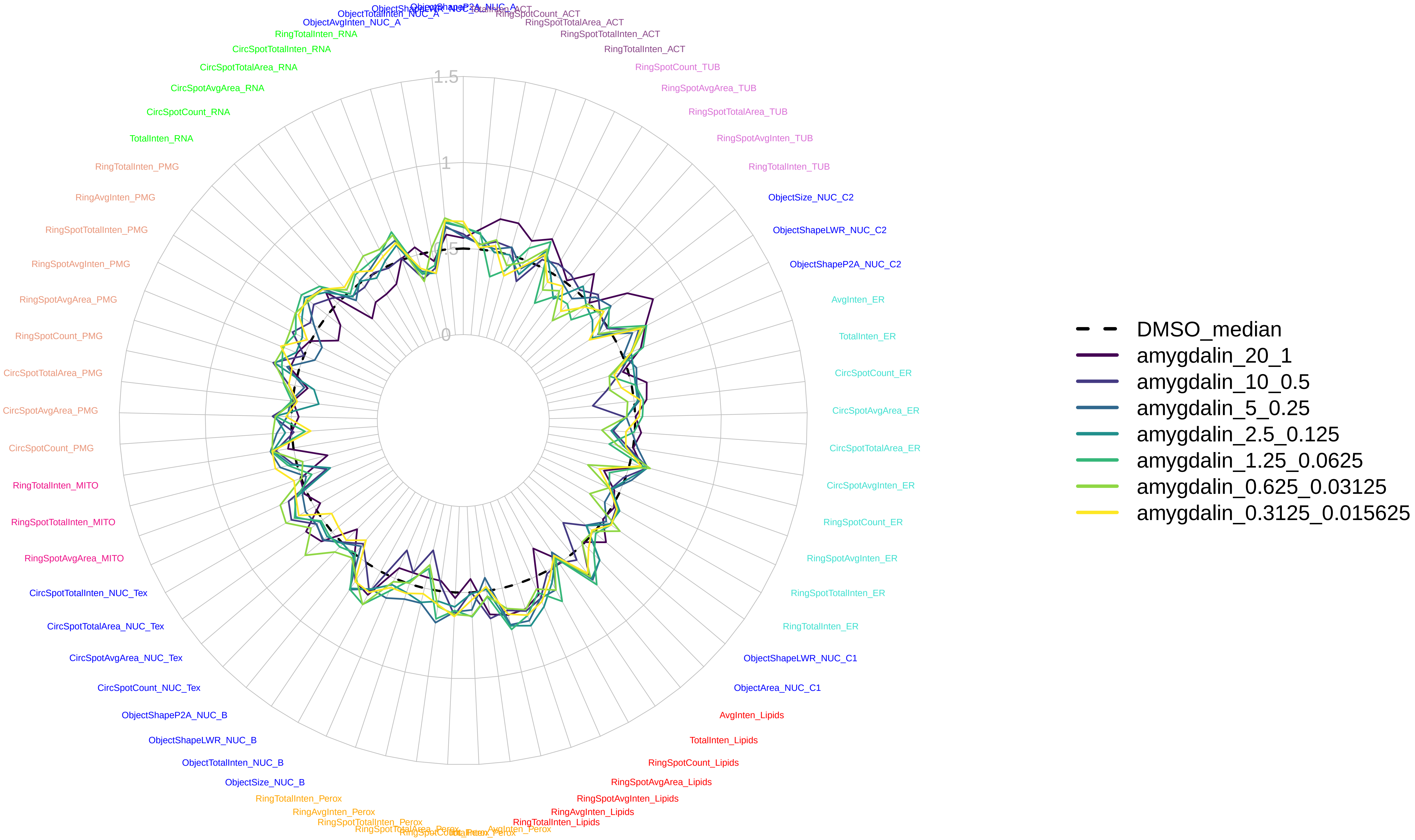

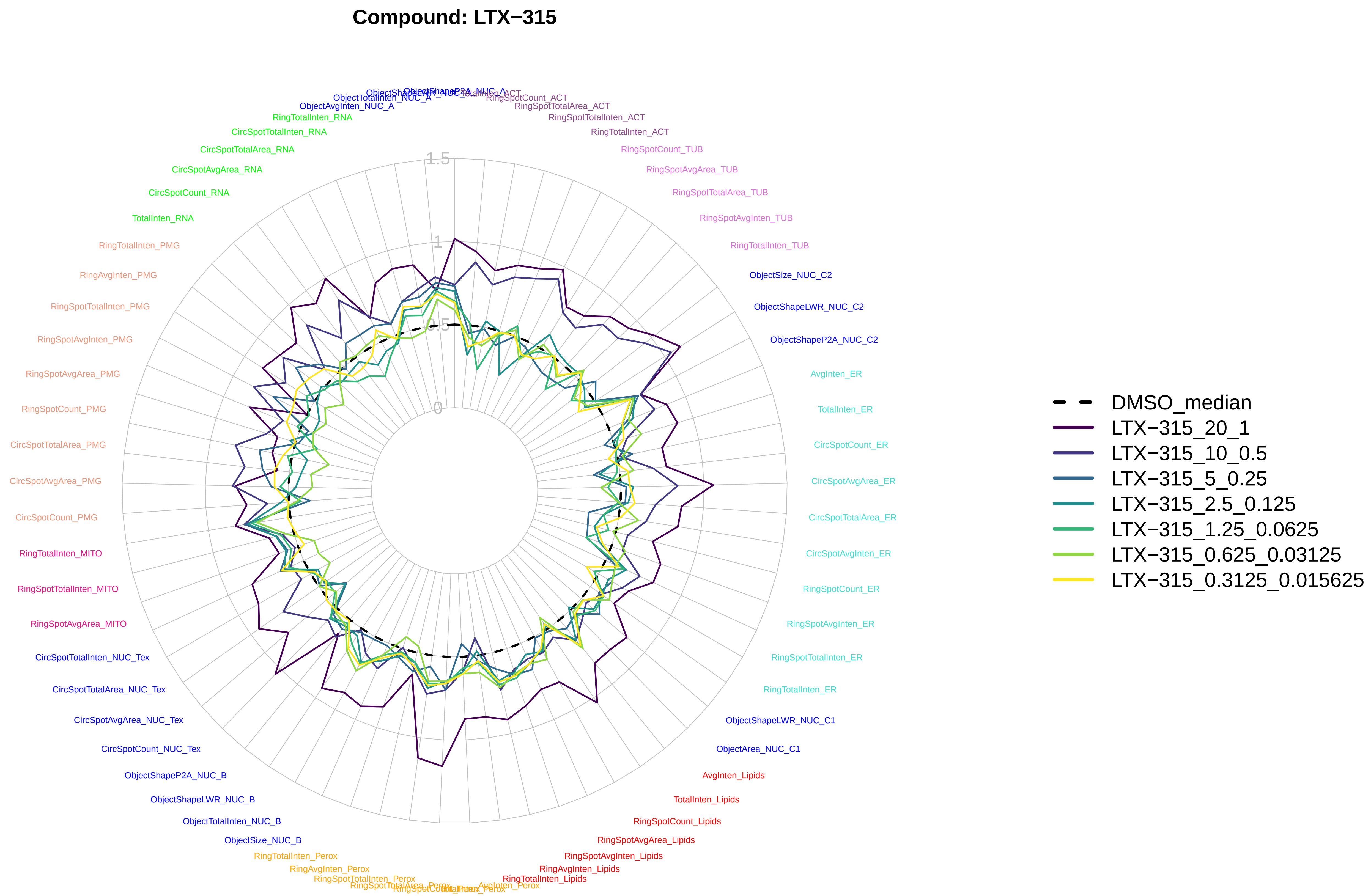

Compound: LTX−315
ObjectTotalInten_NUC_A
ObjectAvgInten_NUC_A
RingSpotCount_ACT
RingSpotTotalArea_ACT
RingSpotTotalInten_ACT
RingTotalInten_ACT
RingTotalInten_RNA
CircSpotTotalInten_RNA
CircSpotTotalArea_RNA
CircSpotAvgArea_RNA
CircSpotCount_RNA
TotalInten_RNA
RingTotalInten_PMG
RingAvgInten_PMG
RingSpotTotalInten_PMG
RingSpotAvgInten_PMG
RingSpotAvgArea_PMG
RingSpotCount_PMG
CircSpotTotalArea_PMG
CircSpotAvgArea_PMG
CircSpotCount_PMG
RingTotalInten_MITO
RingSpotTotalInten_MITO
RingSpotAvgArea_MITO
CircSpotTotalInten_NUC_Tex
CircSpotTotalArea_NUC_Tex
CircSpotAvgArea_NUC_Tex
CircSpotCount_NUC_Tex
ObjectShapeP2A_NUC_B
ObjectShapeLWR_NUC_B
ObjectTotalInten_NUC_B
ObjectSize_NUC_B
RingTotalInten_Perox
RingAvgInten_Perox
RingSpotTotalInten_Perox
AvgInten_Perox
RingSpotCount_TUB
RingSpotAvgArea_TUB
RingSpotTotalArea_TUB
RingSpotAvgInten_TUB
RingTotalInten_TUB
ObjectSize_NUC_C2
ObjectShapeLWR_NUC_C2
ObjectShapeP2A_NUC_C2
AvgInten_ER
TotalInten_ER
CircSpotCount_ER
CircSpotAvgArea_ER
CircSpotTotalArea_ER
CircSpotAvgInten_ER
RingSpotCount_ER
RingSpotAvgInten_ER
RingSpotTotalInten_ER
RingTotalInten_ER
ObjectShapeLWR_NUC_C1
ObjectArea_NUC_C1
AvgInten_Lipids
TotalInten_Lipids
RingSpotCount_Lipids
RingSpotAvgArea_Lipids
RingSpotAvgInten_Lipids
RingAvgInten_Lipids
RingTotalInten_Lipids
1.5
1
0.5
0
DMSO_median
LTX−315_20_1
LTX−315_10_0.5
LTX−315_5_0.25
LTX−315_2.5_0.125
LTX−315_1.25_0.0625
LTX−315_0.625_0.03125
LTX−315_0.3125_0.015625

Compound: erastin

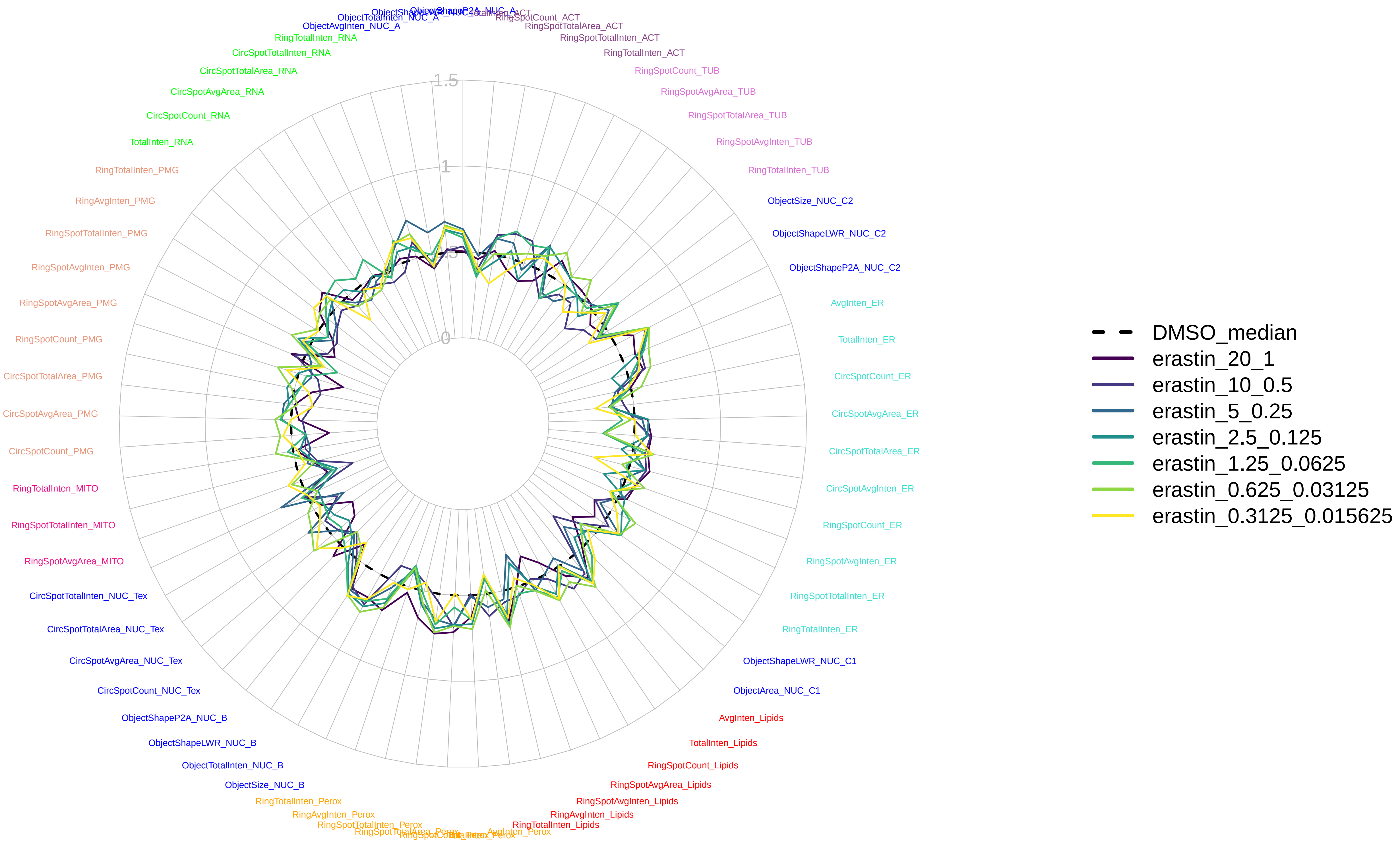

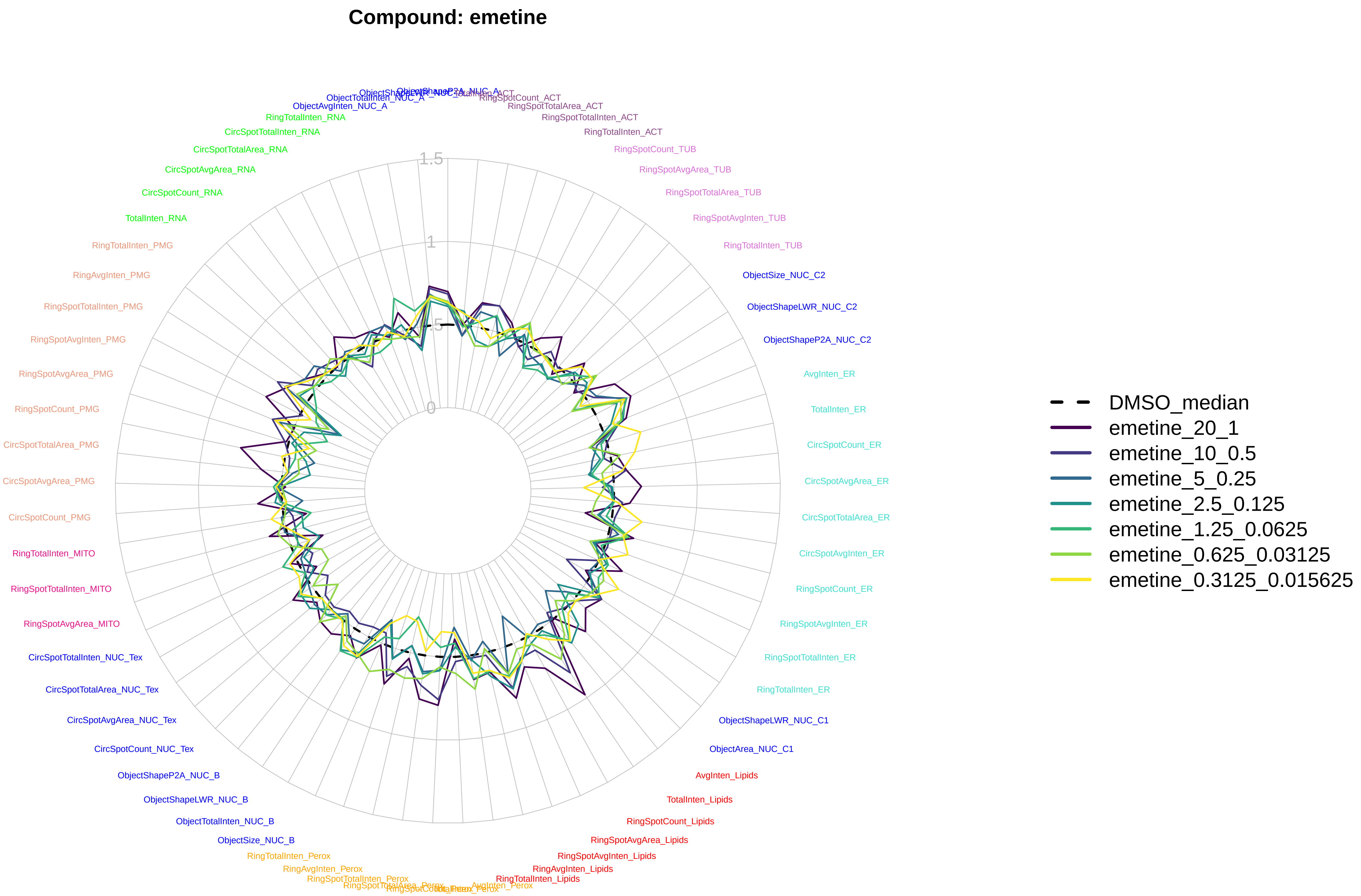
Compound: emetine
1.5
1
.5
0
RingSpotCount_ACT
RingSpotTotalArea_ACT
RingSpotTotalInten_ACT
RingTotalInten_ACT
RingSpotCount_TUB
RingSpotAvgArea_TUB
RingSpotTotalArea_TUB
RingSpotAvgInten_TUB
RingTotalInten_TUB
ObjectSize_NUC_C2
ObjectShapeLWR_NUC_C2
ObjectShapeP2A_NUC_C2
AvgInten_ER
TotalInten_ER
CircSpotCount_ER
CircSpotAvgArea_ER
CircSpotTotalArea_ER
CircSpotAvgInten_ER
RingSpotCount_ER
RingSpotAvgInten_ER
RingSpotTotalInten_ER
RingTotalInten_ER
ObjectShapeLWR_NUC_C1
ObjectArea_NUC_C1
AvgInten_Lipids
TotalInten_Lipids
RingSpotCount_Lipids
RingSpotAvgArea_Lipids
RingSpotAvgInten_Lipids
RingAvgInten_Lipids
RingTotalInten_Lipids
AvgInten_Perox
RingTotalInten_Perox
RingAvgInten_Perox
RingSpotTotalInten_Perox
ObjectSize_NUC_B
ObjectTotalInten_NUC_B
ObjectShapeLWR_NUC_B
ObjectShapeP2A_NUC_B
CircSpotCount_NUC_Tex
CircSpotAvgArea_NUC_Tex
CircSpotTotalArea_NUC_Tex
CircSpotTotalInten_NUC_Tex
RingSpotAvgArea_MITO
RingSpotTotalInten_MITO
RingTotalInten_MITO
CircSpotCount_PMG
CircSpotAvgArea_PMG
CircSpotTotalArea_PMG
RingSpotCount_PMG
RingSpotAvgArea_PMG
RingSpotAvgInten_PMG
RingSpotTotalInten_PMG
RingAvgInten_PMG
RingTotalInten_PMG
TotalInten_RNA
CircSpotCount_RNA
CircSpotAvgArea_RNA
CircSpotTotalArea_RNA
CircSpotTotalInten_RNA
RingTotalInten_RNA
ObjectAvgInten_NUC_A
ObjectTotalInten_NUC_A
DMSO_median
emetine_20_1
emetine_10_0.5
emetine_5_0.25
emetine_2.5_0.125
emetine_1.25_0.0625
emetine_0.625_0.03125
emetine_0.3125_0.015625

Compound: sirolimus

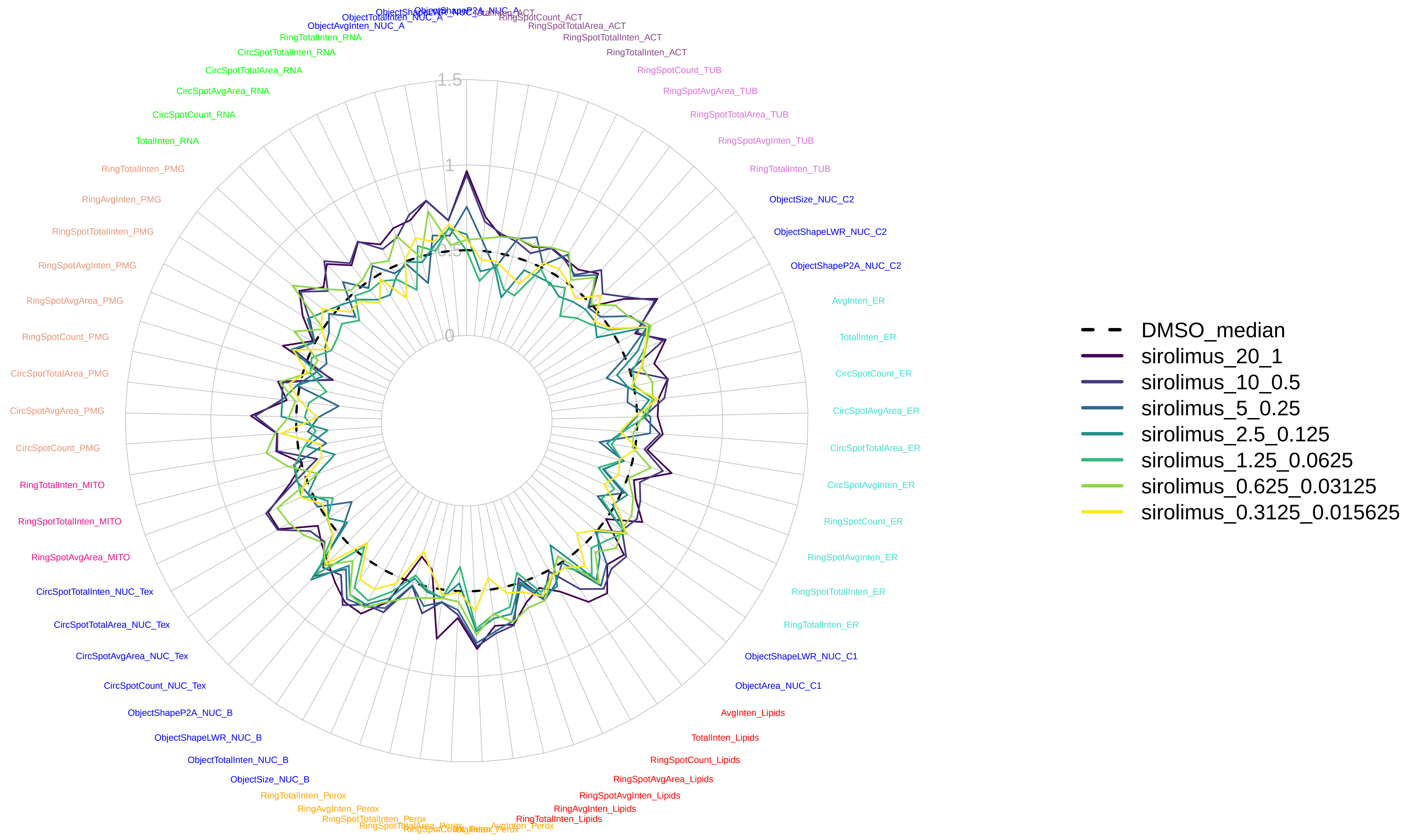

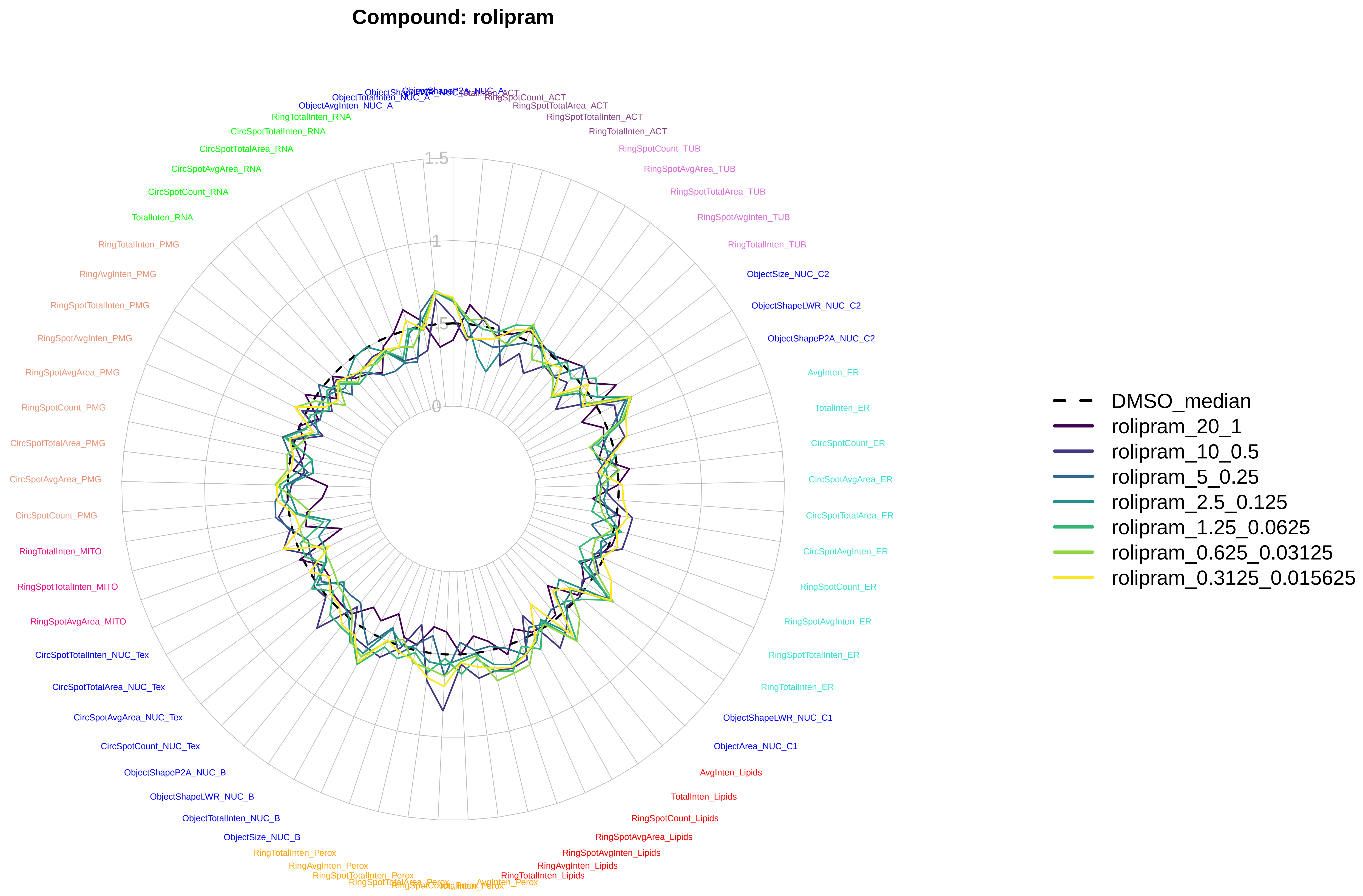
Compound: rolipram
ObjectShapeP2A_NUC_A
ObjectTotalInten_NUC_A
ObjectAvgInten_NUC_A
RingTotalInten_RNA
CircSpotTotalInten_RNA
CircSpotTotalArea_RNA
CircSpotAvgArea_RNA
CircSpotCount_RNA
TotalInten_RNA
RingTotalInten_PMG
RingAvgInten_PMG
RingSpotTotalInten_PMG
RingSpotAvgInten_PMG
RingSpotAvgArea_PMG
RingSpotCount_PMG
CircSpotTotalArea_PMG
CircSpotAvgArea_PMG
CircSpotCount_PMG
RingTotalInten_MITO
RingSpotTotalInten_MITO
RingSpotAvgArea_MITO
CircSpotTotalInten_NUC_Tex
CircSpotTotalArea_NUC_Tex
CircSpotAvgArea_NUC_Tex
CircSpotCount_NUC_Tex
ObjectShapeP2A_NUC_B
ObjectShapeLWR_NUC_B
ObjectTotalInten_NUC_B
ObjectSize_NUC_B
RingTotalInten_Perox
RingAvgInten_Perox
RingSpotTotalInten_Perox
AvgInten_Perox
RingTotalInten_Lipids
RingAvgInten_Lipids
RingSpotAvgInten_Lipids
RingSpotAvgArea_Lipids
RingSpotCount_Lipids
TotalInten_Lipids
AvgInten_Lipids
ObjectArea_NUC_C1
ObjectShapeLWR_NUC_C1
RingTotalInten_ER
RingSpotTotalInten_ER
RingSpotAvgInten_ER
RingSpotCount_ER
CircSpotAvgInten_ER
CircSpotTotalArea_ER
CircSpotAvgArea_ER
CircSpotCount_ER
TotalInten_ER
AvgInten_ER
ObjectShapeP2A_NUC_C2
ObjectShapeLWR_NUC_C2
ObjectSize_NUC_C2
RingTotalInten_TUB
RingSpotAvgInten_TUB
RingSpotTotalArea_TUB
RingSpotAvgArea_TUB
RingSpotCount_TUB
RingTotalInten_ACT
RingSpotTotalInten_ACT
RingSpotTotalArea_ACT
RingSpotCount_ACT
1.5
1
.5
0
DMSO_median
rolipram_20_1
rolipram_10_0.5
rolipram_5_0.25
rolipram_2.5_0.125
rolipram_1.25_0.0625
rolipram_0.625_0.03125
rolipram_0.3125_0.015625

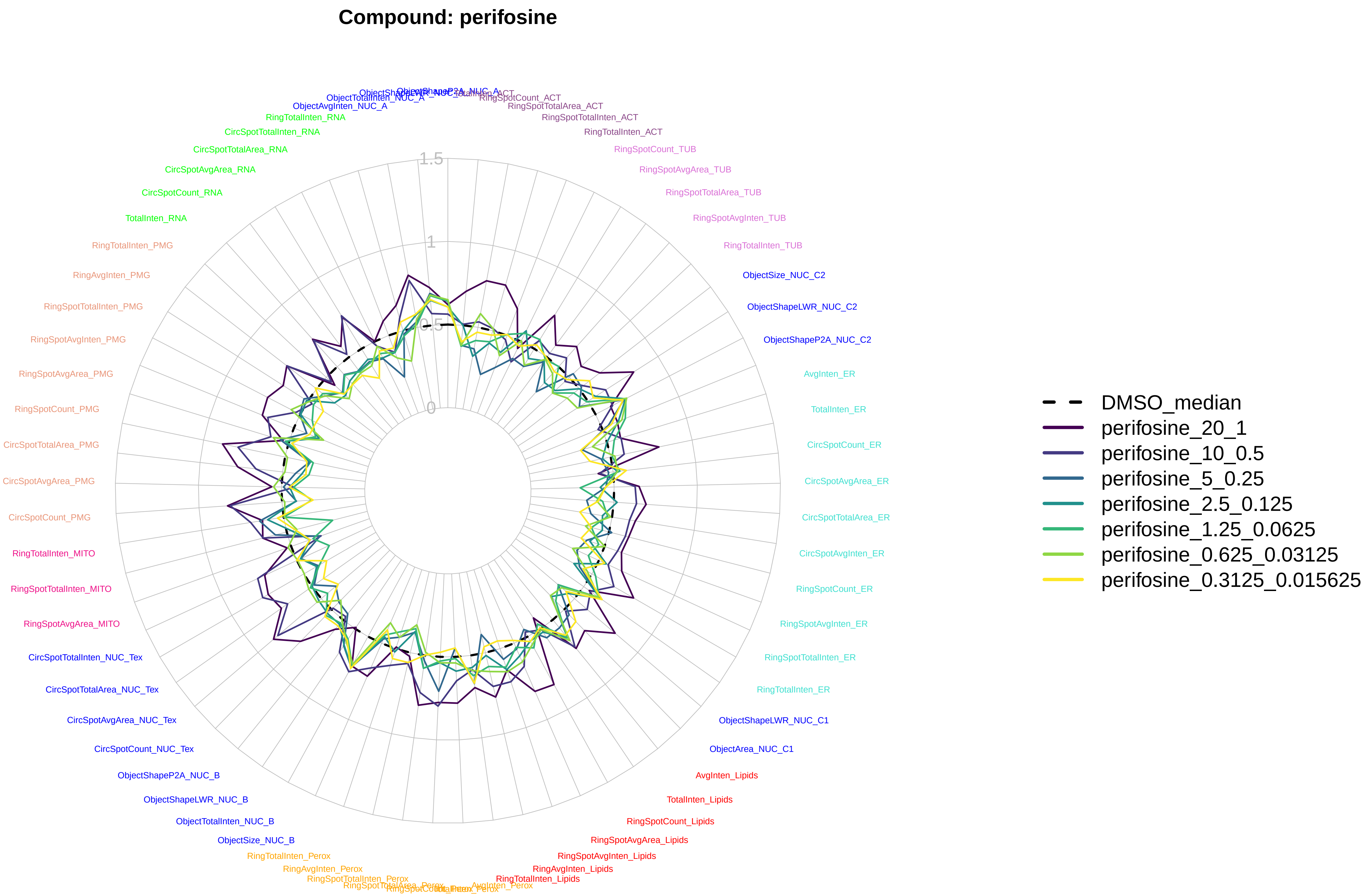

Compound: perifosine
1.5
1
0.5
0
ObjectTotalInten_NUC_A
ObjectAvgInten_NUC_A
RingTotalInten_RNA
CircSpotTotalInten_RNA
CircSpotTotalArea_RNA
CircSpotAvgArea_RNA
CircSpotCount_RNA
TotalInten_RNA
RingTotalInten_PMG
RingAvgInten_PMG
RingSpotTotalInten_PMG
RingSpotAvgInten_PMG
RingSpotAvgArea_PMG
RingSpotCount_PMG
CircSpotTotalArea_PMG
CircSpotAvgArea_PMG
CircSpotCount_PMG
RingTotalInten_MITO
RingSpotTotalInten_MITO
RingSpotAvgArea_MITO
CircSpotTotalInten_NUC_Tex
CircSpotTotalArea_NUC_Tex
CircSpotAvgArea_NUC_Tex
CircSpotCount_NUC_Tex
ObjectShapeP2A_NUC_B
ObjectShapeLWR_NUC_B
ObjectTotalInten_NUC_B
ObjectSize_NUC_B
RingTotalInten_Perox
RingAvgInten_Perox
RingSpotTotalInten_Perox
AvgInten_Perox
RingTotalInten_Lipids
RingAvgInten_Lipids
RingSpotAvgInten_Lipids
RingSpotAvgArea_Lipids
RingSpotCount_Lipids
TotalInten_Lipids
AvgInten_Lipids
ObjectArea_NUC_C1
ObjectShapeLWR_NUC_C1
RingTotalInten_ER
RingSpotTotalInten_ER
RingSpotAvgInten_ER
RingSpotCount_ER
CircSpotAvgInten_ER
CircSpotTotalArea_ER
CircSpotAvgArea_ER
CircSpotCount_ER
TotalInten_ER
AvgInten_ER
ObjectShapeP2A_NUC_C2
ObjectShapeLWR_NUC_C2
ObjectSize_NUC_C2
RingTotalInten_TUB
RingSpotAvgInten_TUB
RingSpotTotalArea_TUB
RingSpotAvgArea_TUB
RingSpotCount_TUB
RingTotalInten_ACT
RingSpotTotalInten_ACT
RingSpotTotalArea_ACT
RingSpotCount_ACT
DMSO_median
perifosine_20_1
perifosine_10_0.5
perifosine_5_0.25
perifosine_2.5_0.125
perifosine_1.25_0.0625
perifosine_0.625_0.03125
perifosine_0.3125_0.015625

Compound: golgicide−A

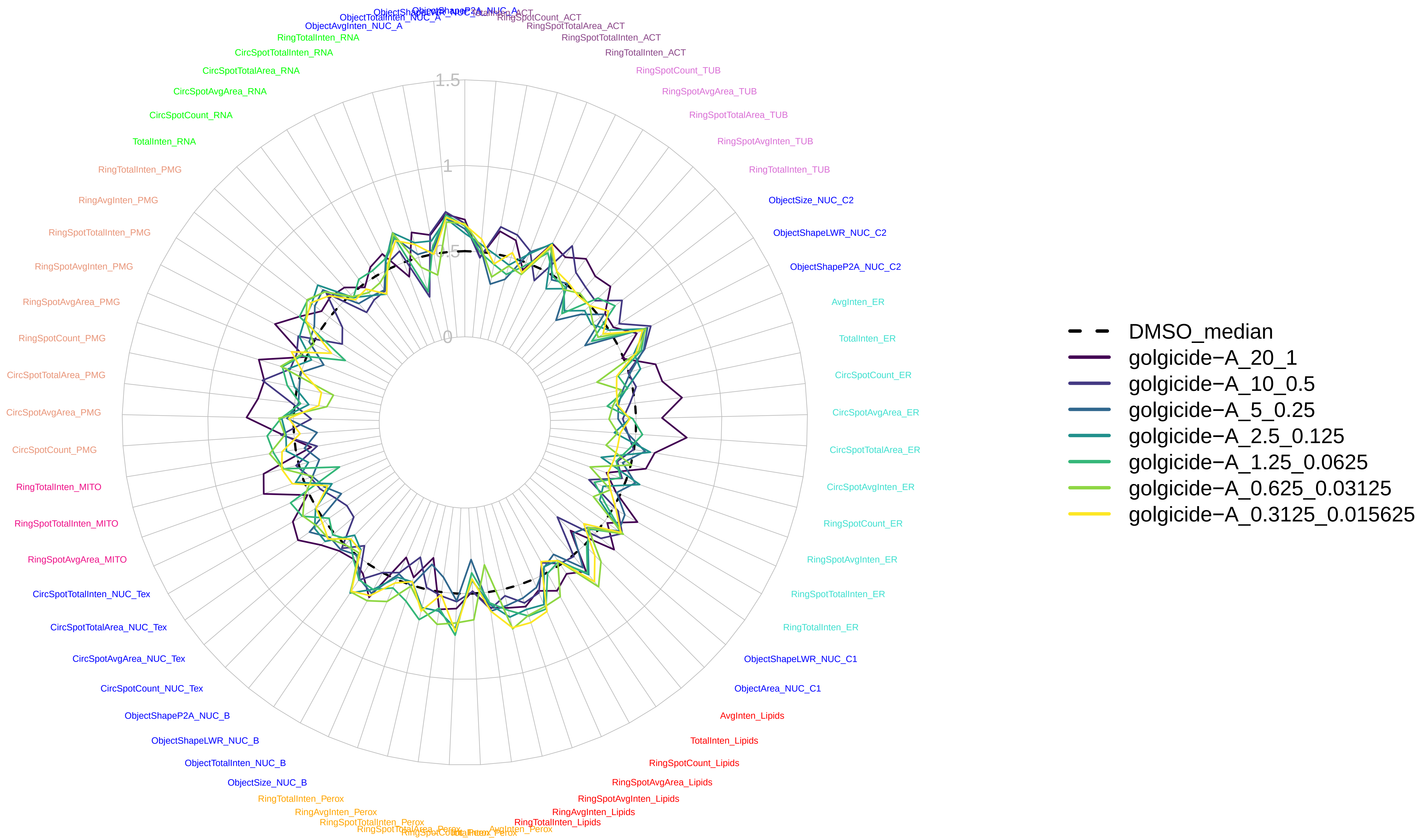

Compound: puromycin

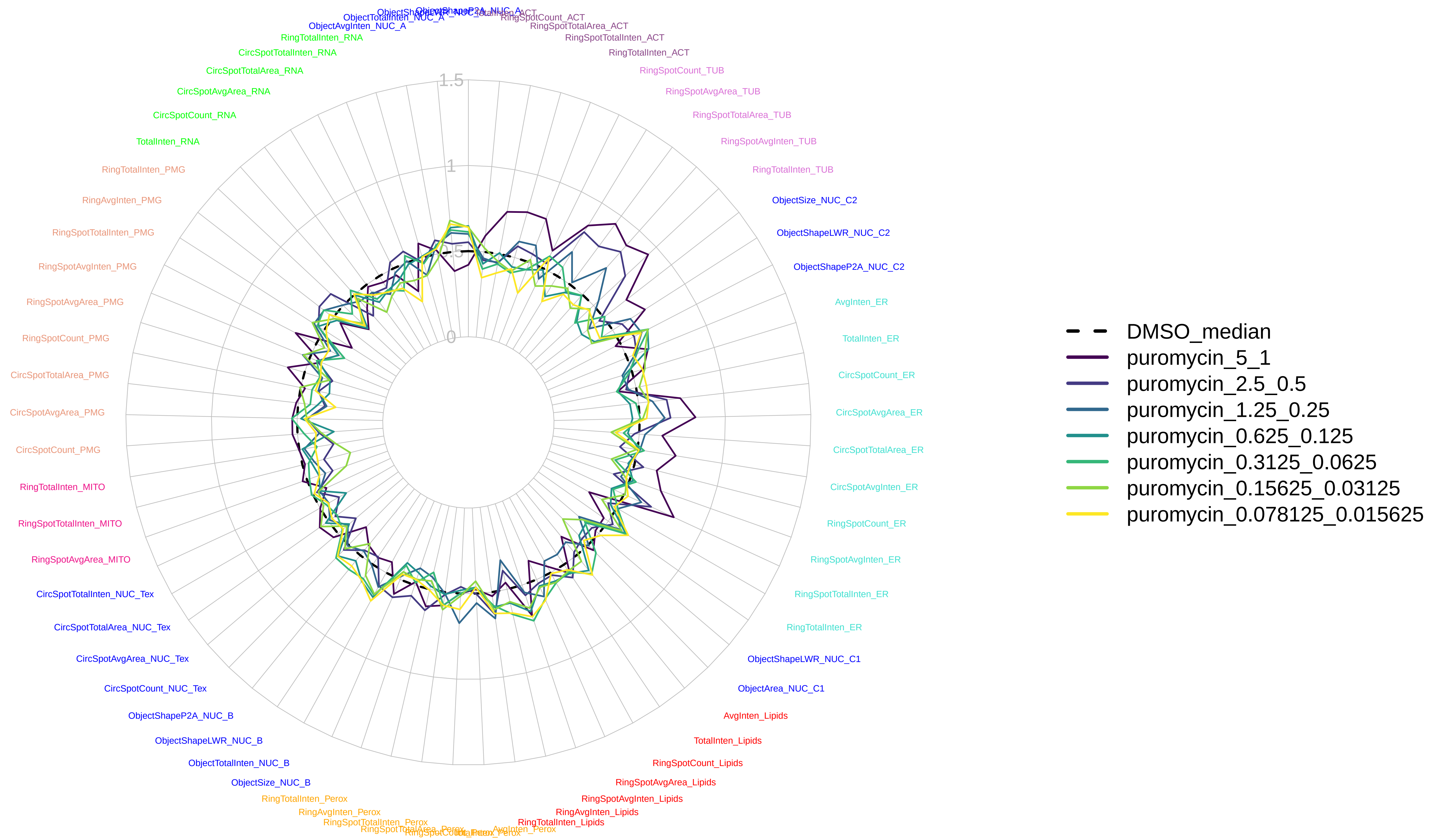

Compound: CK−636

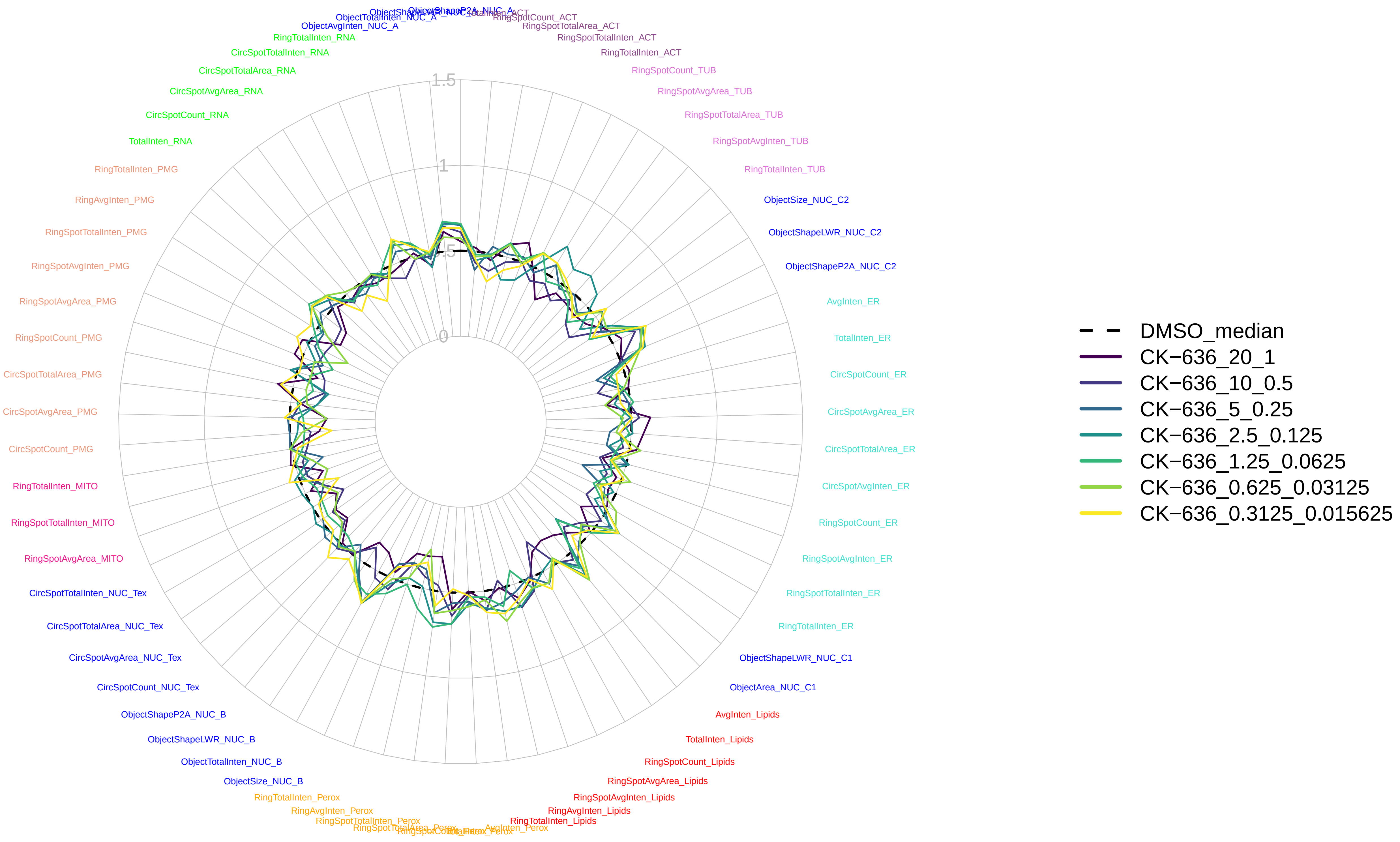

Compound: nocodazole

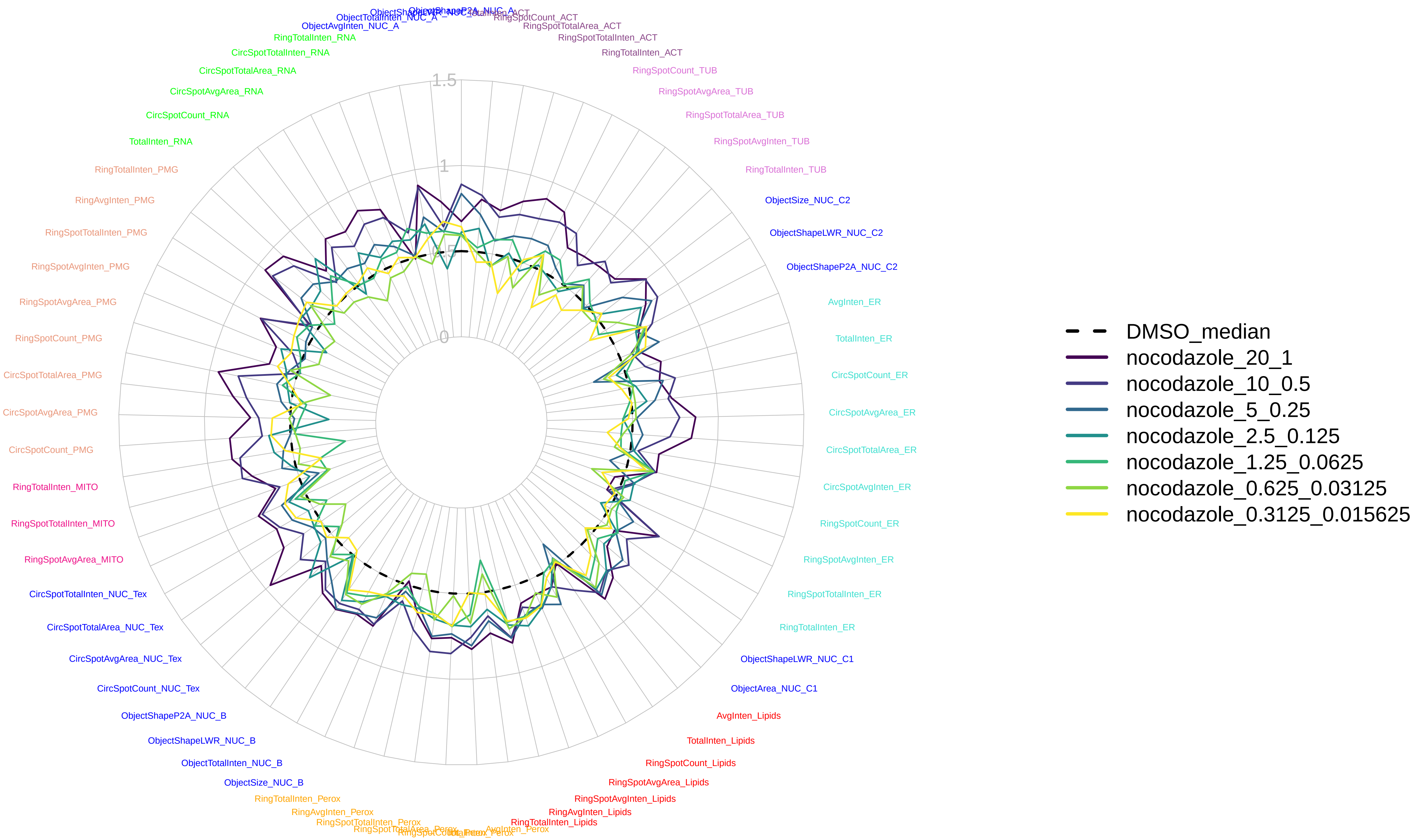

Compound: paclitaxel

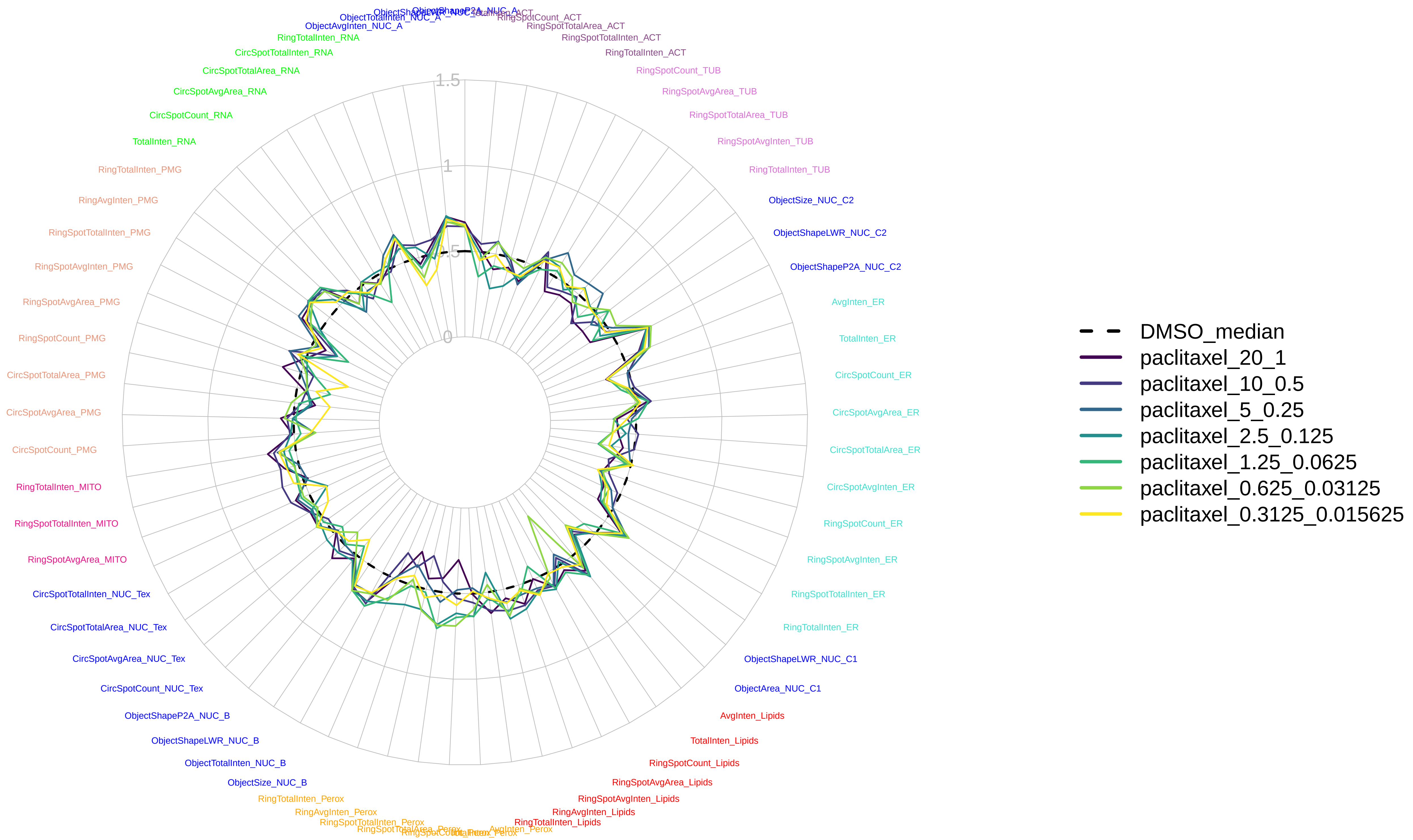

Compound: NSC−697923

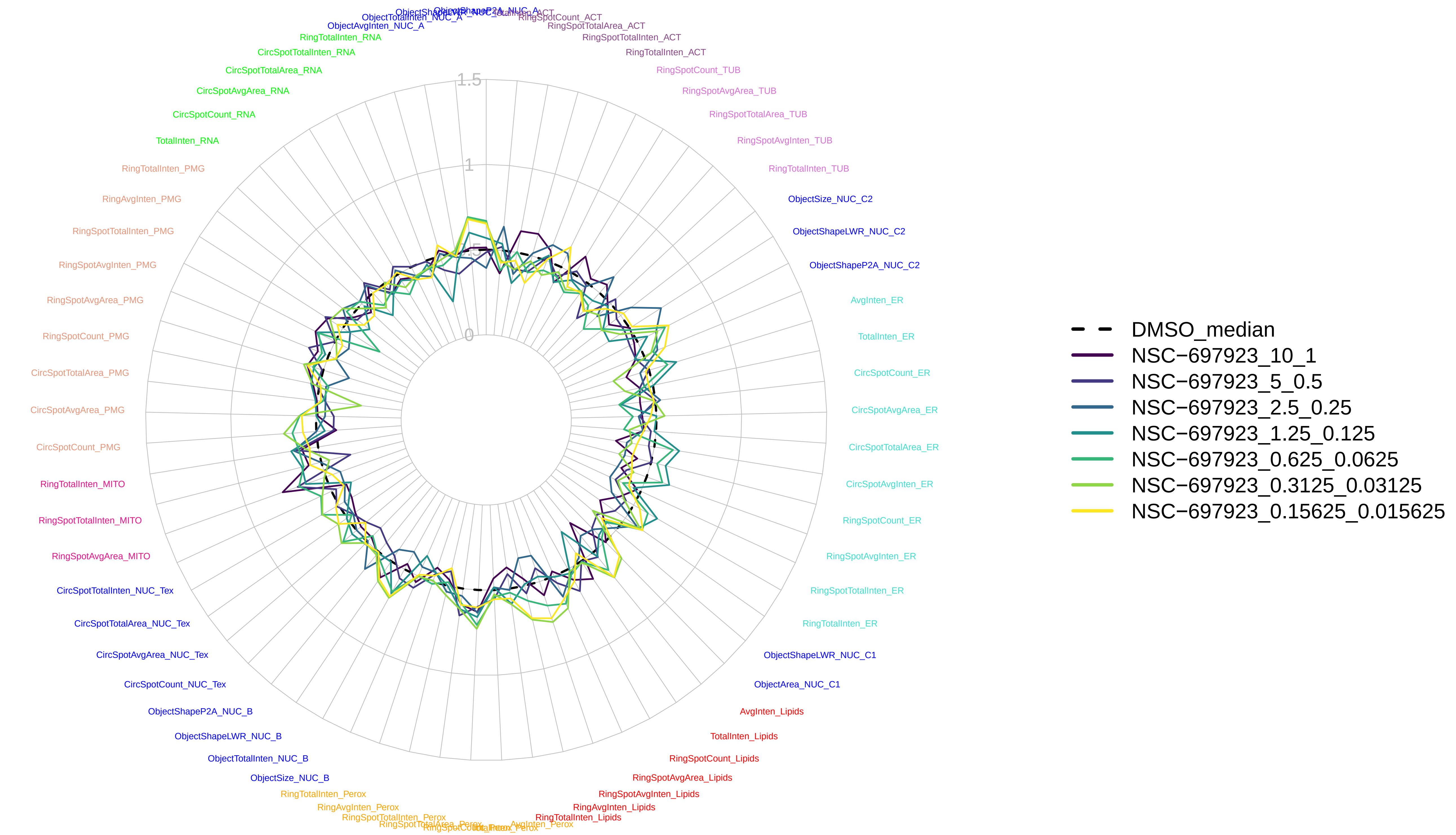

Compound: antimycin−a

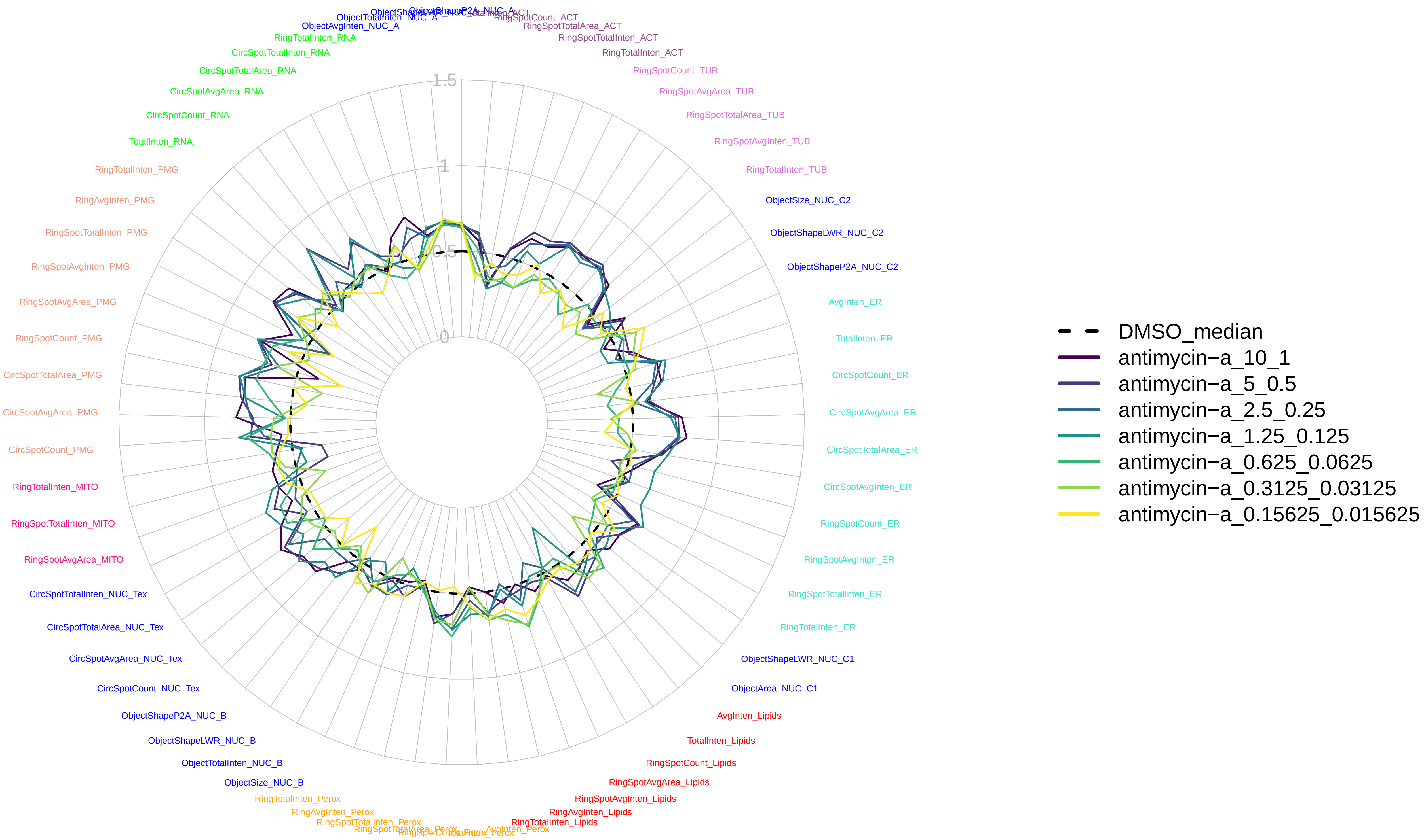

Compound: oligomycin−a

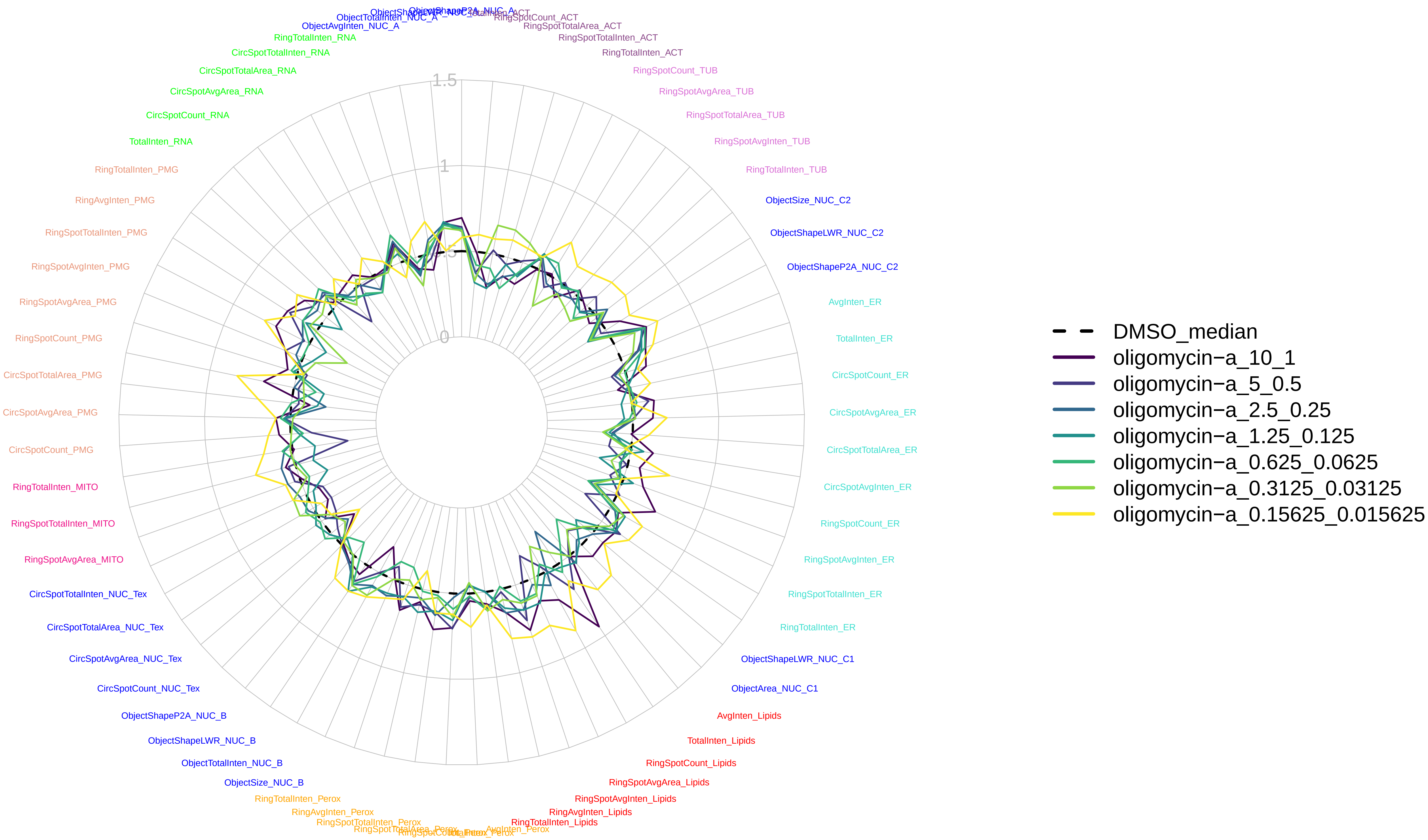

Compound: mdivi−1

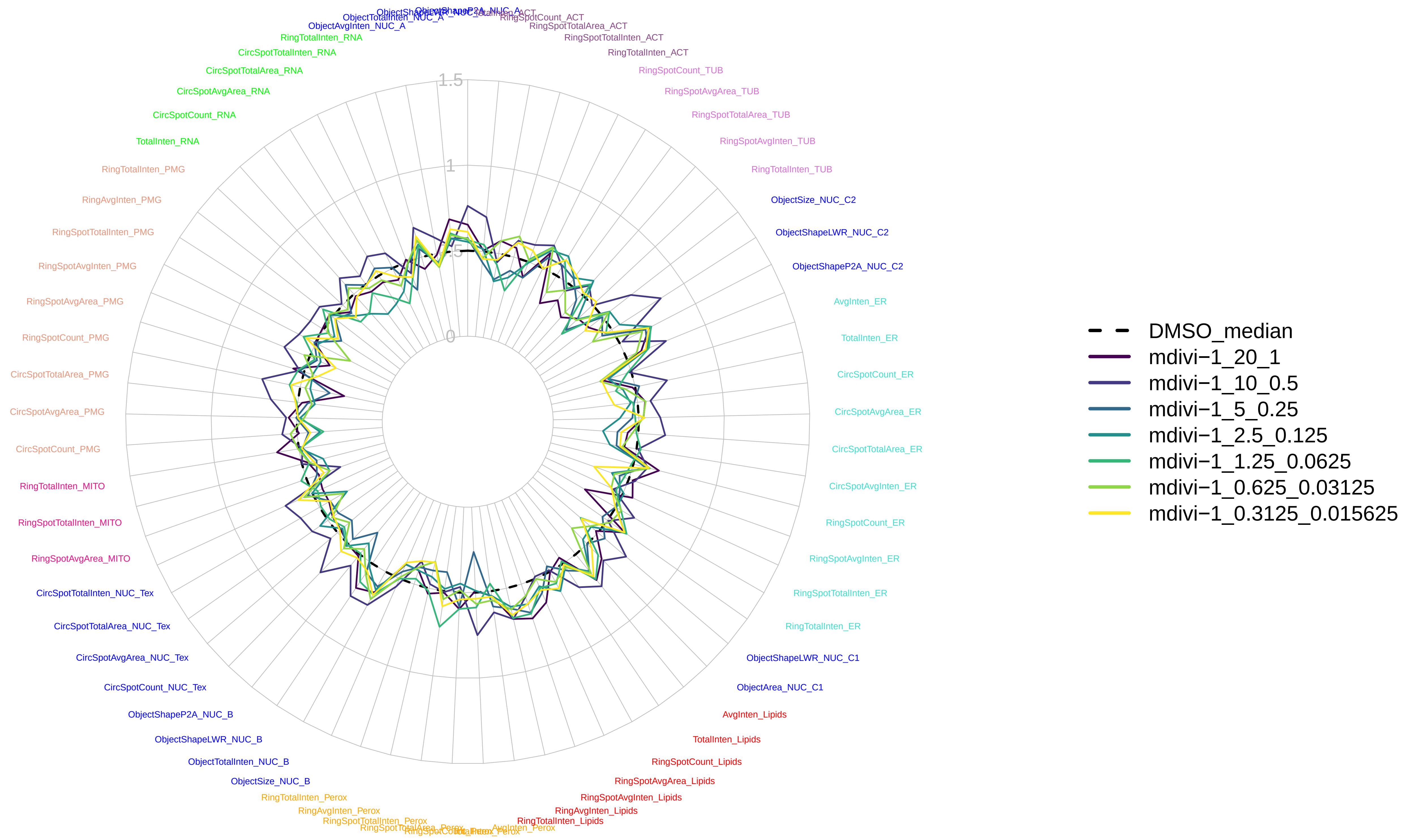

Compound: amsacrine

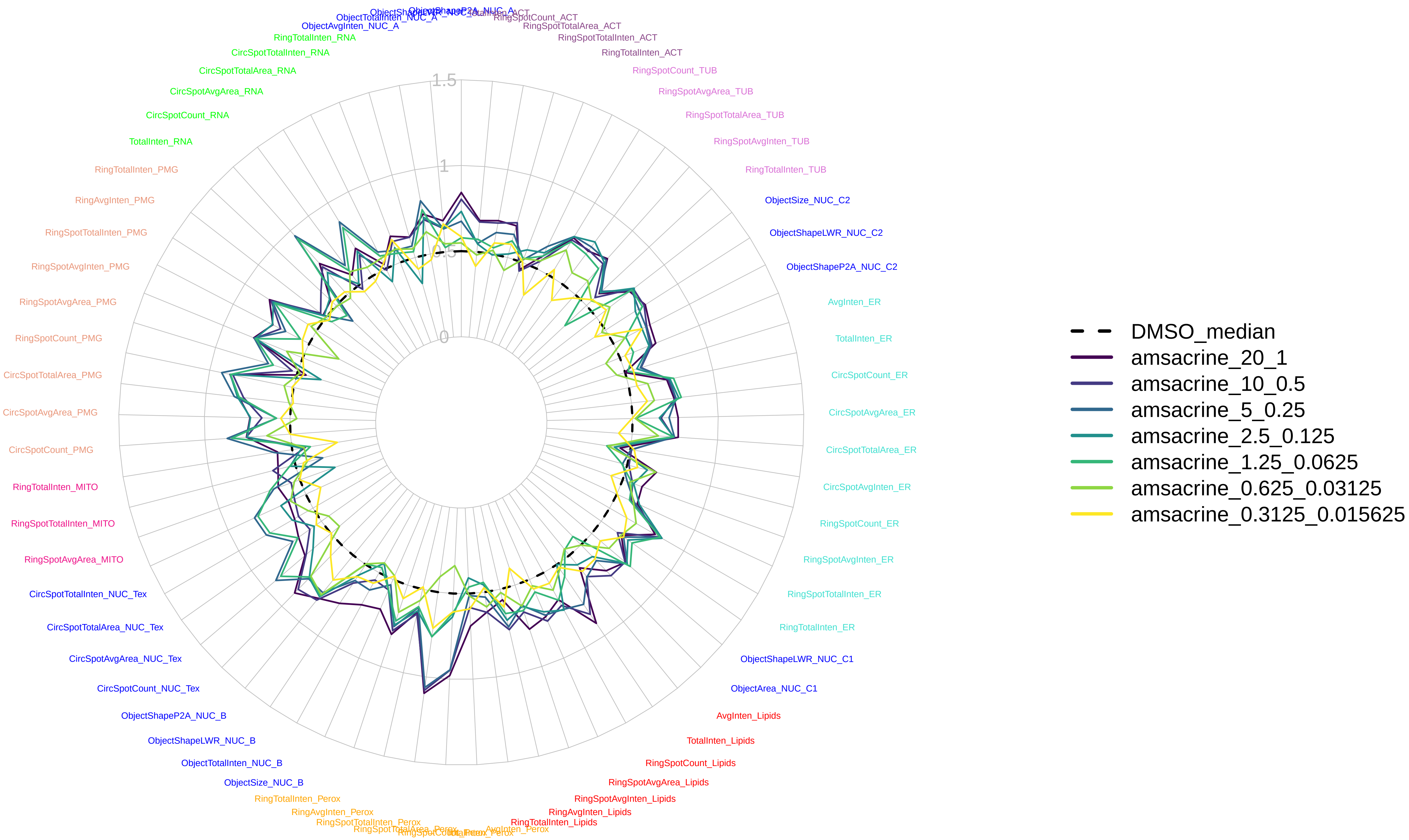

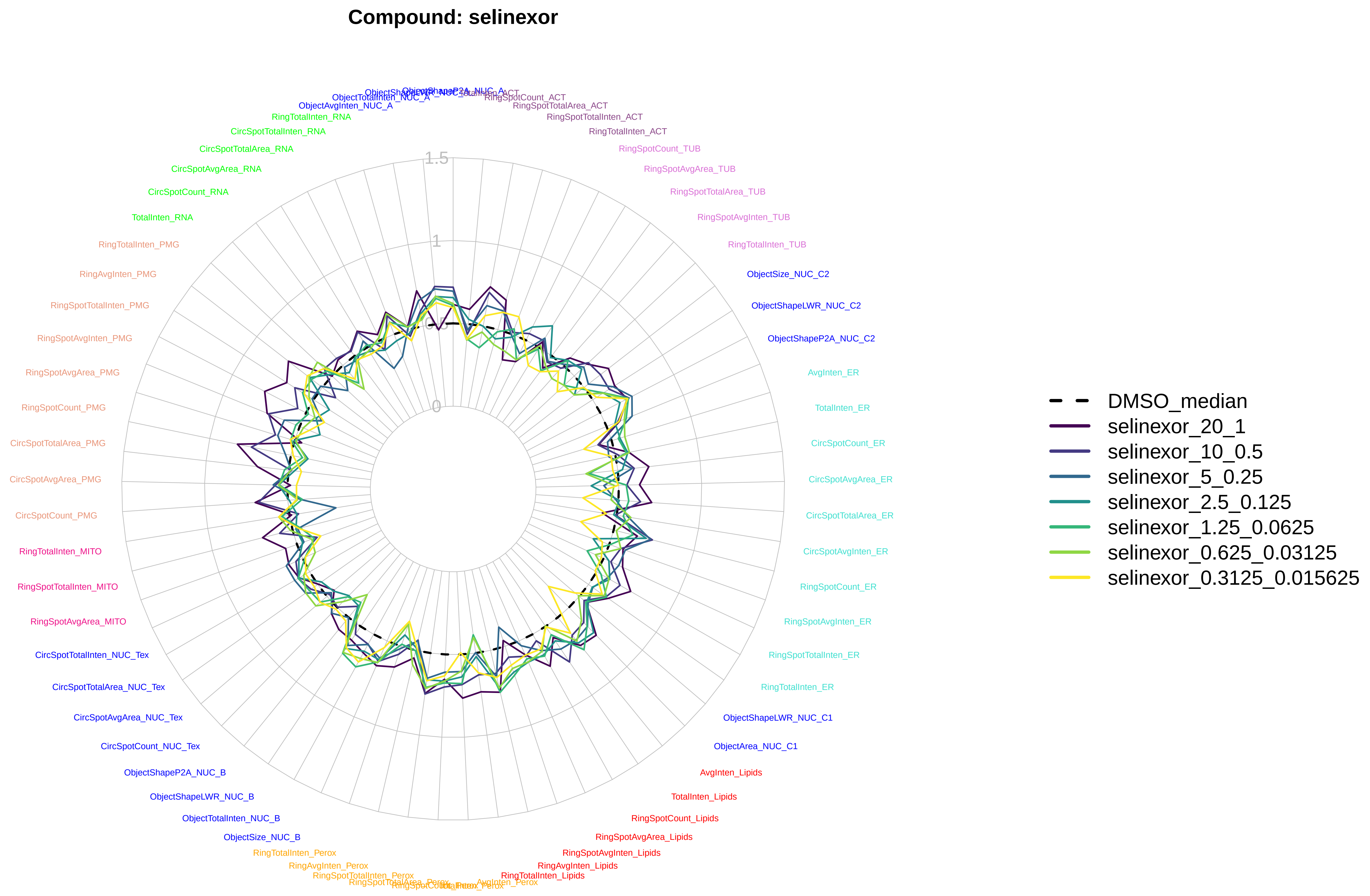
Compound: selinexor
ObjectShapeP2A_NUC_A
ObjectTotalInten_NUC_A
ObjectAvgInten_NUC_A
RingSpotCount_ACT
RingSpotTotalArea_ACT
RingSpotTotalInten_ACT
RingTotalInten_ACT
RingSpotCount_TUB
RingSpotAvgArea_TUB
RingSpotTotalArea_TUB
RingSpotAvgInten_TUB
RingTotalInten_TUB
ObjectSize_NUC_C2
ObjectShapeLWR_NUC_C2
ObjectShapeP2A_NUC_C2
AvgInten_ER
TotalInten_ER
CircSpotCount_ER
CircSpotAvgArea_ER
CircSpotTotalArea_ER
CircSpotAvgInten_ER
RingSpotCount_ER
RingSpotAvgInten_ER
RingSpotTotalInten_ER
RingTotalInten_ER
ObjectShapeLWR_NUC_C1
ObjectArea_NUC_C1
AvgInten_Lipids
TotalInten_Lipids
RingSpotCount_Lipids
RingSpotAvgArea_Lipids
RingSpotAvgInten_Lipids
RingAvgInten_Lipids
RingTotalInten_Lipids
AvgInten_Perox
RingSpotTotalArea_Perox
RingSpotTotalInten_Perox
RingAvgInten_Perox
RingTotalInten_Perox
ObjectSize_NUC_B
ObjectTotalInten_NUC_B
ObjectShapeLWR_NUC_B
ObjectShapeP2A_NUC_B
CircSpotCount_NUC_Tex
CircSpotAvgArea_NUC_Tex
CircSpotTotalArea_NUC_Tex
CircSpotTotalInten_NUC_Tex
RingSpotAvgArea_MITO
RingSpotTotalInten_MITO
RingTotalInten_MITO
CircSpotCount_PMG
CircSpotAvgArea_PMG
CircSpotTotalArea_PMG
RingSpotCount_PMG
RingSpotAvgArea_PMG
RingSpotAvgInten_PMG
RingSpotTotalInten_PMG
RingAvgInten_PMG
RingTotalInten_PMG
TotalInten_RNA
CircSpotCount_RNA
CircSpotAvgArea_RNA
CircSpotTotalArea_RNA
CircSpotTotalInten_RNA
RingTotalInten_RNA
1.5
1
0.5
0
DMSO_median
selinexor_20_1
selinexor_10_0.5
selinexor_5_0.25
selinexor_2.5_0.125
selinexor_1.25_0.0625
selinexor_0.625_0.03125
selinexor_0.3125_0.015625

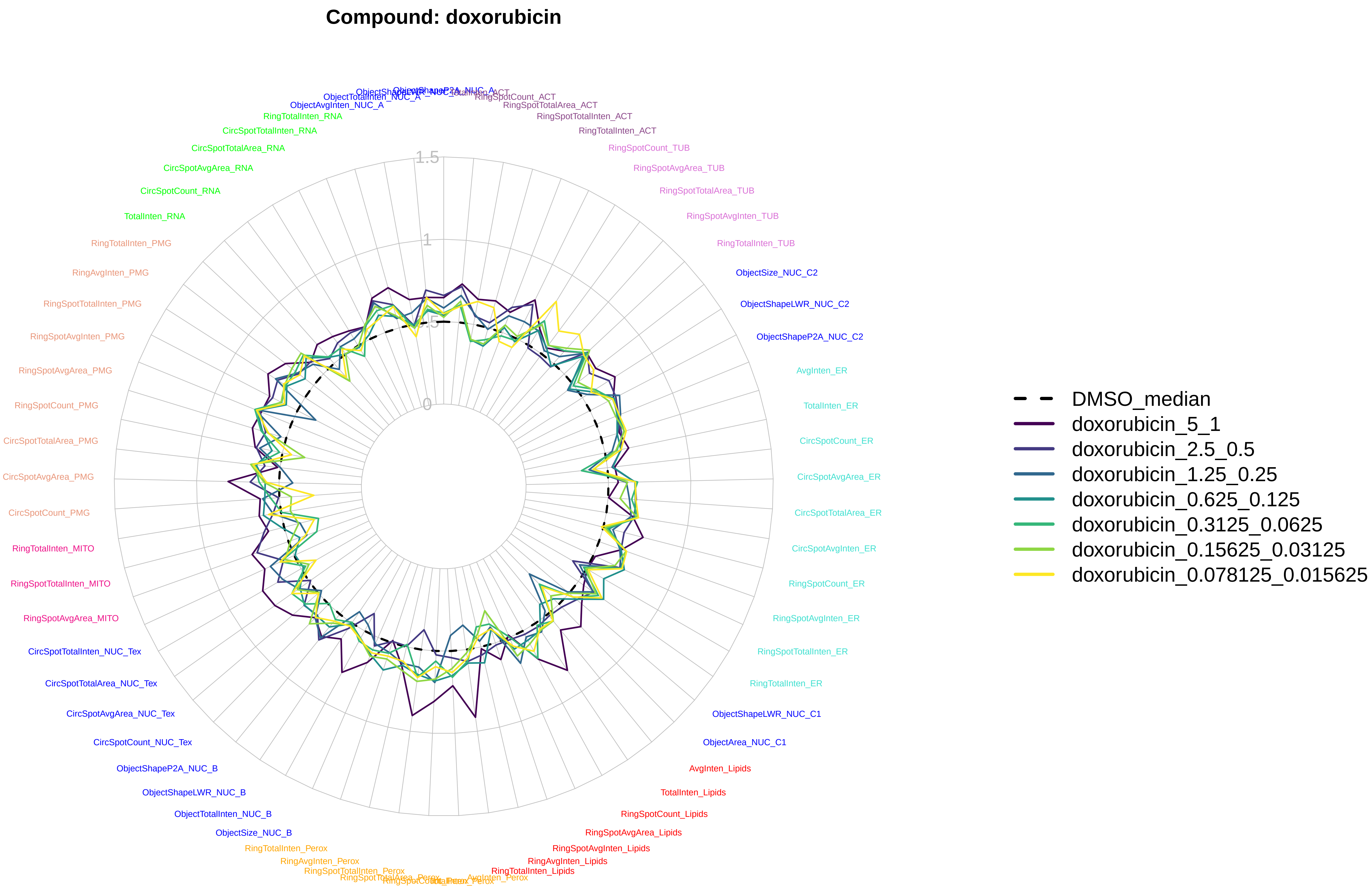
Compound: doxorubicin
ObjectShapeP2A_NUC_A
ObjectTotalInten_NUC_A
ObjectAvgInten_NUC_A
RingSpotCount_ACT
RingSpotTotalArea_ACT
RingSpotTotalInten_ACT
RingTotalInten_ACT
RingSpotCount_TUB
RingSpotAvgArea_TUB
RingSpotTotalArea_TUB
RingSpotAvgInten_TUB
RingTotalInten_TUB
ObjectSize_NUC_C2
ObjectShapeLWR_NUC_C2
ObjectShapeP2A_NUC_C2
AvgInten_ER
TotalInten_ER
CircSpotCount_ER
CircSpotAvgArea_ER
CircSpotTotalArea_ER
CircSpotAvgInten_ER
RingSpotCount_ER
RingSpotAvgInten_ER
RingSpotTotalInten_ER
RingTotalInten_ER
ObjectShapeLWR_NUC_C1
ObjectArea_NUC_C1
AvgInten_Lipids
TotalInten_Lipids
RingSpotCount_Lipids
RingSpotAvgArea_Lipids
RingSpotAvgInten_Lipids
RingAvgInten_Lipids
RingTotalInten_Lipids
AvgInten_Perox
RingTotalInten_Perox
RingAvgInten_Perox
RingSpotTotalInten_Perox
ObjectSize_NUC_B
ObjectTotalInten_NUC_B
ObjectShapeLWR_NUC_B
ObjectShapeP2A_NUC_B
CircSpotCount_NUC_Tex
CircSpotAvgArea_NUC_Tex
CircSpotTotalArea_NUC_Tex
CircSpotTotalInten_NUC_Tex
RingSpotAvgArea_MITO
RingSpotTotalInten_MITO
RingTotalInten_MITO
CircSpotCount_PMG
CircSpotAvgArea_PMG
CircSpotTotalArea_PMG
RingSpotCount_PMG
RingSpotAvgArea_PMG
RingSpotAvgInten_PMG
RingSpotTotalInten_PMG
RingAvgInten_PMG
RingTotalInten_PMG
TotalInten_RNA
CircSpotCount_RNA
CircSpotAvgArea_RNA
CircSpotTotalArea_RNA
CircSpotTotalInten_RNA
RingTotalInten_RNA
1.5
1
0
DMSO_median
doxorubicin_5_1
doxorubicin_2.5_0.5
doxorubicin_1.25_0.25
doxorubicin_0.625_0.125
doxorubicin_0.3125_0.0625
doxorubicin_0.15625_0.03125
doxorubicin_0.078125_0.015625

Compound: gemcitabine

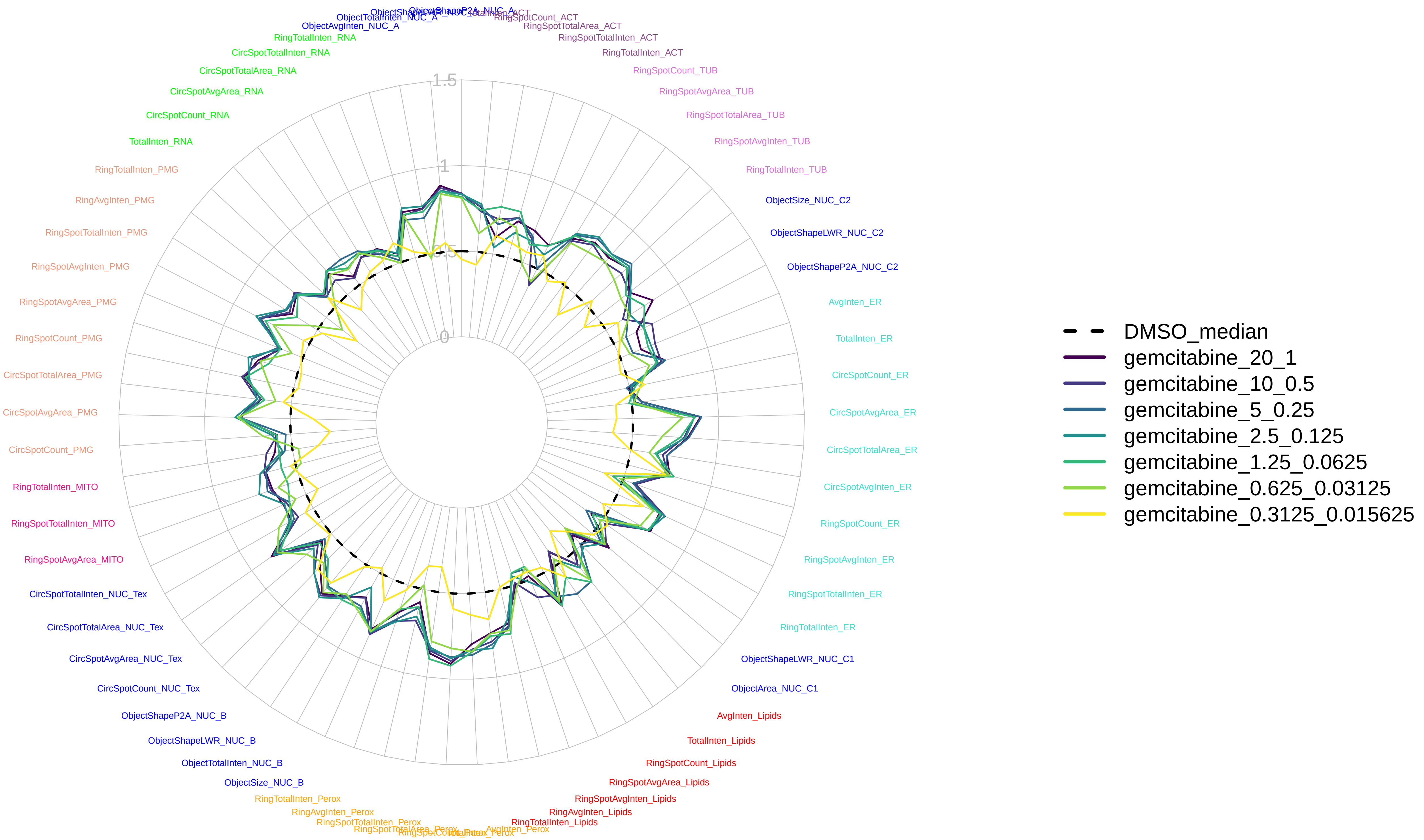

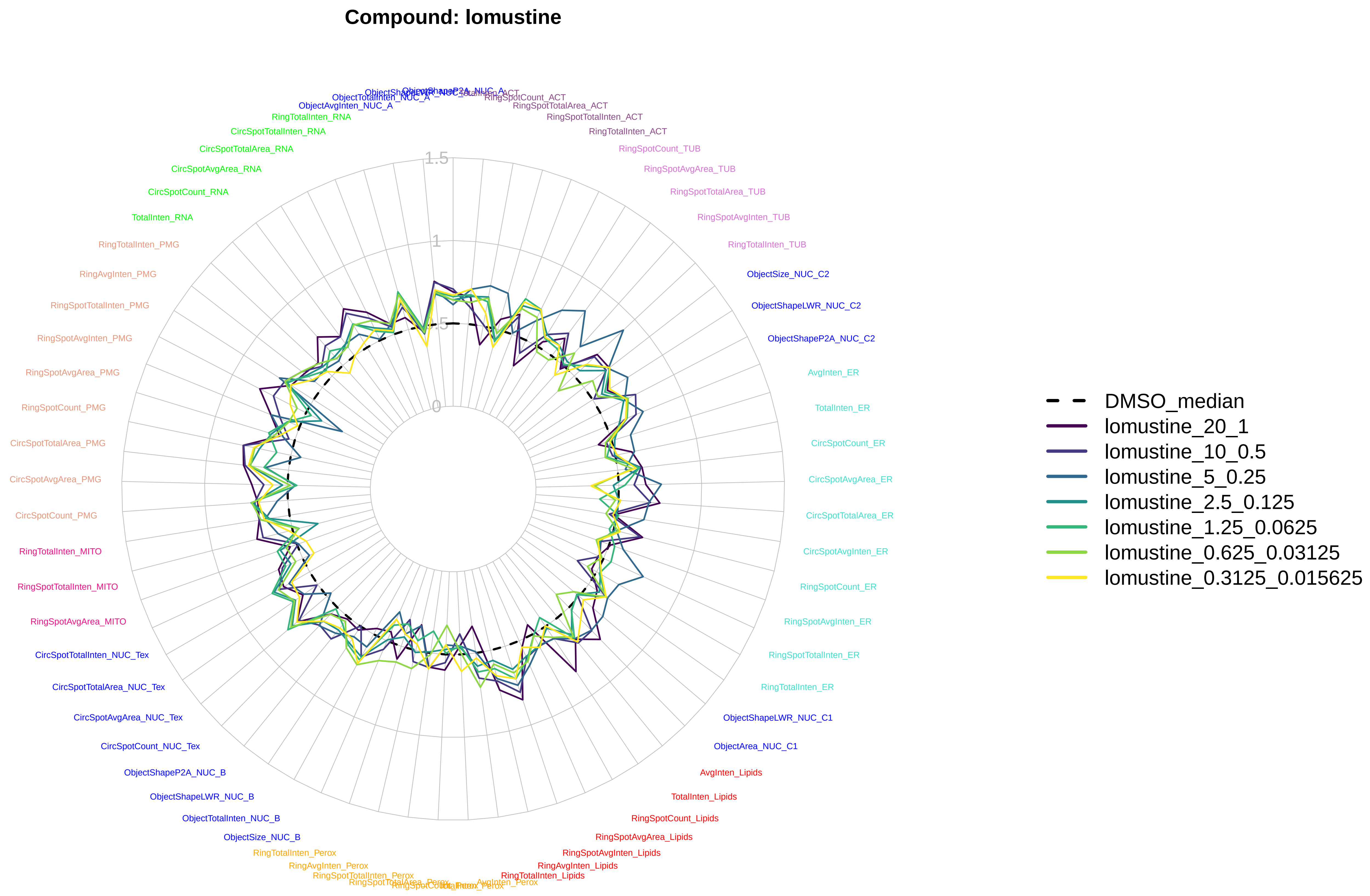

Compound: lomustine
1.5
1
.5
0
DMSO_median
lomustine_20_1
lomustine_10_0.5
lomustine_5_0.25
lomustine_2.5_0.125
lomustine_1.25_0.0625
lomustine_0.625_0.03125
lomustine_0.3125_0.015625
RingSpotCount_ACT
RingSpotTotalArea_ACT
RingSpotTotalInten_ACT
RingTotalInten_ACT
RingSpotCount_TUB
RingSpotAvgArea_TUB
RingSpotTotalArea_TUB
RingSpotAvgInten_TUB
RingTotalInten_TUB
ObjectSize_NUC_C2
ObjectShapeLWR_NUC_C2
ObjectShapeP2A_NUC_C2
AvgInten_ER
TotalInten_ER
CircSpotCount_ER
CircSpotAvgArea_ER
CircSpotTotalArea_ER
CircSpotAvgInten_ER
RingSpotCount_ER
RingSpotAvgInten_ER
RingSpotTotalInten_ER
RingTotalInten_ER
ObjectShapeLWR_NUC_C1
ObjectArea_NUC_C1
AvgInten_Lipids
TotalInten_Lipids
RingSpotCount_Lipids
RingSpotAvgArea_Lipids
RingSpotAvgInten_Lipids
RingAvgInten_Lipids
RingTotalInten_Lipids
AvgInten_Perox
RingTotalInten_Perox
RingAvgInten_Perox
RingSpotTotalInten_Perox
ObjectSize_NUC_B
ObjectTotalInten_NUC_B
ObjectShapeLWR_NUC_B
ObjectShapeP2A_NUC_B
CircSpotCount_NUC_Tex
CircSpotAvgArea_NUC_Tex
CircSpotTotalArea_NUC_Tex
CircSpotTotalInten_NUC_Tex
RingSpotAvgArea_MITO
RingSpotTotalInten_MITO
RingTotalInten_MITO
CircSpotCount_PMG
CircSpotAvgArea_PMG
CircSpotTotalArea_PMG
RingSpotCount_PMG
RingSpotAvgArea_PMG
RingSpotAvgInten_PMG
RingSpotTotalInten_PMG
RingAvgInten_PMG
RingTotalInten_PMG
TotalInten_RNA
CircSpotCount_RNA
CircSpotAvgArea_RNA
CircSpotTotalArea_RNA
CircSpotTotalInten_RNA
RingTotalInten_RNA
ObjectAvgInten_NUC_A
ObjectTotalInten_NUC_A

Compound: haloperidol

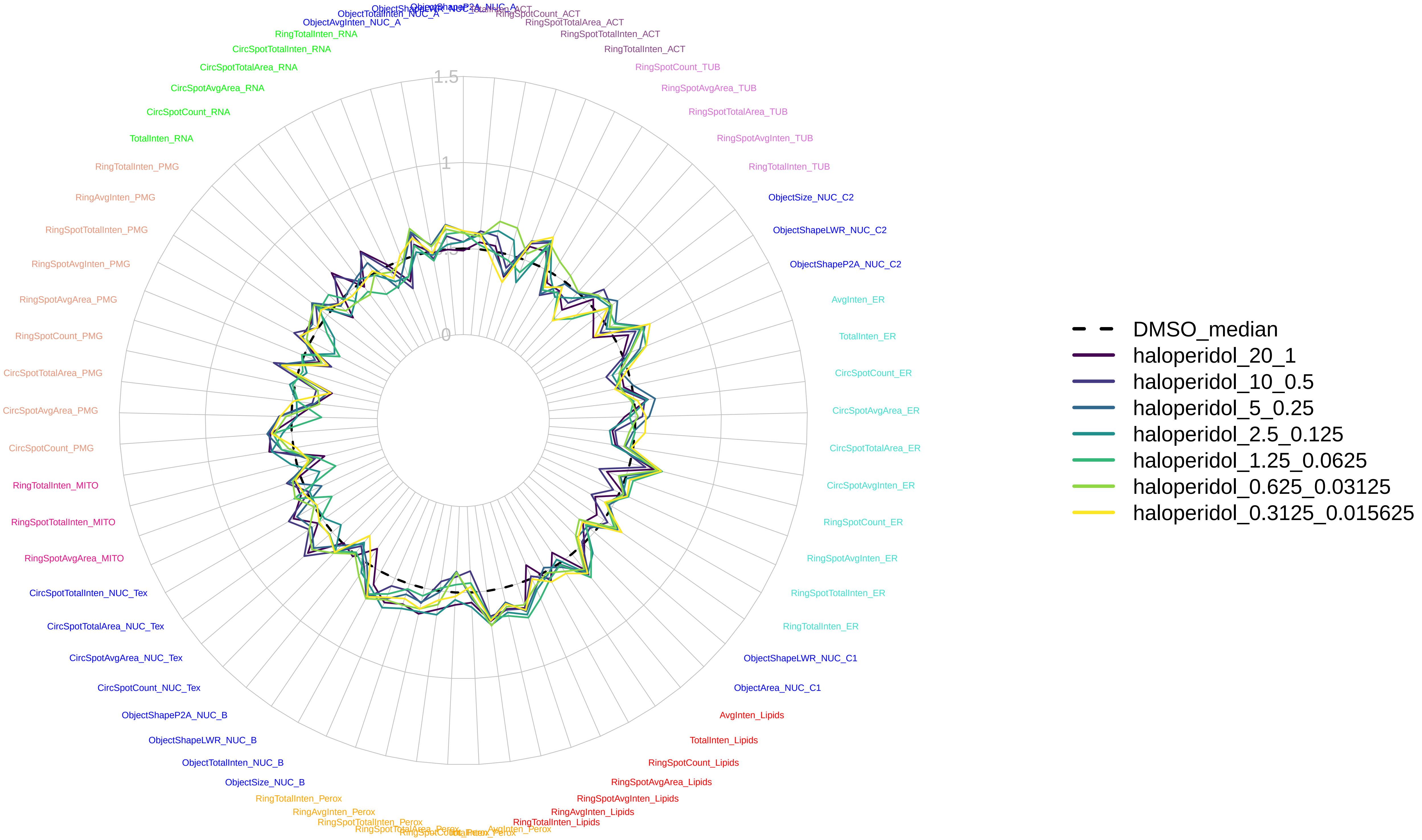

Compound: mepacrine

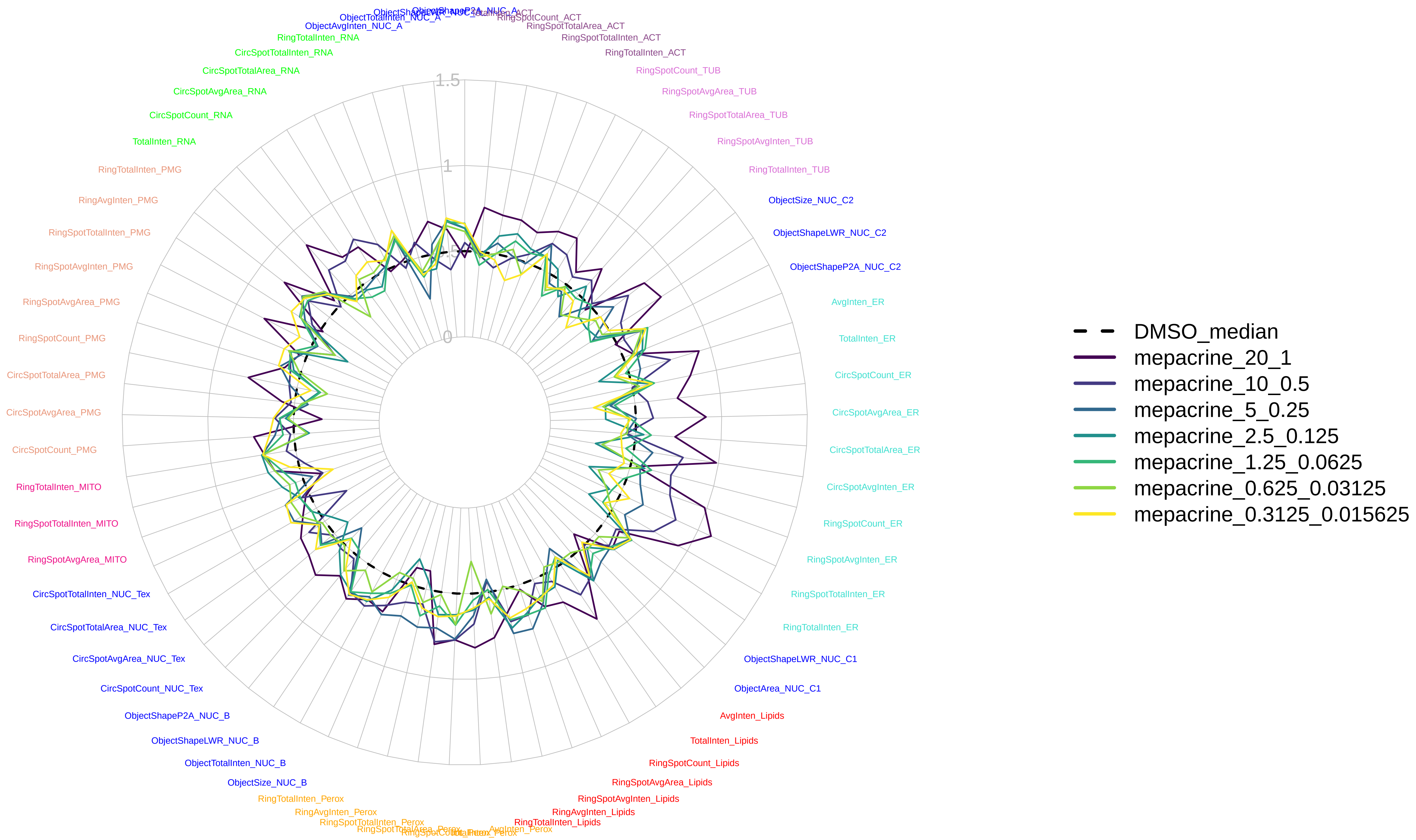

Compound: dynasore

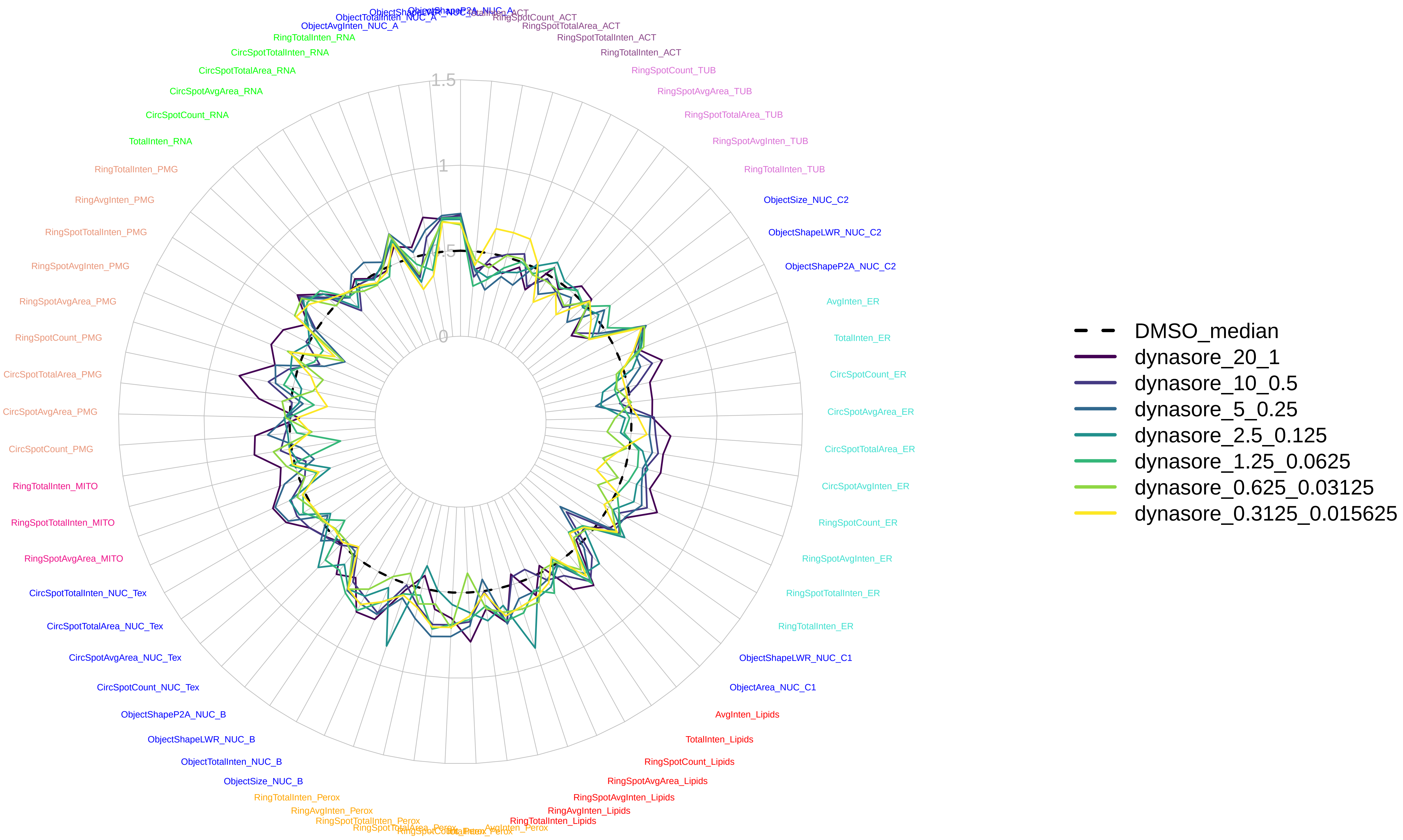

Compound: tetrandrine

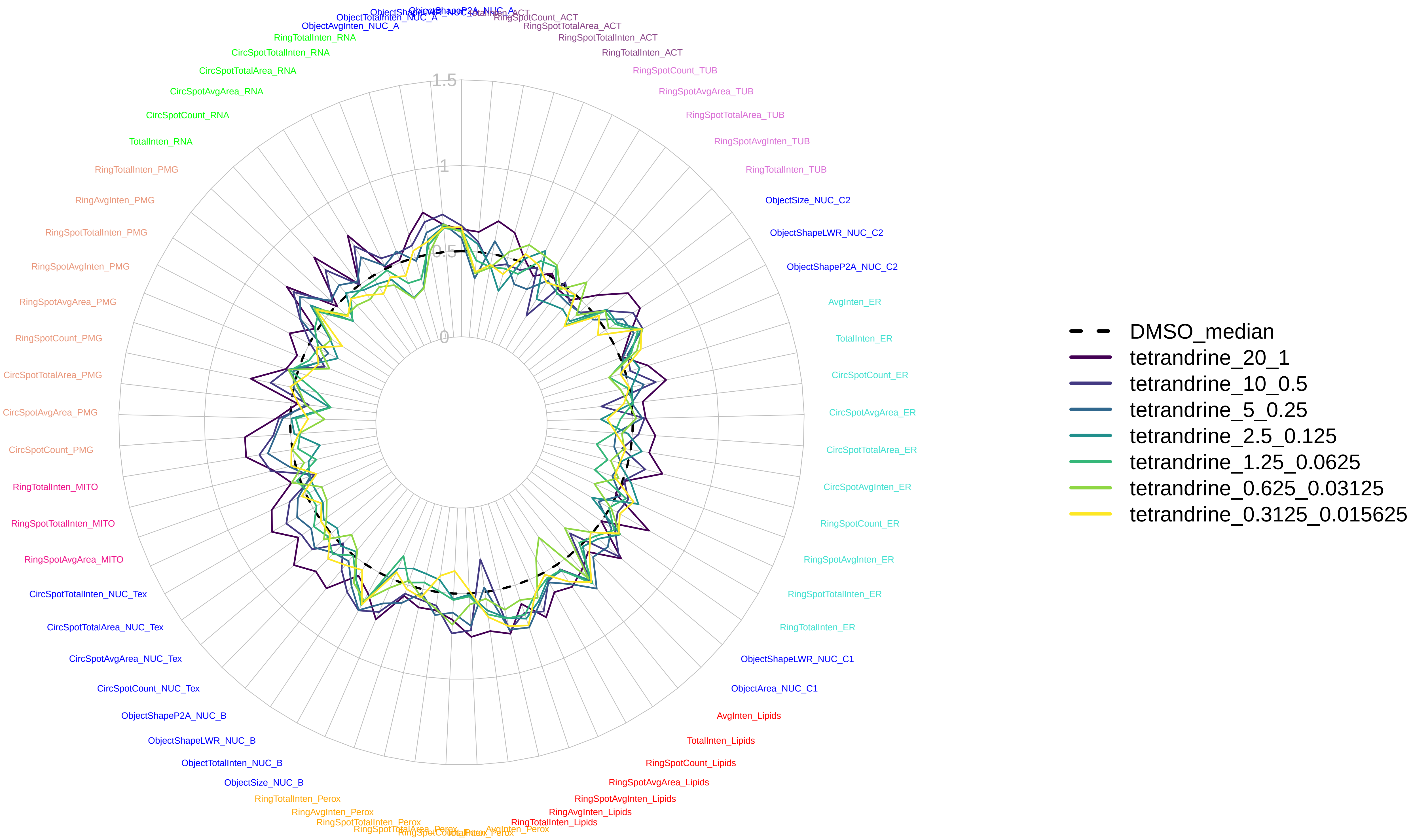

Compound: cytochalasin−d

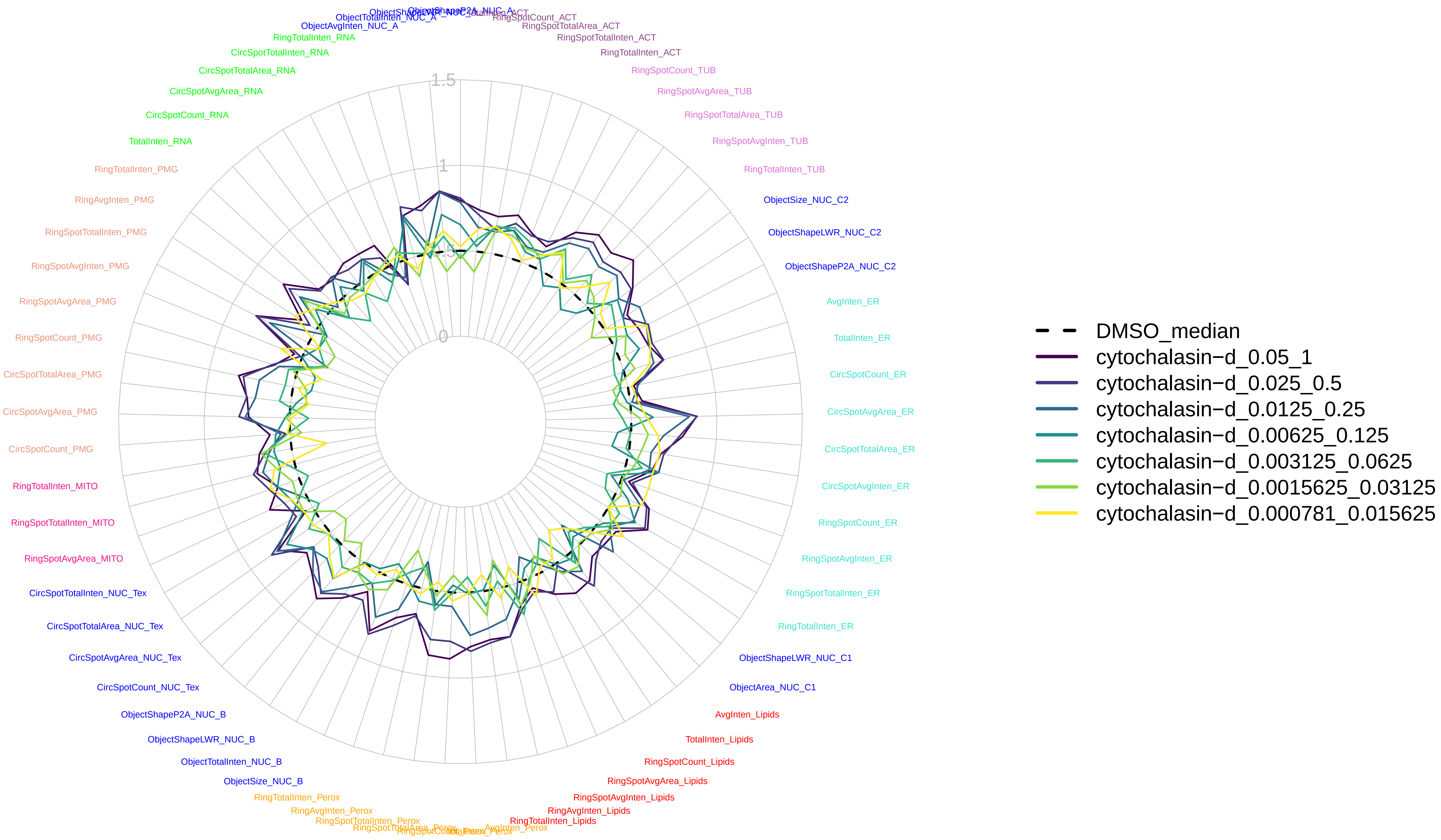

Compound: tanespimycin

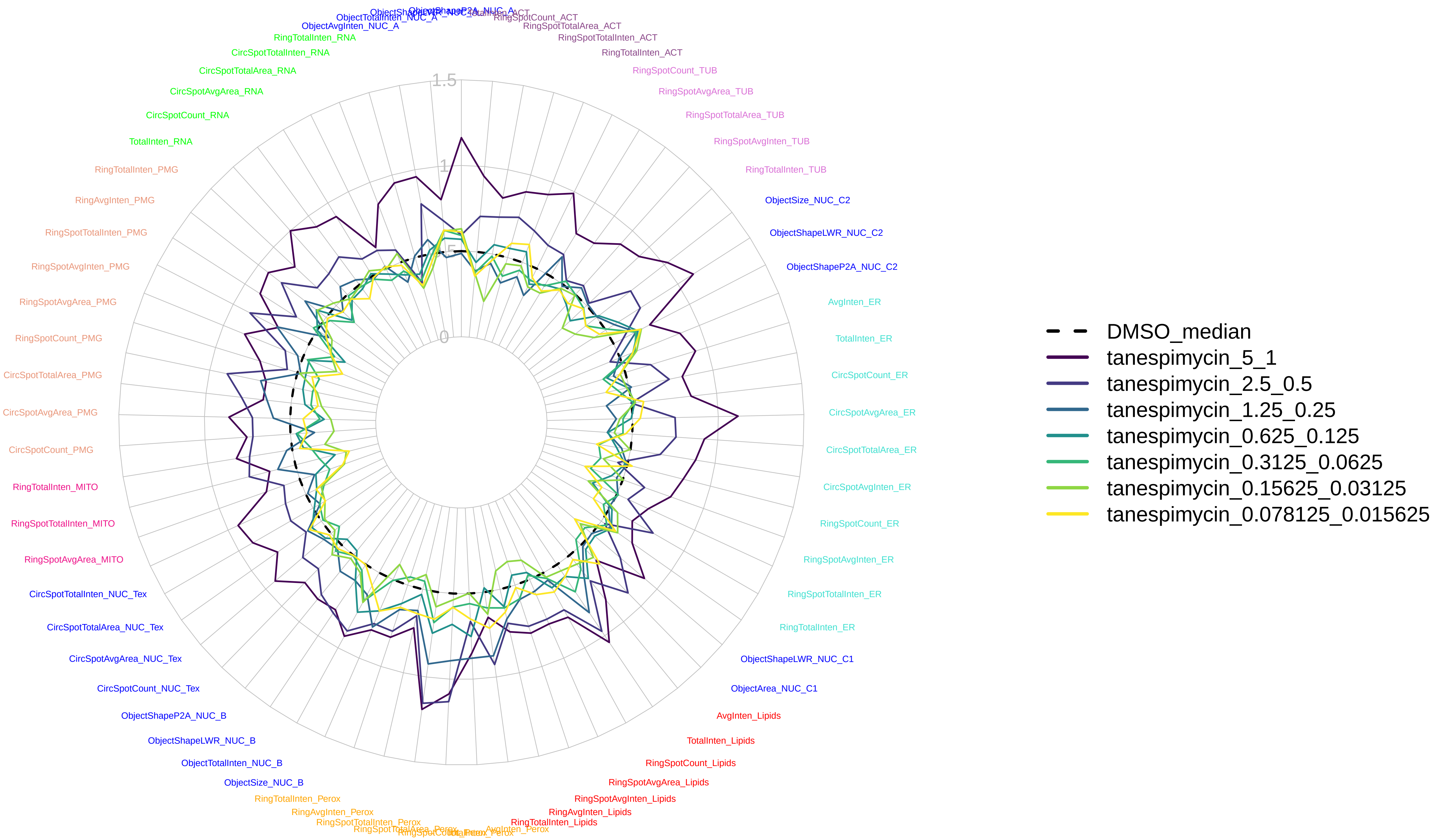

Compound: MG−101

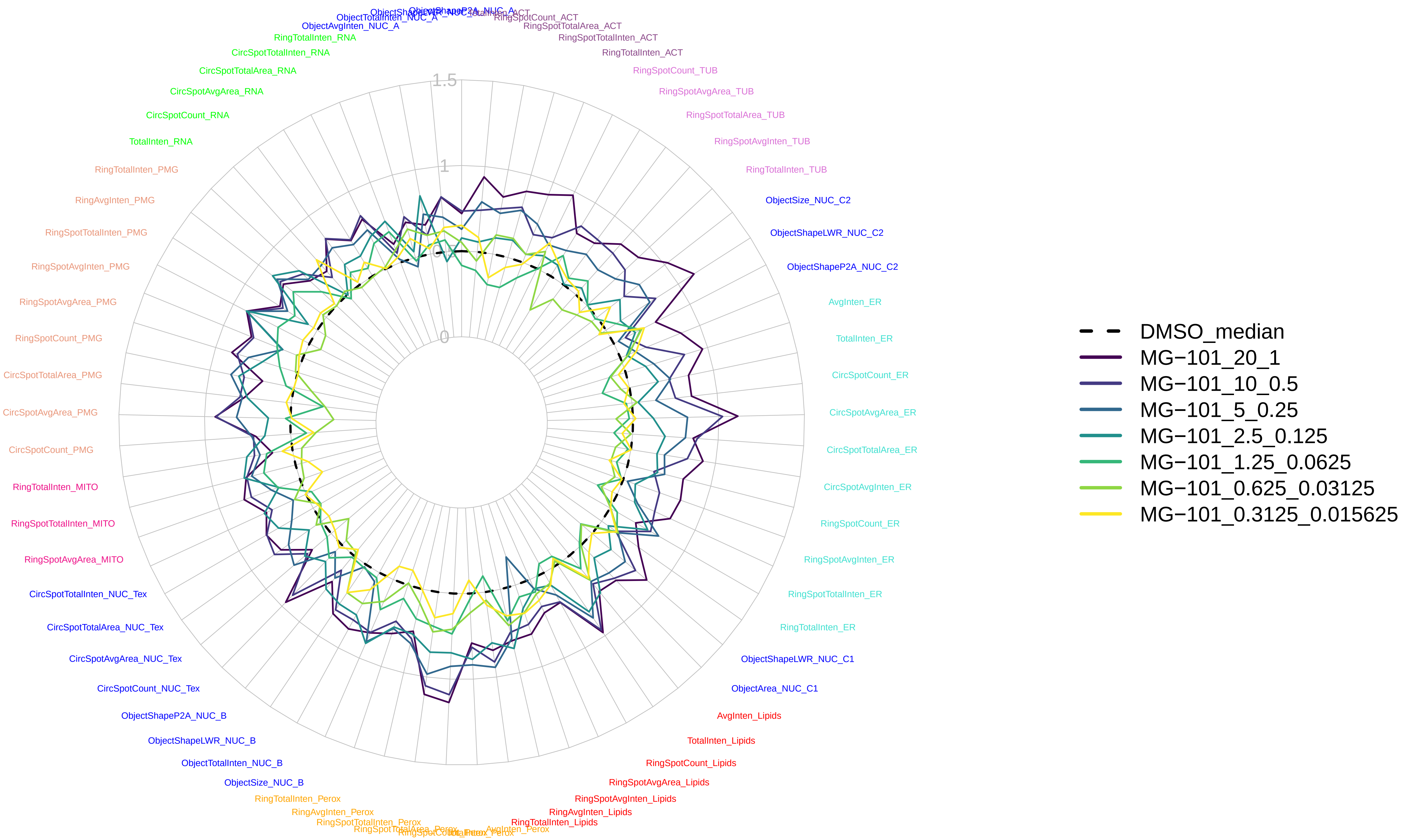

Compound: tunicamycin

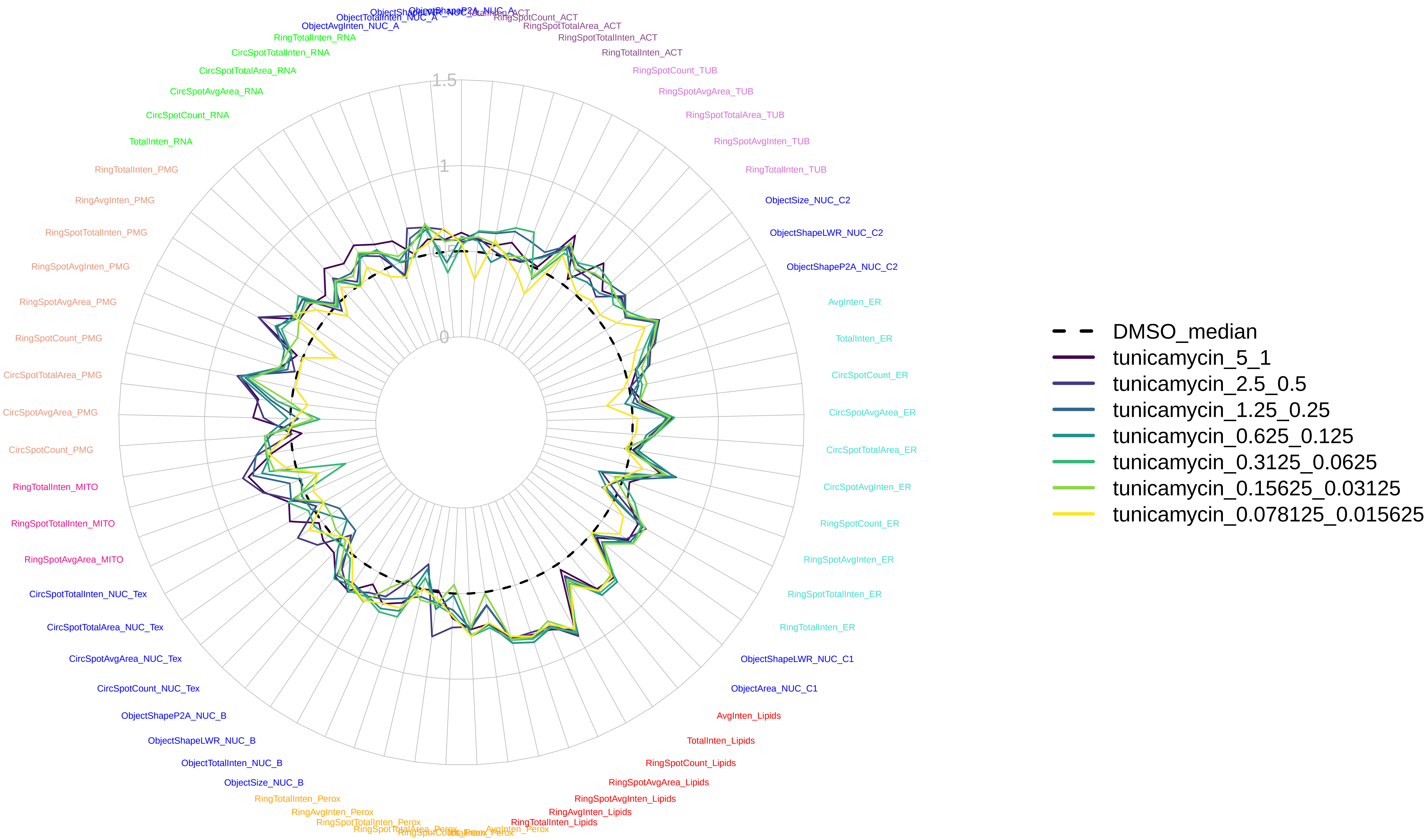

Compound: picropodophyllin

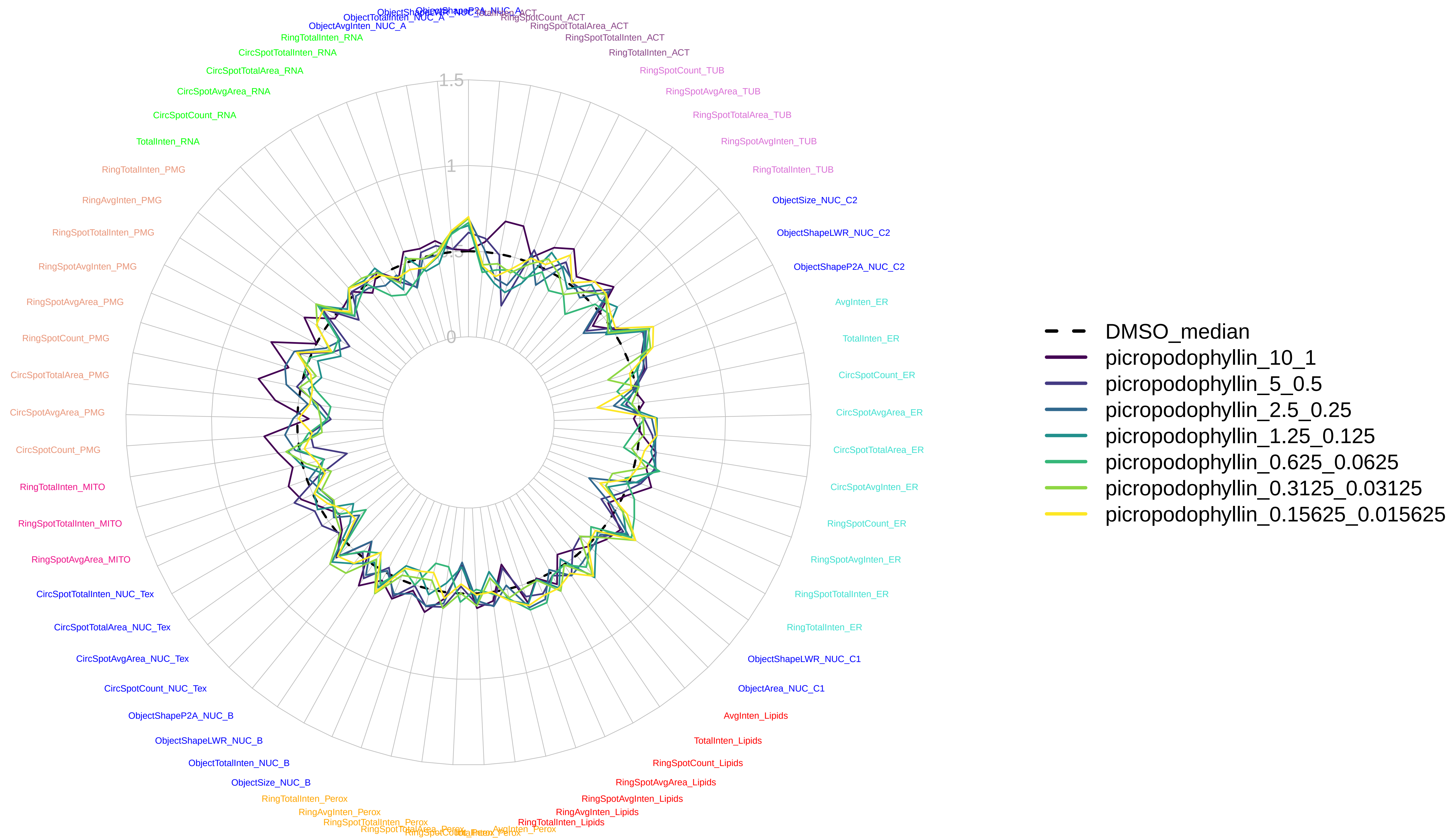

Compound: deforolimus

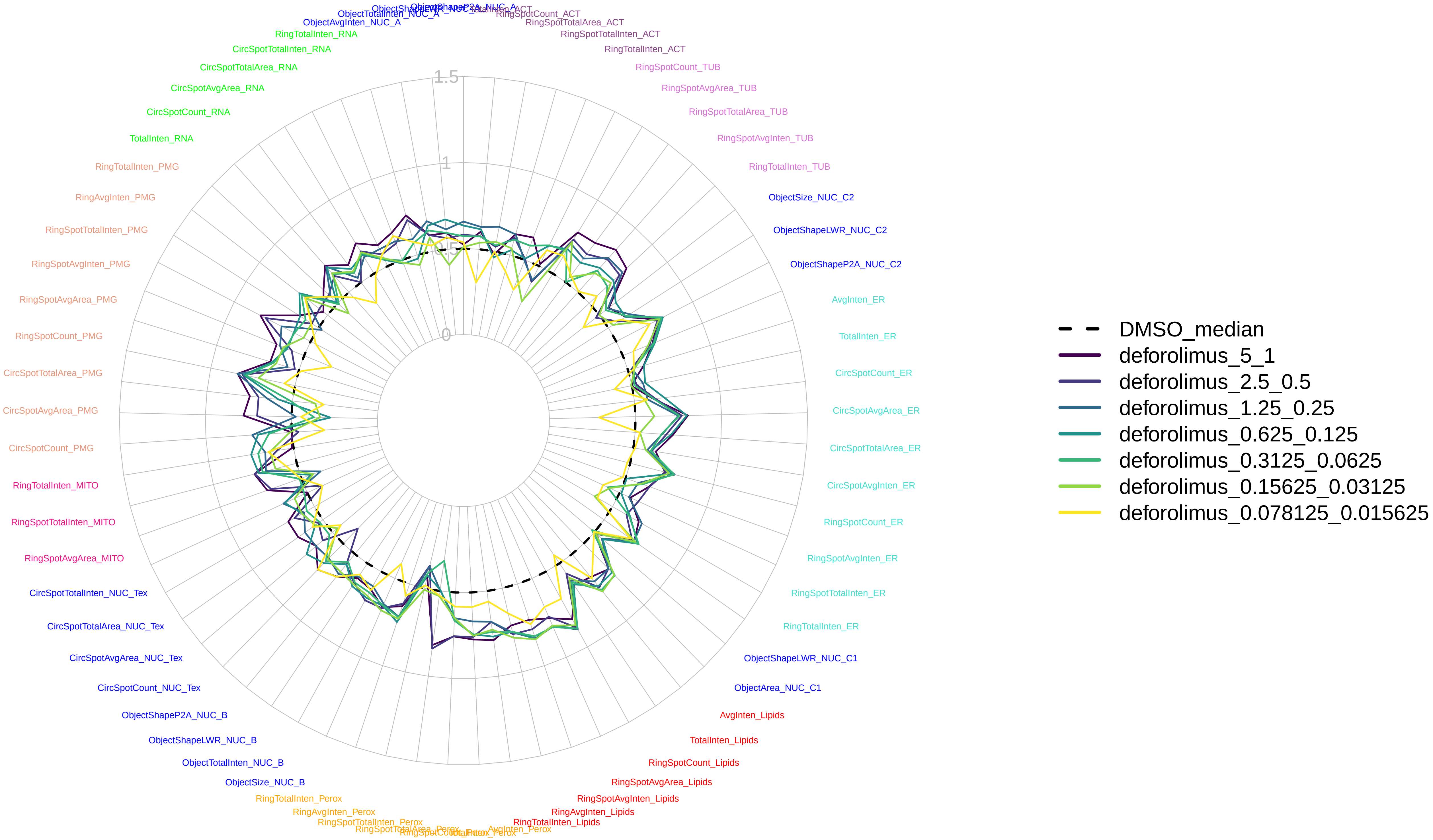

Compound: FCCP

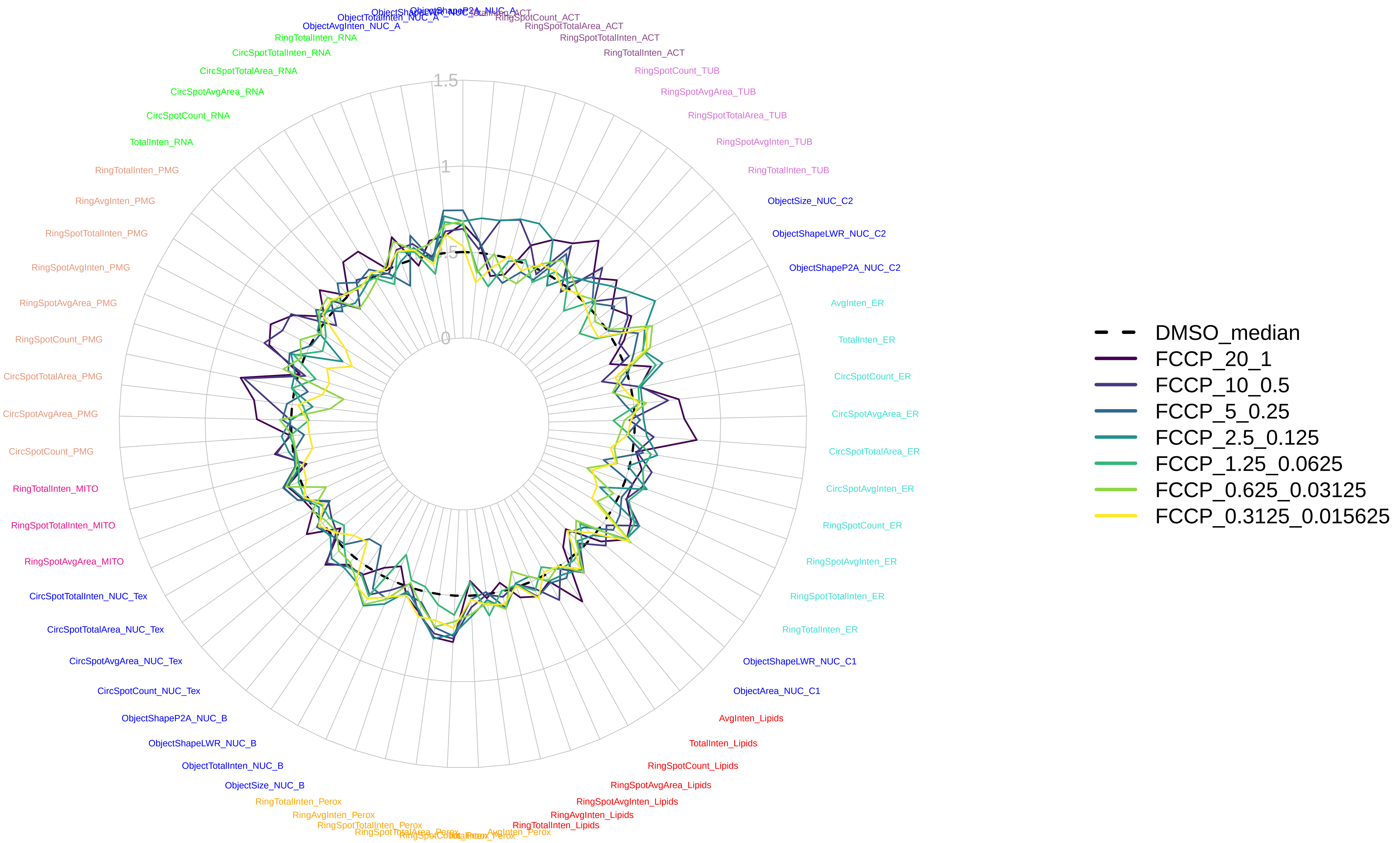

Compound: daunorubicin

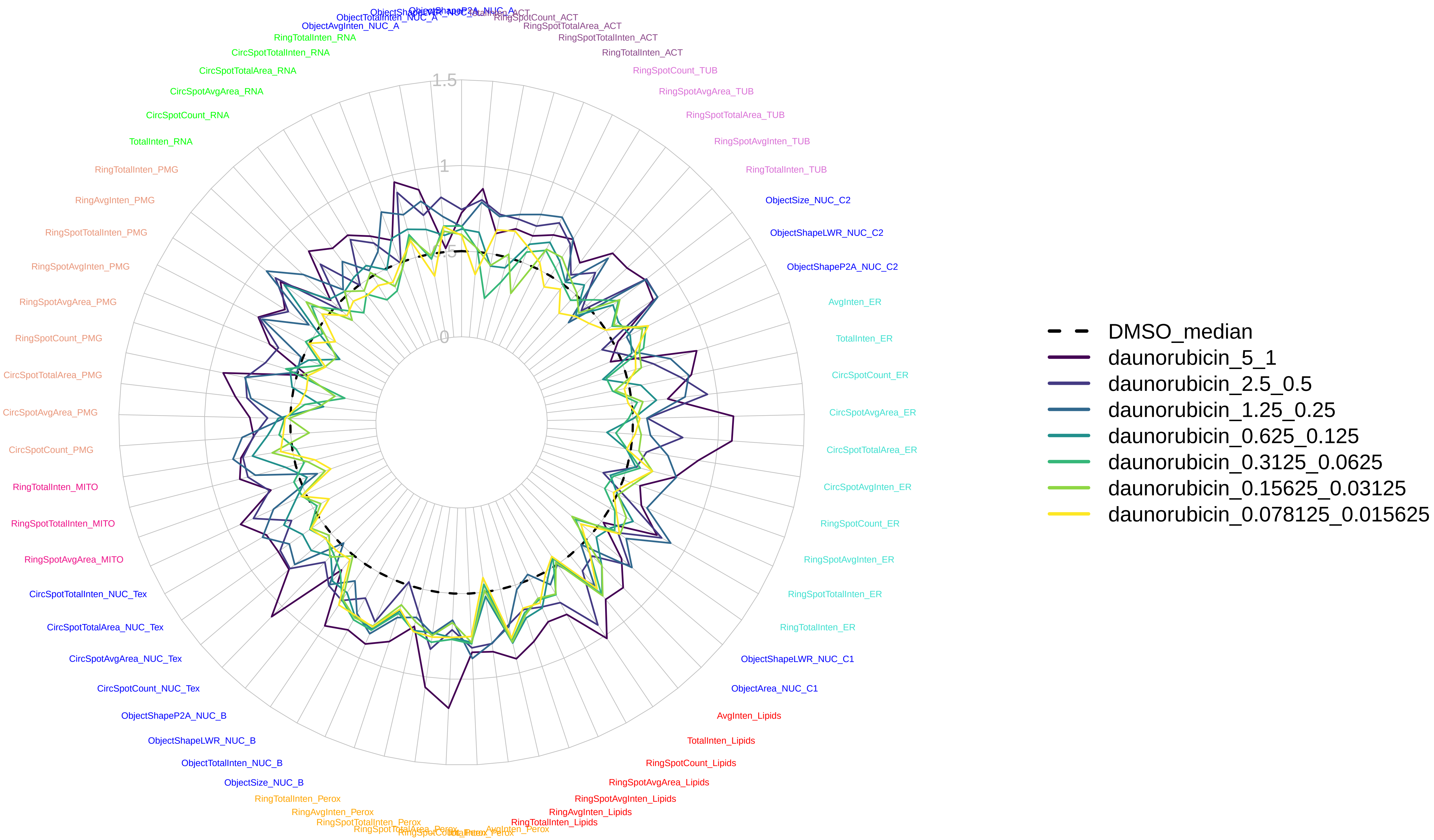

Compound: mitoxantrone

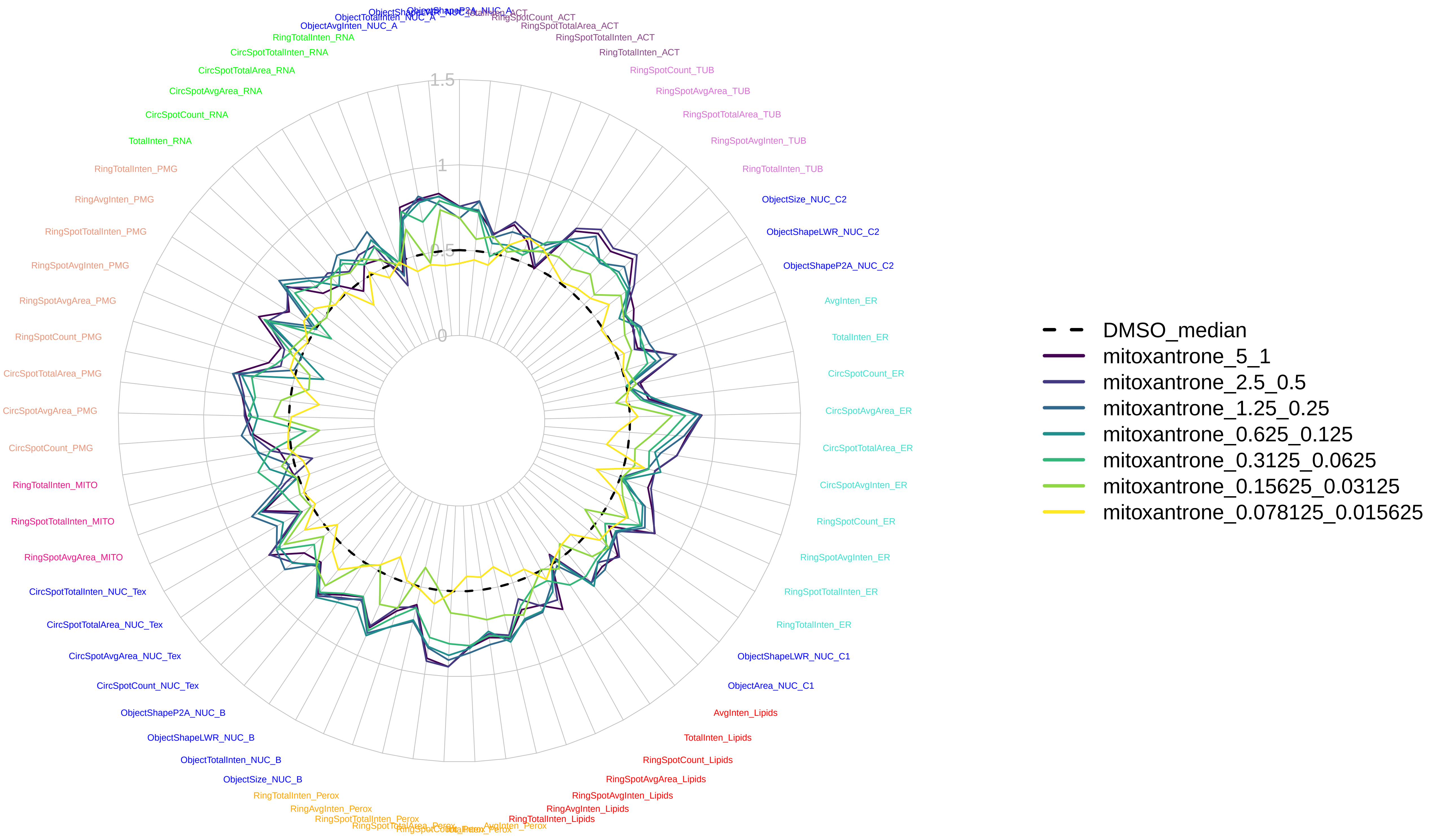

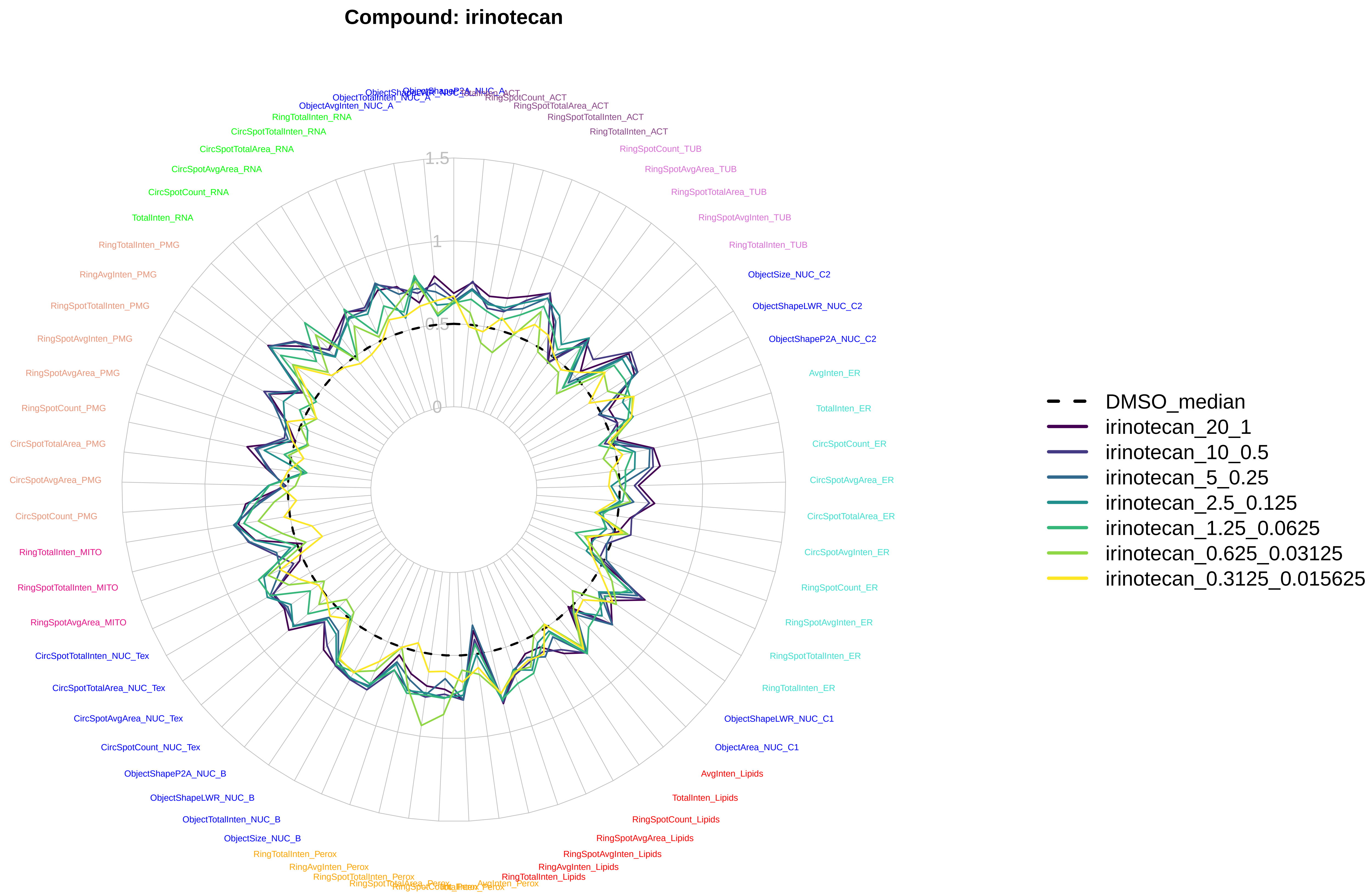
Compound: irinotecan
1.5
1
0.5
0
ObjectTotalInten_NUC_A
ObjectAvgInten_NUC_A
RingTotalInten_RNA
CircSpotTotalInten_RNA
CircSpotTotalArea_RNA
CircSpotAvgArea_RNA
CircSpotCount_RNA
TotalInten_RNA
RingTotalInten_PMG
RingAvgInten_PMG
RingSpotTotalInten_PMG
RingSpotAvgInten_PMG
RingSpotAvgArea_PMG
RingSpotCount_PMG
CircSpotTotalArea_PMG
CircSpotAvgArea_PMG
CircSpotCount_PMG
RingTotalInten_MITO
RingSpotTotalInten_MITO
RingSpotAvgArea_MITO
CircSpotTotalInten_NUC_Tex
CircSpotTotalArea_NUC_Tex
CircSpotAvgArea_NUC_Tex
CircSpotCount_NUC_Tex
ObjectShapeP2A_NUC_B
ObjectShapeLWR_NUC_B
ObjectTotalInten_NUC_B
ObjectSize_NUC_B
RingTotalInten_Perox
RingAvgInten_Perox
RingSpotTotalInten_Perox
RingSpotCount_ACT
RingSpotTotalArea_ACT
RingSpotTotalInten_ACT
RingTotalInten_ACT
RingSpotCount_TUB
RingSpotAvgArea_TUB
RingSpotTotalArea_TUB
RingSpotAvgInten_TUB
RingTotalInten_TUB
ObjectSize_NUC_C2
ObjectShapeLWR_NUC_C2
ObjectShapeP2A_NUC_C2
AvgInten_ER
TotalInten_ER
CircSpotCount_ER
CircSpotAvgArea_ER
CircSpotTotalArea_ER
CircSpotAvgInten_ER
RingSpotCount_ER
RingSpotAvgInten_ER
RingSpotTotalInten_ER
RingTotalInten_ER
ObjectShapeLWR_NUC_C1
ObjectArea_NUC_C1
AvgInten_Lipids
TotalInten_Lipids
RingSpotCount_Lipids
RingSpotAvgArea_Lipids
RingSpotAvgInten_Lipids
RingAvgInten_Lipids
RingTotalInten_Lipids
DMSO_median
irinotecan_20_1
irinotecan_10_0.5
irinotecan_5_0.25
irinotecan_2.5_0.125
irinotecan_1.25_0.0625
irinotecan_0.625_0.03125
irinotecan_0.3125_0.015625

Compound: bendamustine

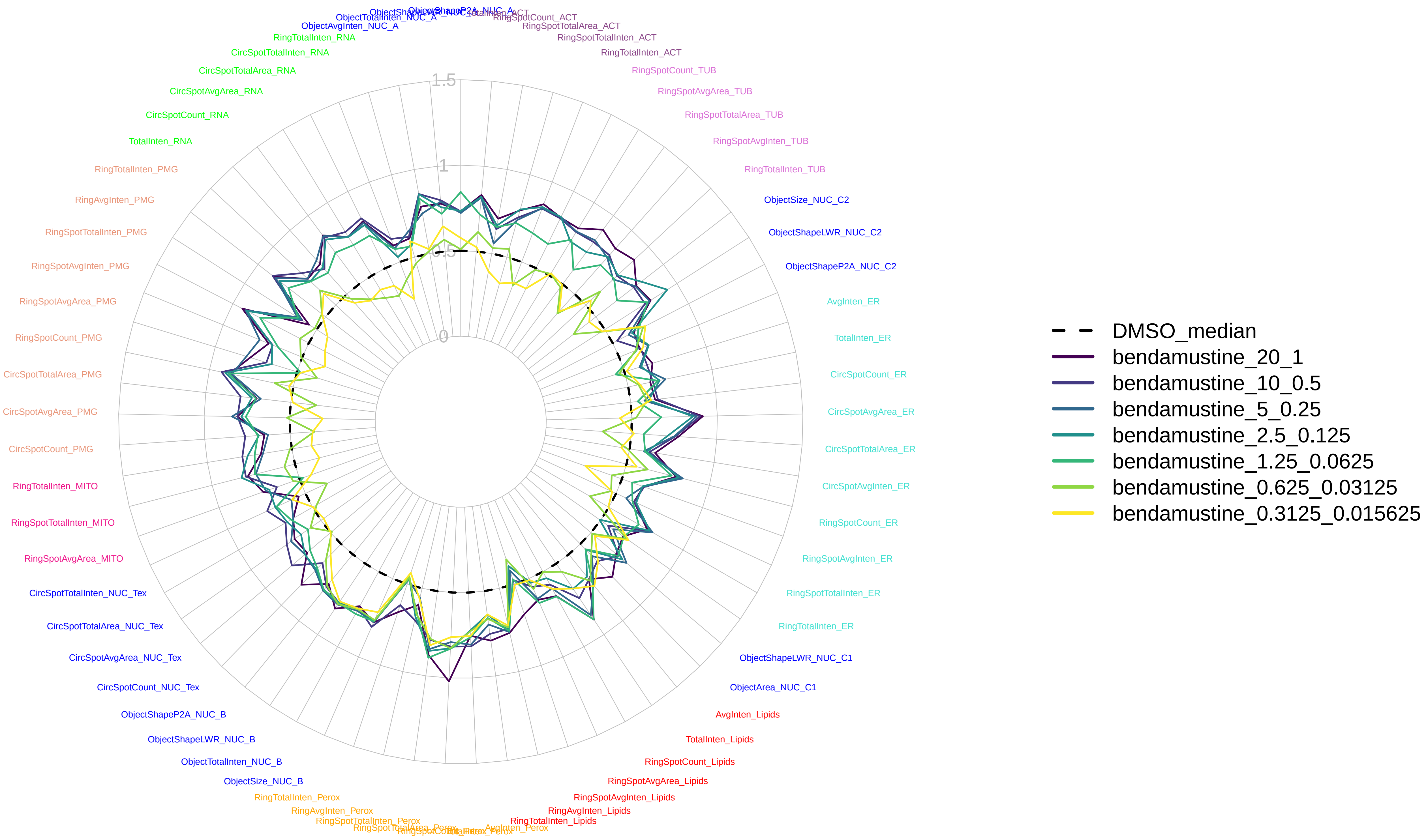

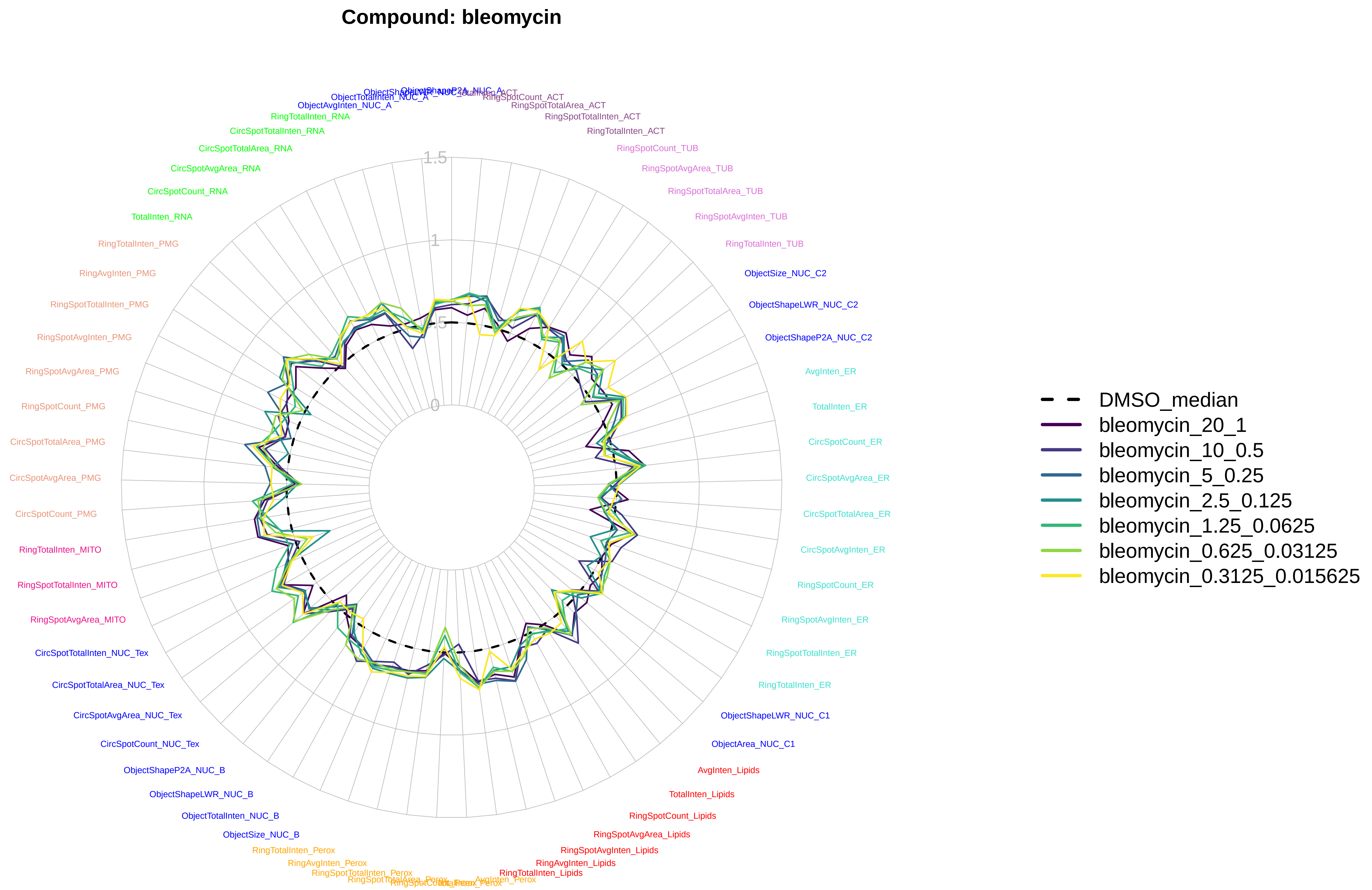
Compound: bleomycin
DMSO_median
bleomycin_20_1
bleomycin_10_0.5
bleomycin_5_0.25
bleomycin_2.5_0.125
bleomycin_1.25_0.0625
bleomycin_0.625_0.03125
bleomycin_0.3125_0.015625
1.5
1
.5
0
RingSpotCount_ACT
RingSpotTotalArea_ACT
RingSpotTotalInten_ACT
RingTotalInten_ACT
RingSpotCount_TUB
RingSpotAvgArea_TUB
RingSpotTotalArea_TUB
RingSpotAvgInten_TUB
RingTotalInten_TUB
ObjectSize_NUC_C2
ObjectShapeLWR_NUC_C2
ObjectShapeP2A_NUC_C2
AvgInten_ER
TotalInten_ER
CircSpotCount_ER
CircSpotAvgArea_ER
CircSpotTotalArea_ER
CircSpotAvgInten_ER
RingSpotCount_ER
RingSpotAvgInten_ER
RingSpotTotalInten_ER
RingTotalInten_ER
ObjectShapeLWR_NUC_C1
ObjectArea_NUC_C1
AvgInten_Lipids
TotalInten_Lipids
RingSpotCount_Lipids
RingSpotAvgArea_Lipids
RingSpotAvgInten_Lipids
RingAvgInten_Lipids
RingTotalInten_Lipids
AvgInten_Perox
RingTotalInten_Perox
RingAvgInten_Perox
RingSpotTotalInten_Perox
ObjectSize_NUC_B
ObjectTotalInten_NUC_B
ObjectShapeLWR_NUC_B
ObjectShapeP2A_NUC_B
CircSpotCount_NUC_Tex
CircSpotAvgArea_NUC_Tex
CircSpotTotalArea_NUC_Tex
CircSpotTotalInten_NUC_Tex
RingSpotAvgArea_MITO
RingSpotTotalInten_MITO
RingTotalInten_MITO
CircSpotCount_PMG
CircSpotAvgArea_PMG
CircSpotTotalArea_PMG
RingSpotCount_PMG
RingSpotAvgArea_PMG
RingSpotAvgInten_PMG
RingSpotTotalInten_PMG
RingAvgInten_PMG
RingTotalInten_PMG
TotalInten_RNA
CircSpotCount_RNA
CircSpotAvgArea_RNA
CircSpotTotalArea_RNA
CircSpotTotalInten_RNA
RingTotalInten_RNA
ObjectAvgInten_NUC_A
ObjectTotalInten_NUC_A

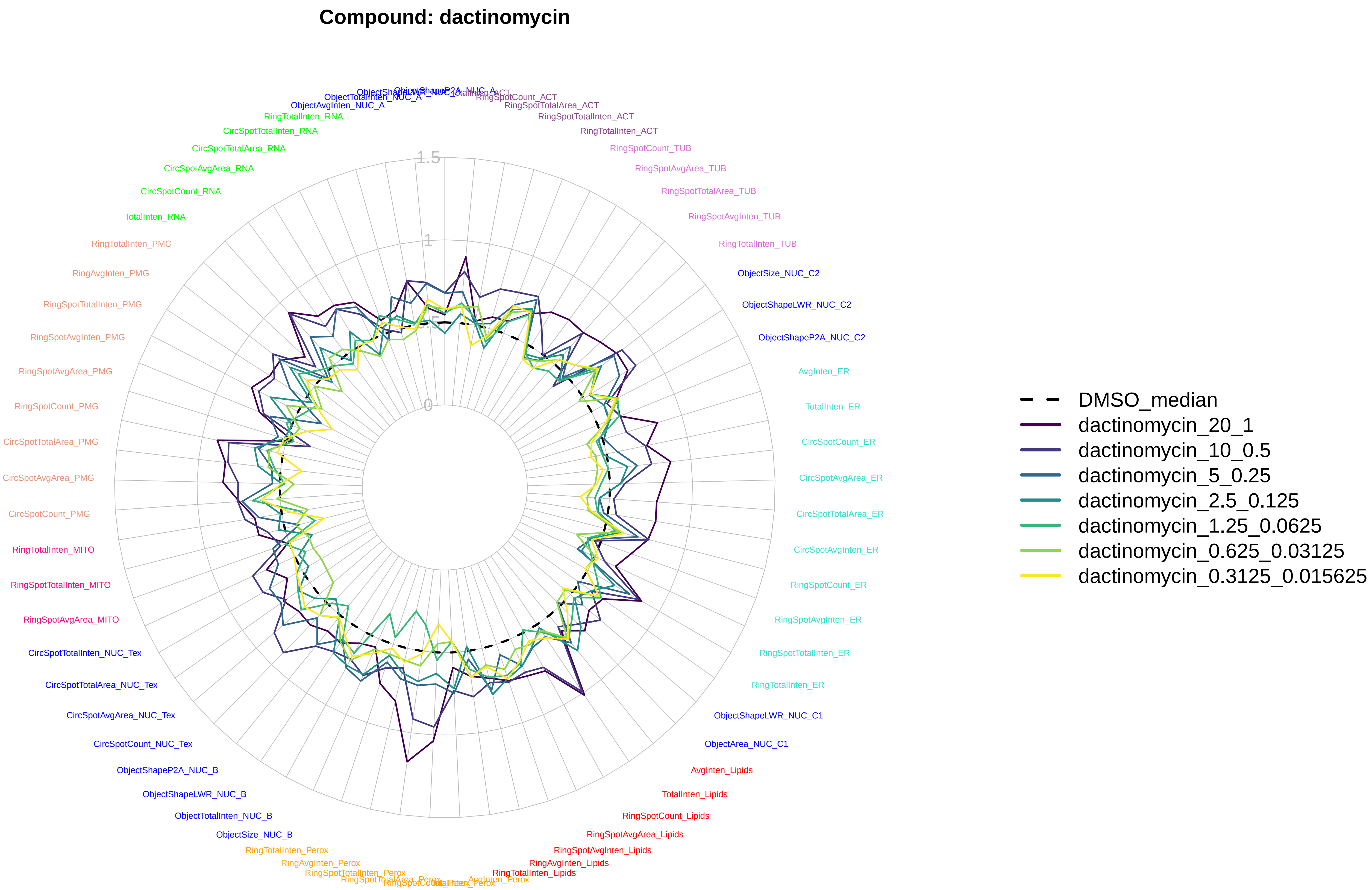

Compound: dactinomycin
ObjectShapeP2A_NUC_A
ObjectTotalInten_NUC_A
ObjectAvgInten_NUC_A
RingSpotCount_ACT
RingSpotTotalArea_ACT
RingSpotTotalInten_ACT
RingTotalInten_ACT
RingSpotCount_TUB
RingSpotAvgArea_TUB
RingSpotTotalArea_TUB
RingSpotAvgInten_TUB
RingTotalInten_TUB
ObjectSize_NUC_C2
ObjectShapeLWR_NUC_C2
ObjectShapeP2A_NUC_C2
AvgInten_ER
TotalInten_ER
CircSpotCount_ER
CircSpotAvgArea_ER
CircSpotTotalArea_ER
CircSpotAvgInten_ER
RingSpotCount_ER
RingSpotAvgInten_ER
RingSpotTotalInten_ER
RingTotalInten_ER
ObjectShapeLWR_NUC_C1
ObjectArea_NUC_C1
AvgInten_Lipids
TotalInten_Lipids
RingSpotCount_Lipids
RingSpotAvgArea_Lipids
RingSpotAvgInten_Lipids
RingAvgInten_Lipids
RingTotalInten_Lipids
AvgInten_Perox
RingSpotTotalInten_Perox
RingAvgInten_Perox
RingTotalInten_Perox
ObjectSize_NUC_B
ObjectTotalInten_NUC_B
ObjectShapeLWR_NUC_B
ObjectShapeP2A_NUC_B
CircSpotCount_NUC_Tex
CircSpotAvgArea_NUC_Tex
CircSpotTotalArea_NUC_Tex
CircSpotTotalInten_NUC_Tex
RingSpotAvgArea_MITO
RingSpotTotalInten_MITO
RingTotalInten_MITO
CircSpotCount_PMG
CircSpotAvgArea_PMG
CircSpotTotalArea_PMG
RingSpotCount_PMG
RingSpotAvgArea_PMG
RingSpotAvgInten_PMG
RingSpotTotalInten_PMG
RingAvgInten_PMG
RingTotalInten_PMG
TotalInten_RNA
CircSpotCount_RNA
CircSpotAvgArea_RNA
CircSpotTotalArea_RNA
CircSpotTotalInten_RNA
RingTotalInten_RNA
1.5
1
0
DMSO_median
dactinomycin_20_1
dactinomycin_10_0.5
dactinomycin_5_0.25
dactinomycin_2.5_0.125
dactinomycin_1.25_0.0625
dactinomycin_0.625_0.03125
dactinomycin_0.3125_0.015625

Compound: fluvastatin

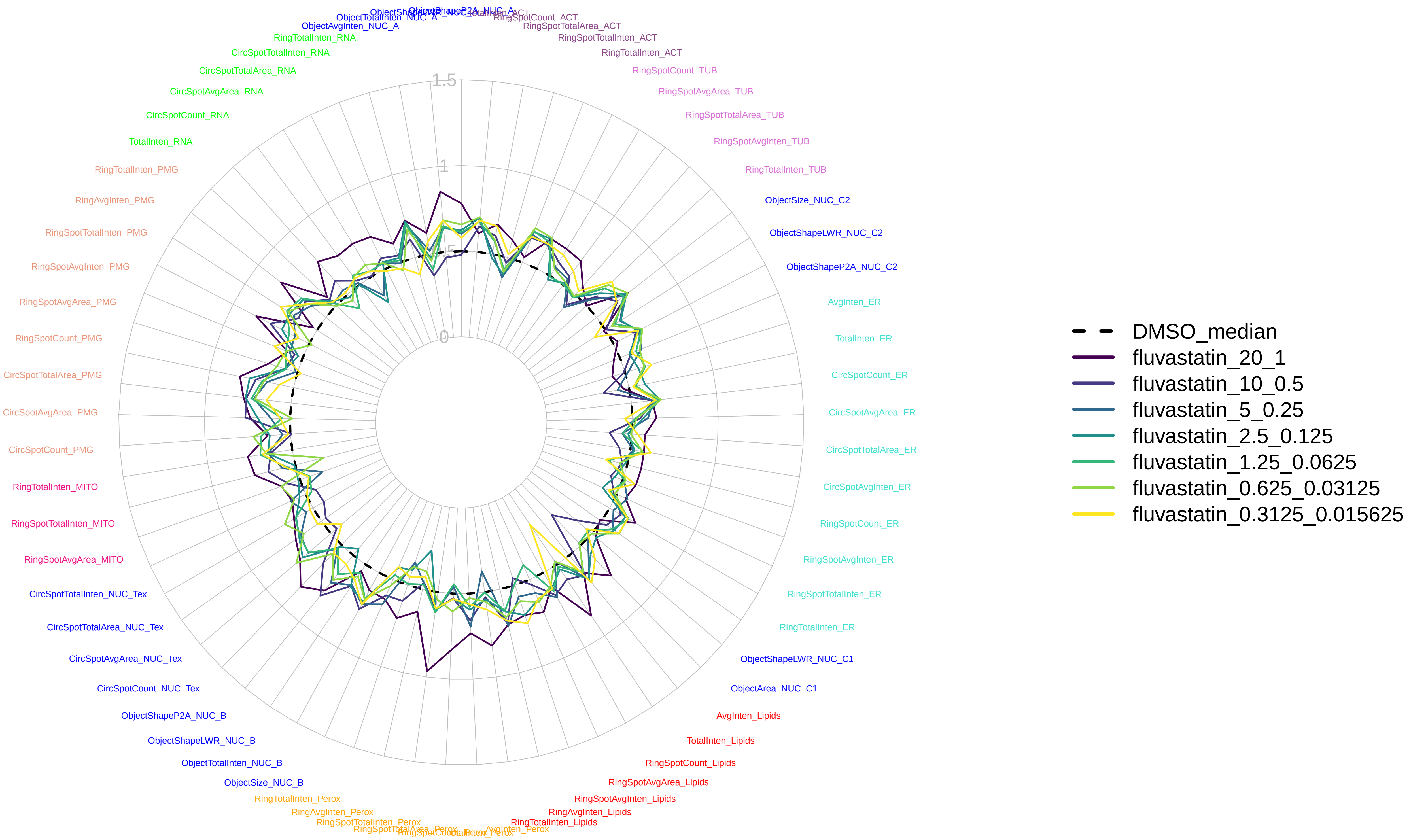

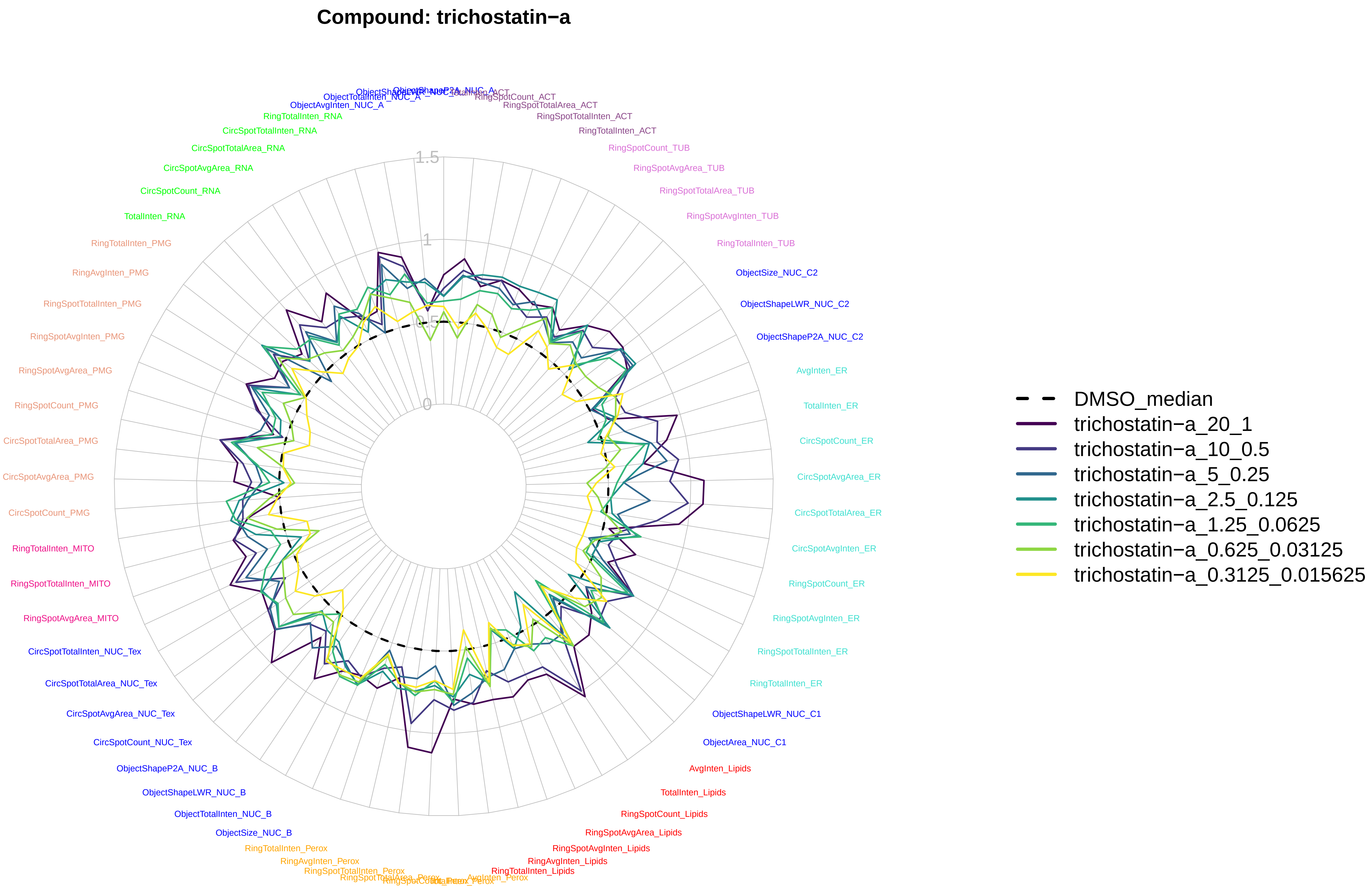
Compound: trichostatin−a
1.5
1
0.5
0
ObjectShapeP2A_NUC_A
ObjectTotalInten_NUC_A
ObjectAvgInten_NUC_A
RingSpotCount_ACT
RingSpotTotalArea_ACT
RingSpotTotalInten_ACT
RingTotalInten_ACT
RingSpotCount_TUB
RingSpotAvgArea_TUB
RingSpotTotalArea_TUB
RingSpotAvgInten_TUB
RingTotalInten_TUB
ObjectSize_NUC_C2
ObjectShapeLWR_NUC_C2
ObjectShapeP2A_NUC_C2
AvgInten_ER
TotalInten_ER
CircSpotCount_ER
CircSpotAvgArea_ER
CircSpotTotalArea_ER
CircSpotAvgInten_ER
RingSpotCount_ER
RingSpotAvgInten_ER
RingSpotTotalInten_ER
RingTotalInten_ER
ObjectShapeLWR_NUC_C1
ObjectArea_NUC_C1
AvgInten_Lipids
TotalInten_Lipids
RingSpotCount_Lipids
RingSpotAvgArea_Lipids
RingSpotAvgInten_Lipids
RingAvgInten_Lipids
RingTotalInten_Lipids
AvgInten_Perox
RingTotalInten_Perox
RingAvgInten_Perox
RingSpotTotalInten_Perox
ObjectSize_NUC_B
ObjectTotalInten_NUC_B
ObjectShapeLWR_NUC_B
ObjectShapeP2A_NUC_B
CircSpotCount_NUC_Tex
CircSpotAvgArea_NUC_Tex
CircSpotTotalArea_NUC_Tex
CircSpotTotalInten_NUC_Tex
RingSpotAvgArea_MITO
RingSpotTotalInten_MITO
RingTotalInten_MITO
CircSpotCount_PMG
CircSpotAvgArea_PMG
CircSpotTotalArea_PMG
RingSpotCount_PMG
RingSpotAvgArea_PMG
RingSpotAvgInten_PMG
RingSpotTotalInten_PMG
RingAvgInten_PMG
RingTotalInten_PMG
TotalInten_RNA
CircSpotCount_RNA
CircSpotAvgArea_RNA
CircSpotTotalArea_RNA
CircSpotTotalInten_RNA
RingTotalInten_RNA
DMSO_median
trichostatin−a_20_1
trichostatin−a_10_0.5
trichostatin−a_5_0.25
trichostatin−a_2.5_0.125
trichostatin−a_1.25_0.0625
trichostatin−a_0.625_0.03125
trichostatin−a_0.3125_0.015625

Compound: DTT

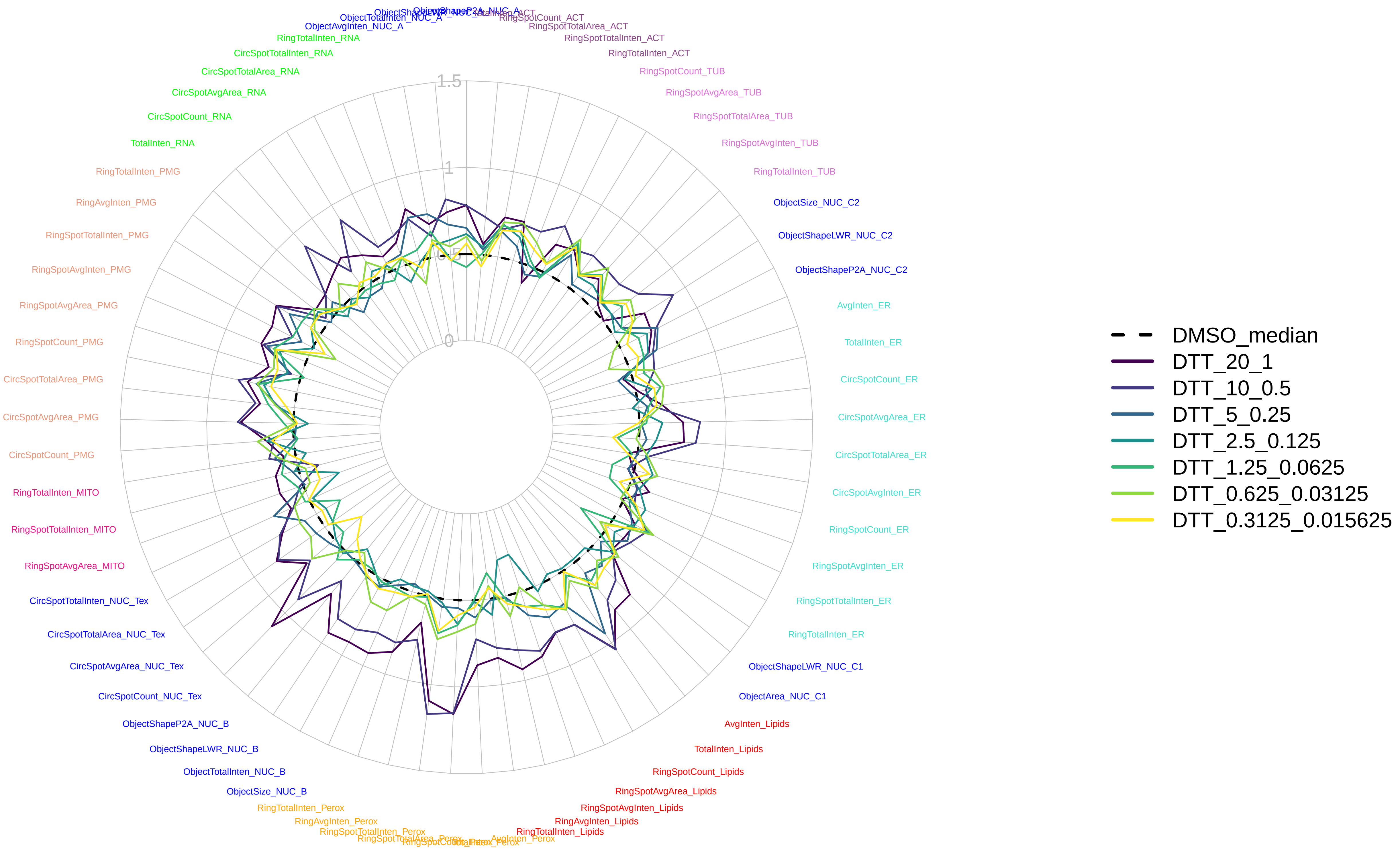

Compound: sirtinol

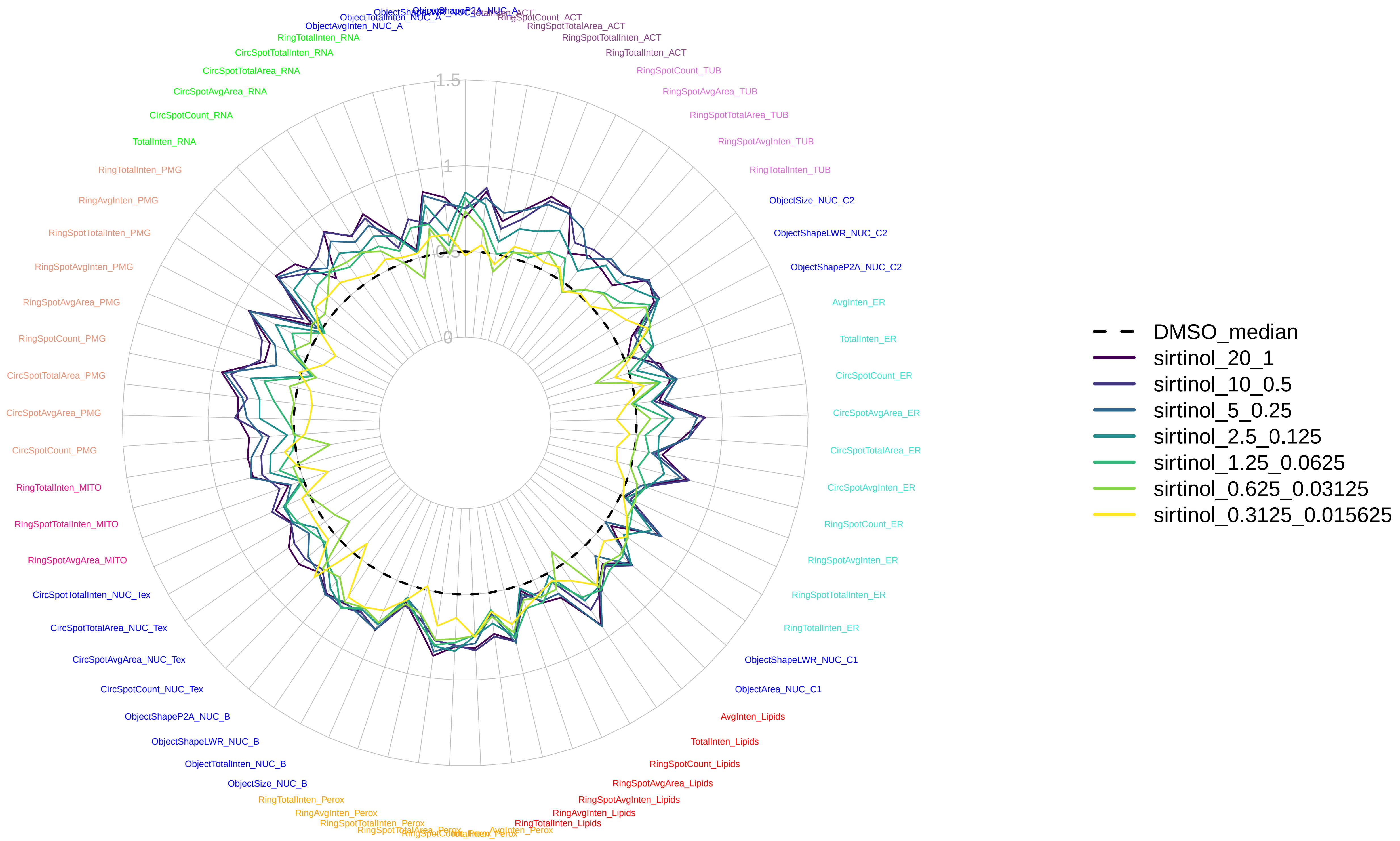

Compound: desipramine

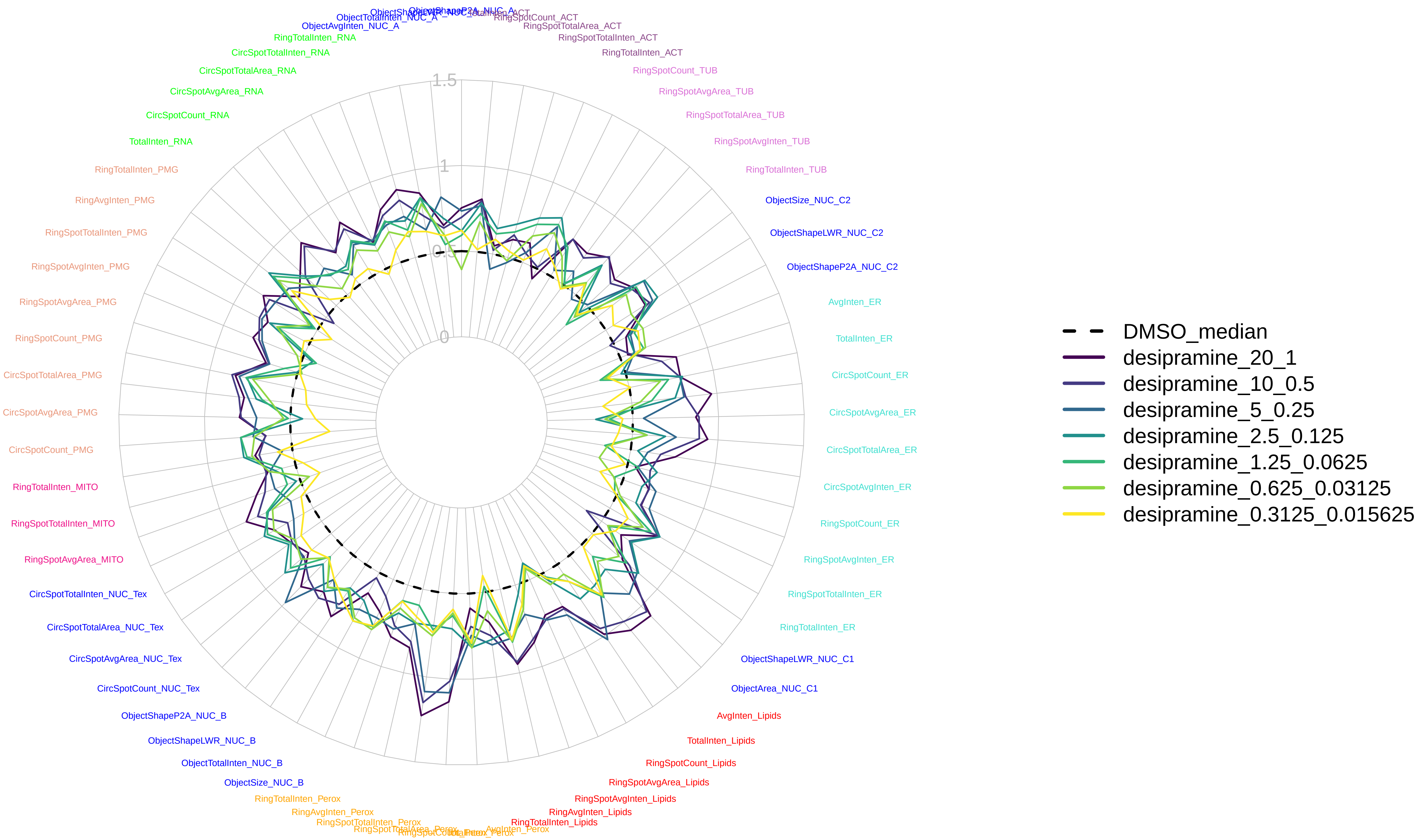

Compound: floxuridine

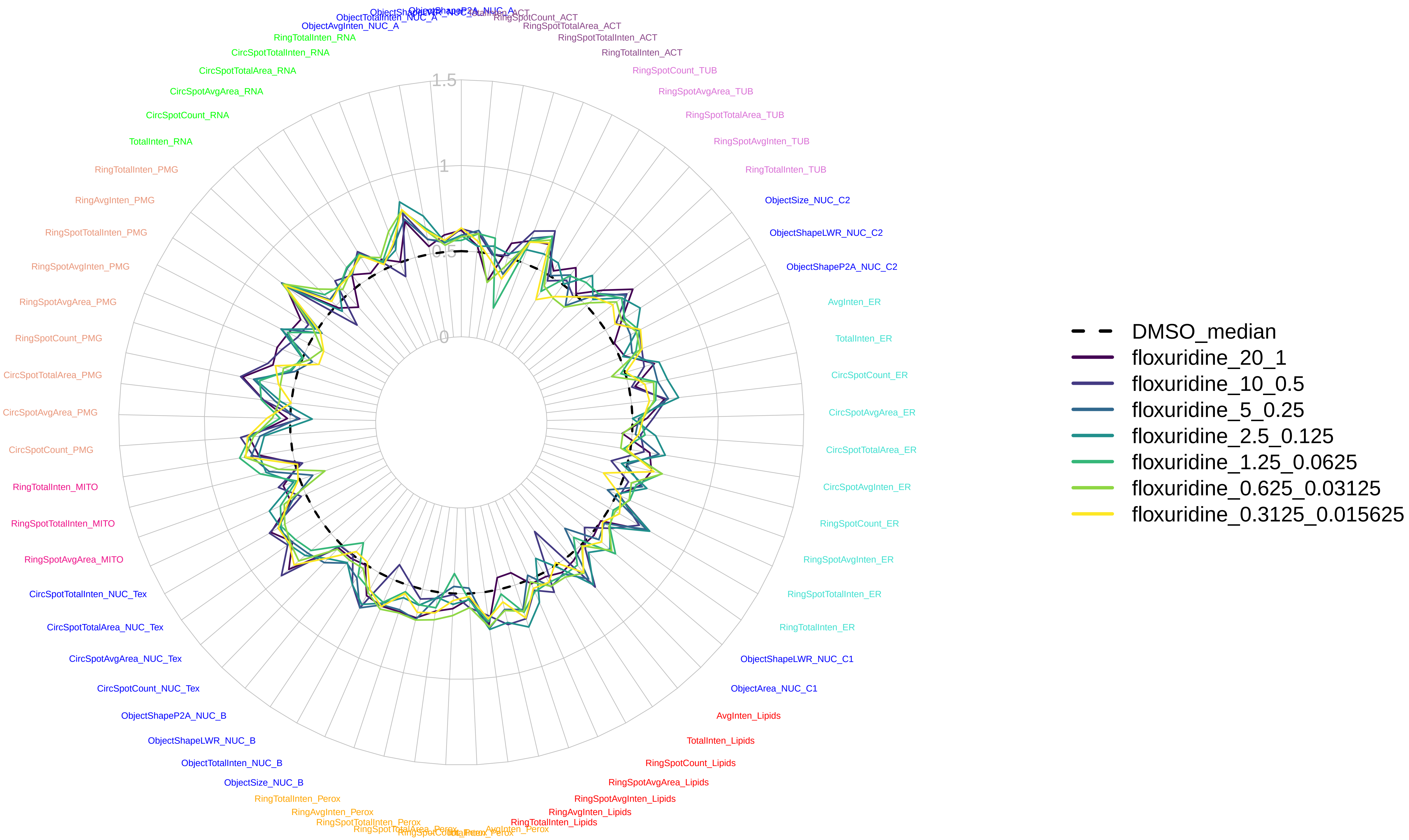

Compound: alvocidib

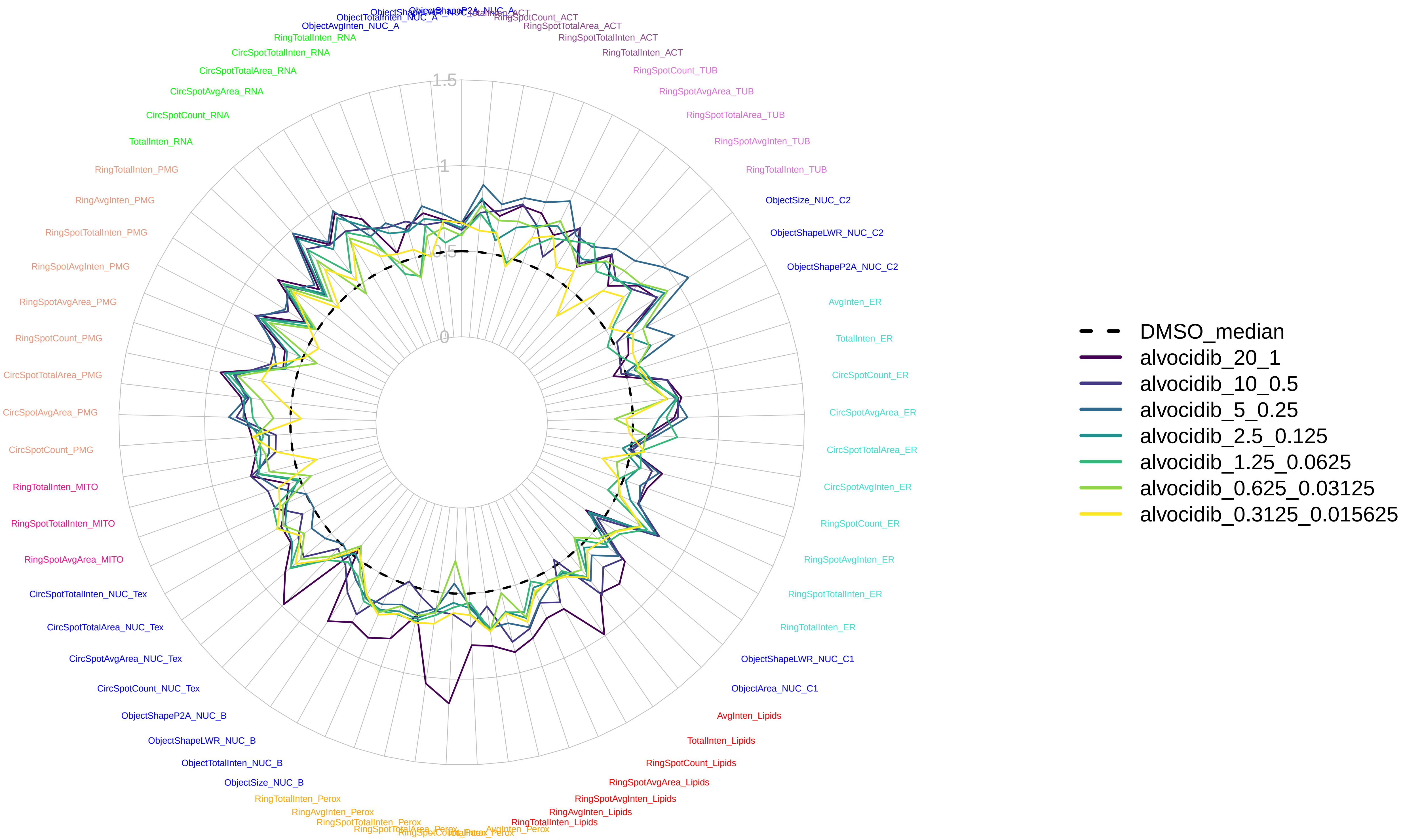

Compound: anisomycin

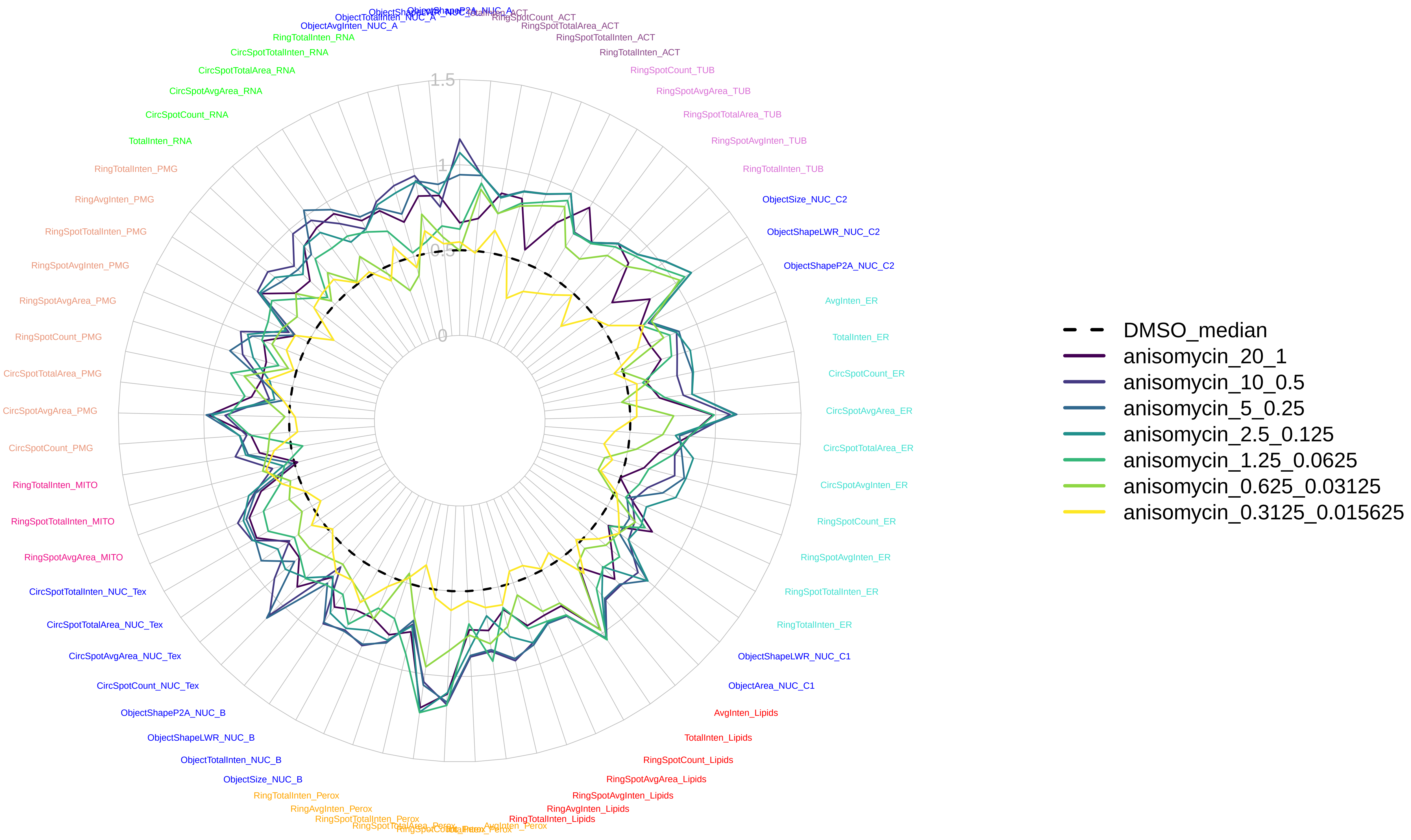

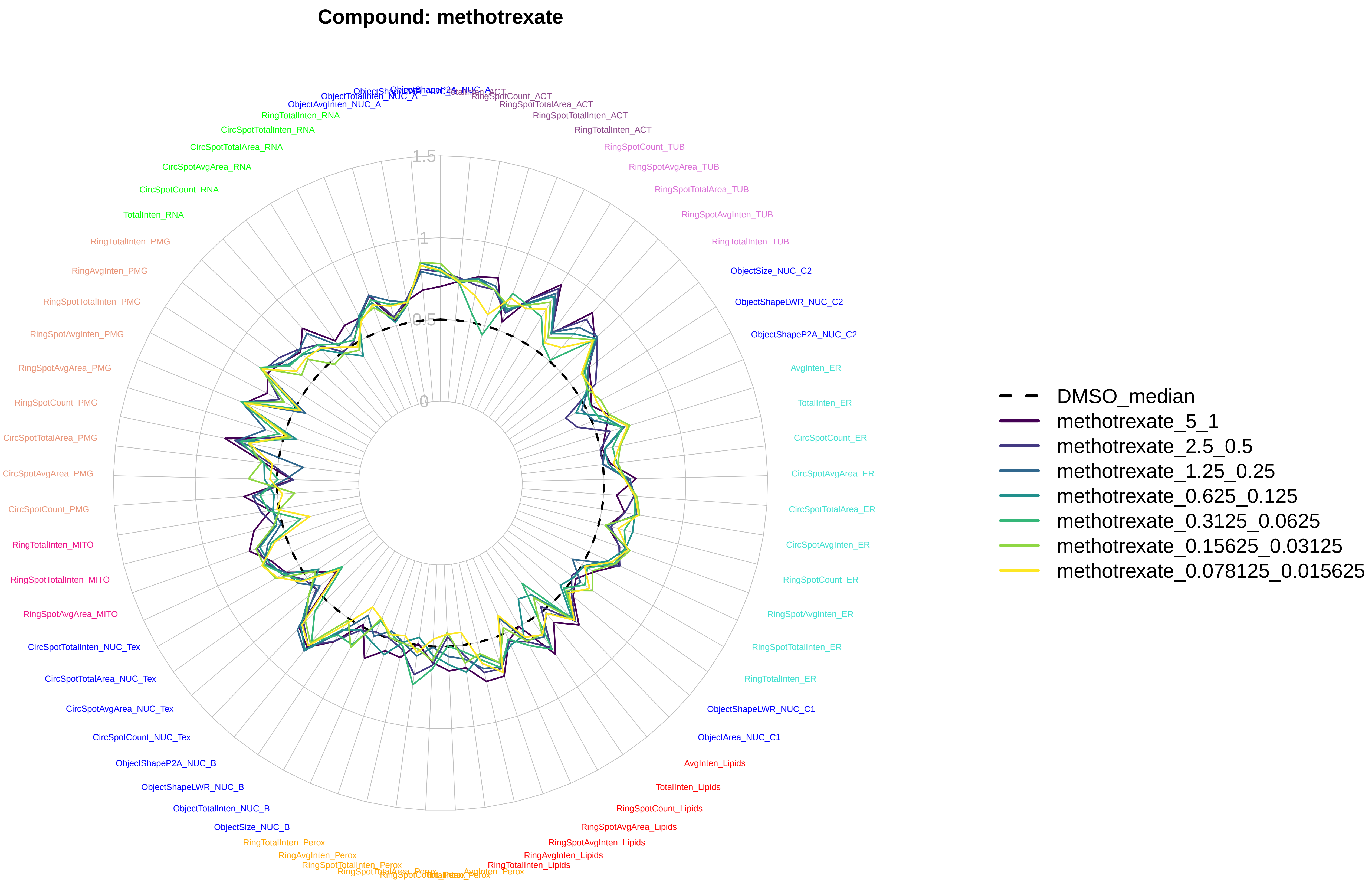
Compound: methotrexate
1.5
1
0.5
0
ObjectShapeP2A_NUC_A
ObjectTotalInten_NUC_A
ObjectAvgInten_NUC_A
RingTotalInten_RNA
CircSpotTotalInten_RNA
CircSpotTotalArea_RNA
CircSpotAvgArea_RNA
CircSpotCount_RNA
TotalInten_RNA
RingTotalInten_PMG
RingAvgInten_PMG
RingSpotTotalInten_PMG
RingSpotAvgInten_PMG
RingSpotAvgArea_PMG
RingSpotCount_PMG
CircSpotTotalArea_PMG
CircSpotAvgArea_PMG
CircSpotCount_PMG
RingTotalInten_MITO
RingSpotTotalInten_MITO
RingSpotAvgArea_MITO
CircSpotTotalInten_NUC_Tex
CircSpotTotalArea_NUC_Tex
CircSpotAvgArea_NUC_Tex
CircSpotCount_NUC_Tex
ObjectShapeP2A_NUC_B
ObjectShapeLWR_NUC_B
ObjectTotalInten_NUC_B
ObjectSize_NUC_B
RingTotalInten_Perox
RingAvgInten_Perox
RingSpotTotalInten_Perox
AvgInten_Perox
RingTotalInten_Lipids
RingAvgInten_Lipids
RingSpotAvgInten_Lipids
RingSpotAvgArea_Lipids
RingSpotCount_Lipids
TotalInten_Lipids
AvgInten_Lipids
ObjectArea_NUC_C1
ObjectShapeLWR_NUC_C1
RingTotalInten_ER
RingSpotTotalInten_ER
RingSpotAvgInten_ER
RingSpotCount_ER
CircSpotAvgInten_ER
CircSpotTotalArea_ER
CircSpotAvgArea_ER
CircSpotCount_ER
TotalInten_ER
AvgInten_ER
ObjectShapeP2A_NUC_C2
ObjectShapeLWR_NUC_C2
ObjectSize_NUC_C2
RingTotalInten_TUB
RingSpotAvgInten_TUB
RingSpotTotalArea_TUB
RingSpotAvgArea_TUB
RingSpotCount_TUB
RingTotalInten_ACT
RingSpotTotalInten_ACT
RingSpotTotalArea_ACT
RingSpotCount_ACT
DMSO_median
methotrexate_5_1
methotrexate_2.5_0.5
methotrexate_1.25_0.25
methotrexate_0.625_0.125
methotrexate_0.3125_0.0625
methotrexate_0.15625_0.03125
methotrexate_0.078125_0.015625

Compound: monensin

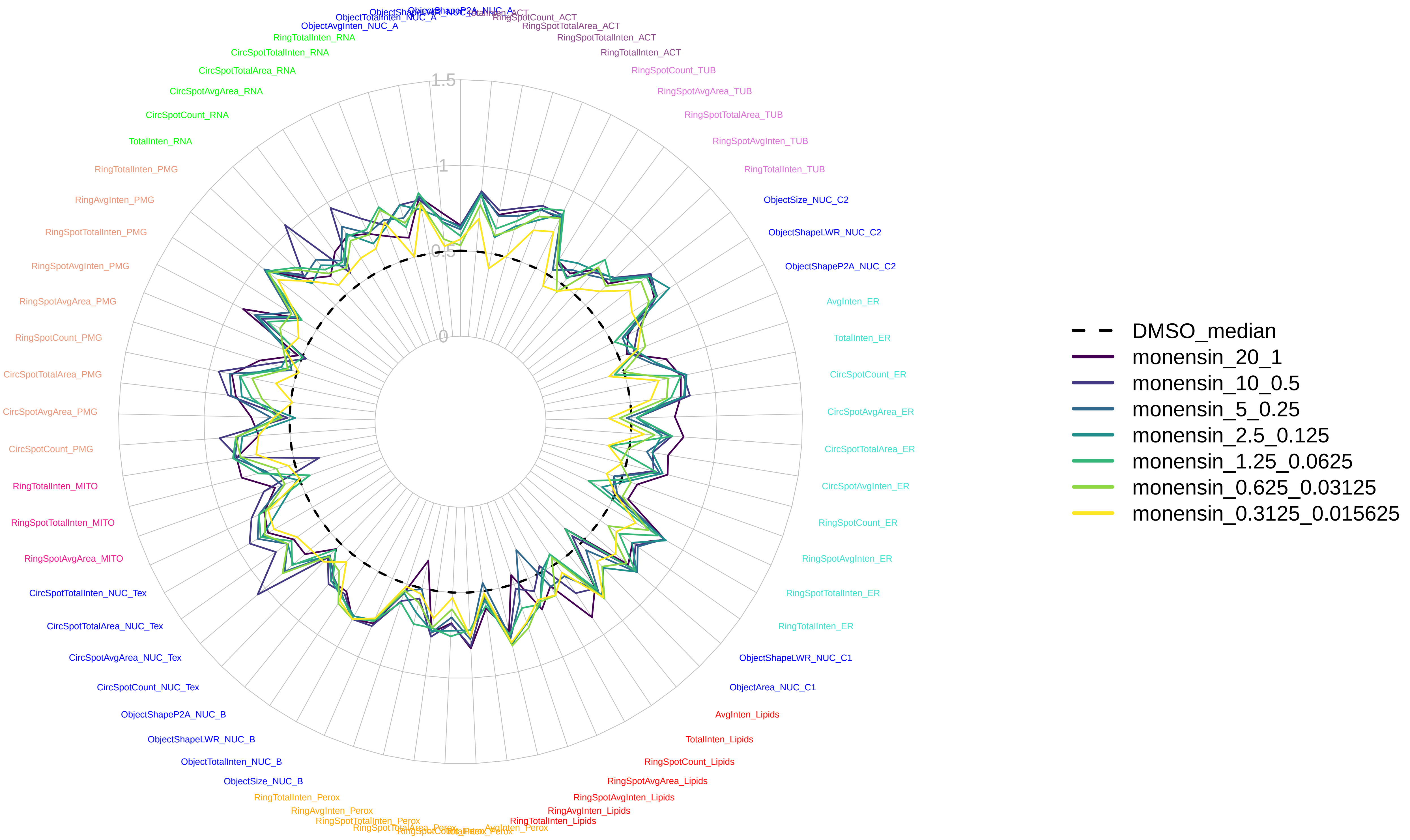

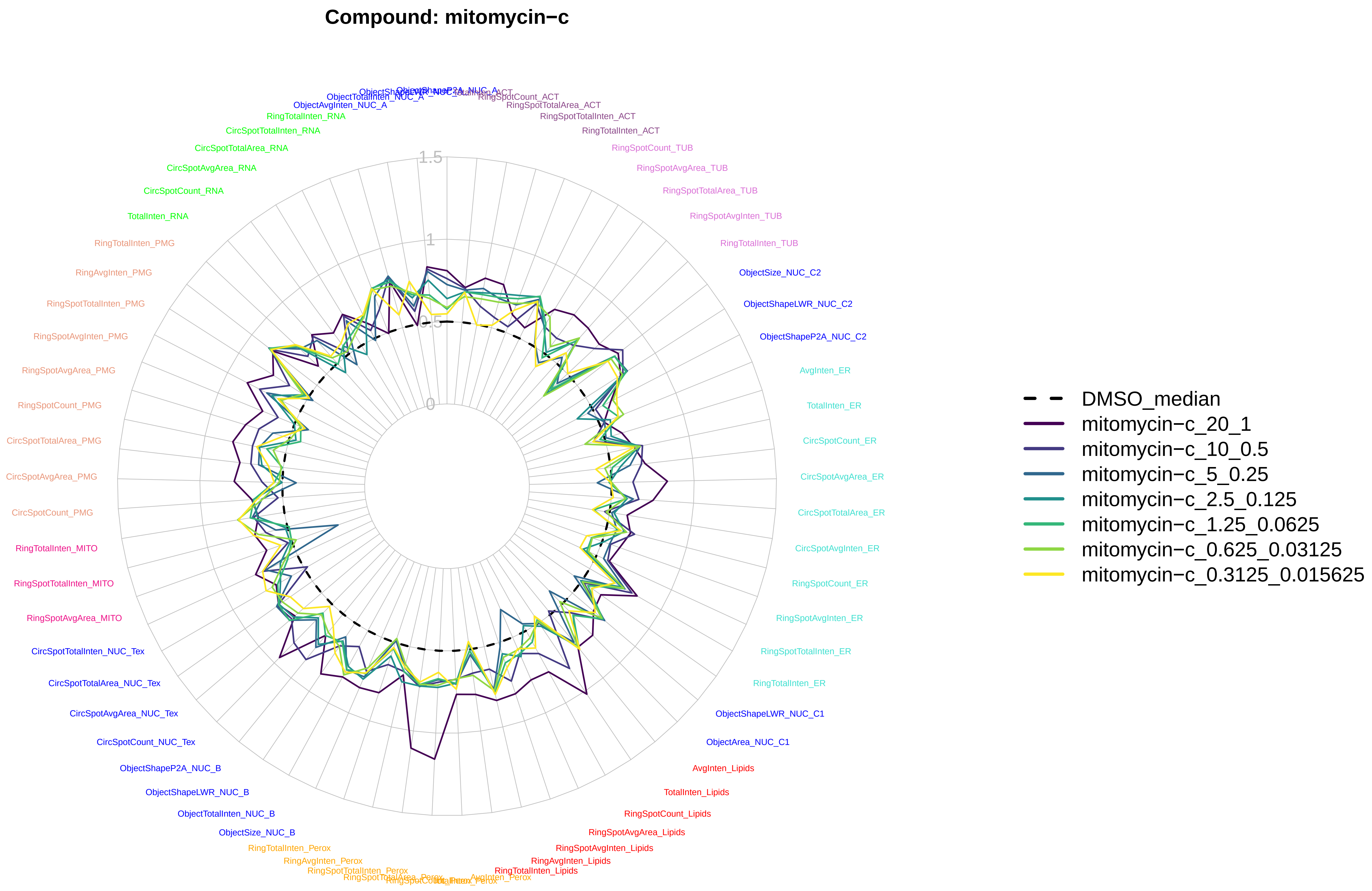

Compound: mitomycin−c
1.5
1
0.5
0
ObjectTotalInten_NUC_A
ObjectAvgInten_NUC_A
RingSpotCount_ACT
RingSpotTotalArea_ACT
RingSpotTotalInten_ACT
RingTotalInten_ACT
RingSpotCount_TUB
RingSpotAvgArea_TUB
RingSpotTotalArea_TUB
RingSpotAvgInten_TUB
RingTotalInten_TUB
ObjectSize_NUC_C2
ObjectShapeLWR_NUC_C2
ObjectShapeP2A_NUC_C2
AvgInten_ER
TotalInten_ER
CircSpotCount_ER
CircSpotAvgArea_ER
CircSpotTotalArea_ER
CircSpotAvgInten_ER
RingSpotCount_ER
RingSpotAvgInten_ER
RingSpotTotalInten_ER
RingTotalInten_ER
ObjectShapeLWR_NUC_C1
ObjectArea_NUC_C1
AvgInten_Lipids
TotalInten_Lipids
RingSpotCount_Lipids
RingSpotAvgArea_Lipids
RingSpotAvgInten_Lipids
RingAvgInten_Lipids
RingTotalInten_Lipids
AvgInten_Perox
RingTotalInten_Perox
RingAvgInten_Perox
RingSpotTotalInten_Perox
ObjectSize_NUC_B
ObjectTotalInten_NUC_B
ObjectShapeLWR_NUC_B
ObjectShapeP2A_NUC_B
CircSpotCount_NUC_Tex
CircSpotAvgArea_NUC_Tex
CircSpotTotalArea_NUC_Tex
CircSpotTotalInten_NUC_Tex
RingSpotAvgArea_MITO
RingSpotTotalInten_MITO
RingTotalInten_MITO
CircSpotCount_PMG
CircSpotAvgArea_PMG
CircSpotTotalArea_PMG
RingSpotCount_PMG
RingSpotAvgArea_PMG
RingSpotAvgInten_PMG
RingSpotTotalInten_PMG
RingAvgInten_PMG
RingTotalInten_PMG
TotalInten_RNA
CircSpotCount_RNA
CircSpotAvgArea_RNA
CircSpotTotalArea_RNA
CircSpotTotalInten_RNA
RingTotalInten_RNA
DMSO_median
mitomycin−c_20_1
mitomycin−c_10_0.5
mitomycin−c_5_0.25
mitomycin−c_2.5_0.125
mitomycin−c_1.25_0.0625
mitomycin−c_0.625_0.03125
mitomycin−c_0.3125_0.015625

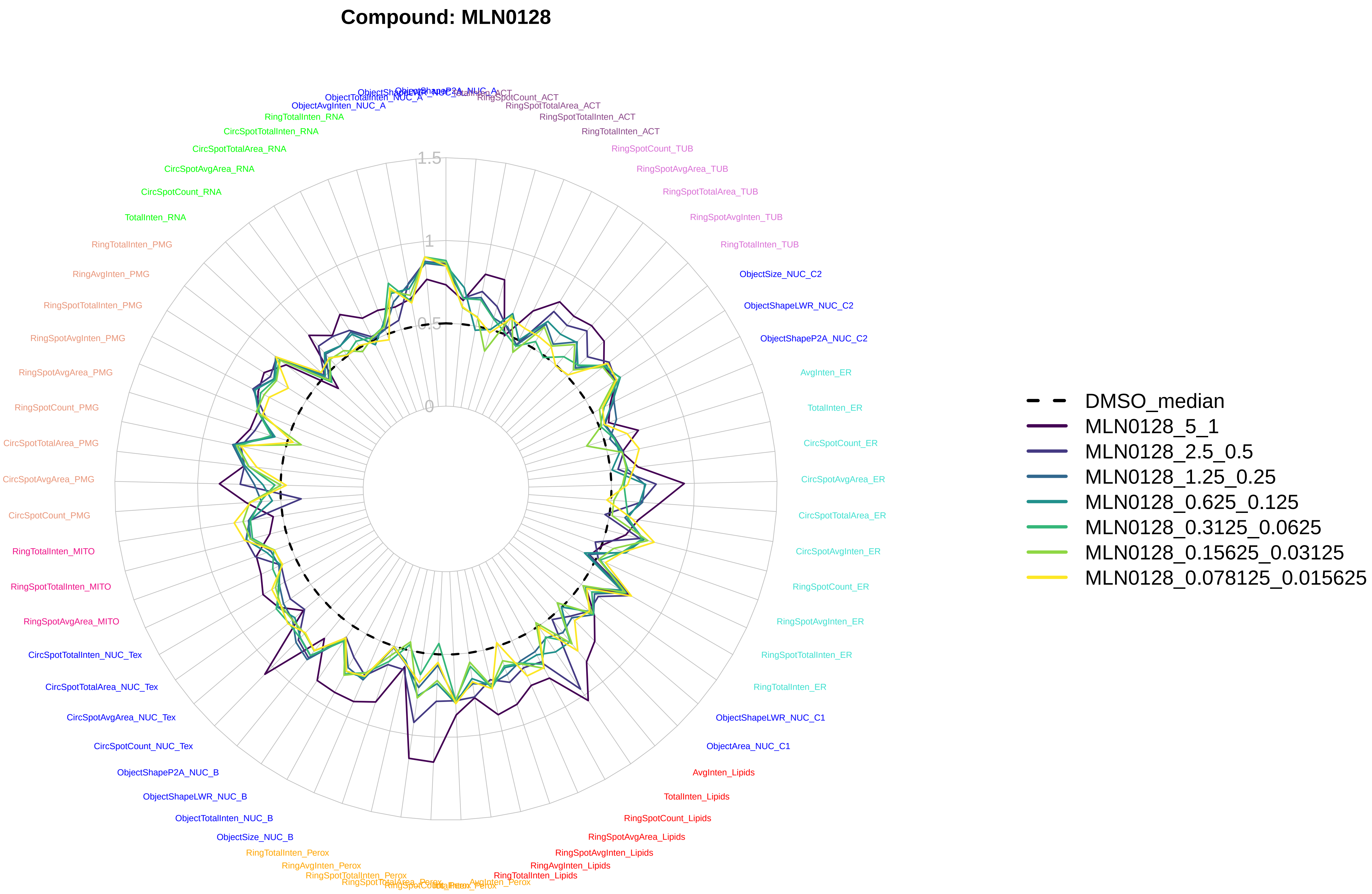
Compound: MLN0128
ObjectTotalInten_NUC_A
ObjectAvgInten_NUC_A
RingTotalInten_RNA
CircSpotTotalInten_RNA
CircSpotTotalArea_RNA
CircSpotAvgArea_RNA
CircSpotCount_RNA
TotalInten_RNA
RingTotalInten_PMG
RingAvgInten_PMG
RingSpotTotalInten_PMG
RingSpotAvgInten_PMG
RingSpotAvgArea_PMG
RingSpotCount_PMG
CircSpotTotalArea_PMG
CircSpotAvgArea_PMG
CircSpotCount_PMG
RingTotalInten_MITO
RingSpotTotalInten_MITO
RingSpotAvgArea_MITO
CircSpotTotalInten_NUC_Tex
CircSpotTotalArea_NUC_Tex
CircSpotAvgArea_NUC_Tex
CircSpotCount_NUC_Tex
ObjectShapeP2A_NUC_B
ObjectShapeLWR_NUC_B
ObjectTotalInten_NUC_B
ObjectSize_NUC_B
RingTotalInten_Perox
RingAvgInten_Perox
RingSpotTotalInten_Perox
RingSpotCount_ACT
RingSpotTotalArea_ACT
RingSpotTotalInten_ACT
RingTotalInten_ACT
RingSpotCount_TUB
RingSpotAvgArea_TUB
RingSpotTotalArea_TUB
RingSpotAvgInten_TUB
RingTotalInten_TUB
ObjectSize_NUC_C2
ObjectShapeLWR_NUC_C2
ObjectShapeP2A_NUC_C2
AvgInten_ER
TotalInten_ER
CircSpotCount_ER
CircSpotAvgArea_ER
CircSpotTotalArea_ER
CircSpotAvgInten_ER
RingSpotCount_ER
RingSpotAvgInten_ER
RingSpotTotalInten_ER
RingTotalInten_ER
ObjectShapeLWR_NUC_C1
ObjectArea_NUC_C1
AvgInten_Lipids
TotalInten_Lipids
RingSpotCount_Lipids
RingSpotAvgArea_Lipids
RingSpotAvgInten_Lipids
RingAvgInten_Lipids
RingTotalInten_Lipids
1.5
1
0.5
0
DMSO_median
MLN0128_5_1
MLN0128_2.5_0.5
MLN0128_1.25_0.25
MLN0128_0.625_0.125
MLN0128_0.3125_0.0625
MLN0128_0.15625_0.03125
MLN0128_0.078125_0.015625

Compound: brefeldin−a

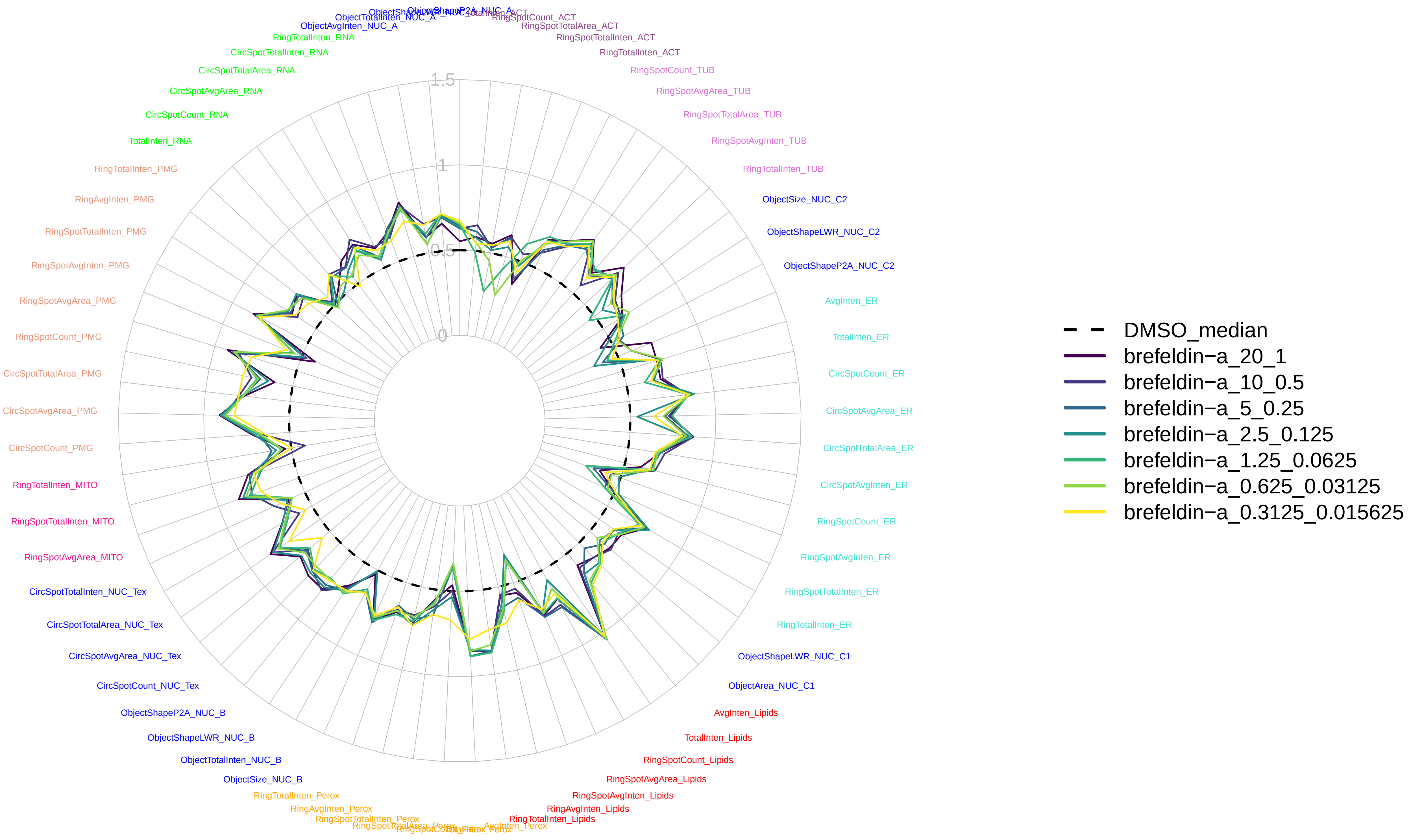

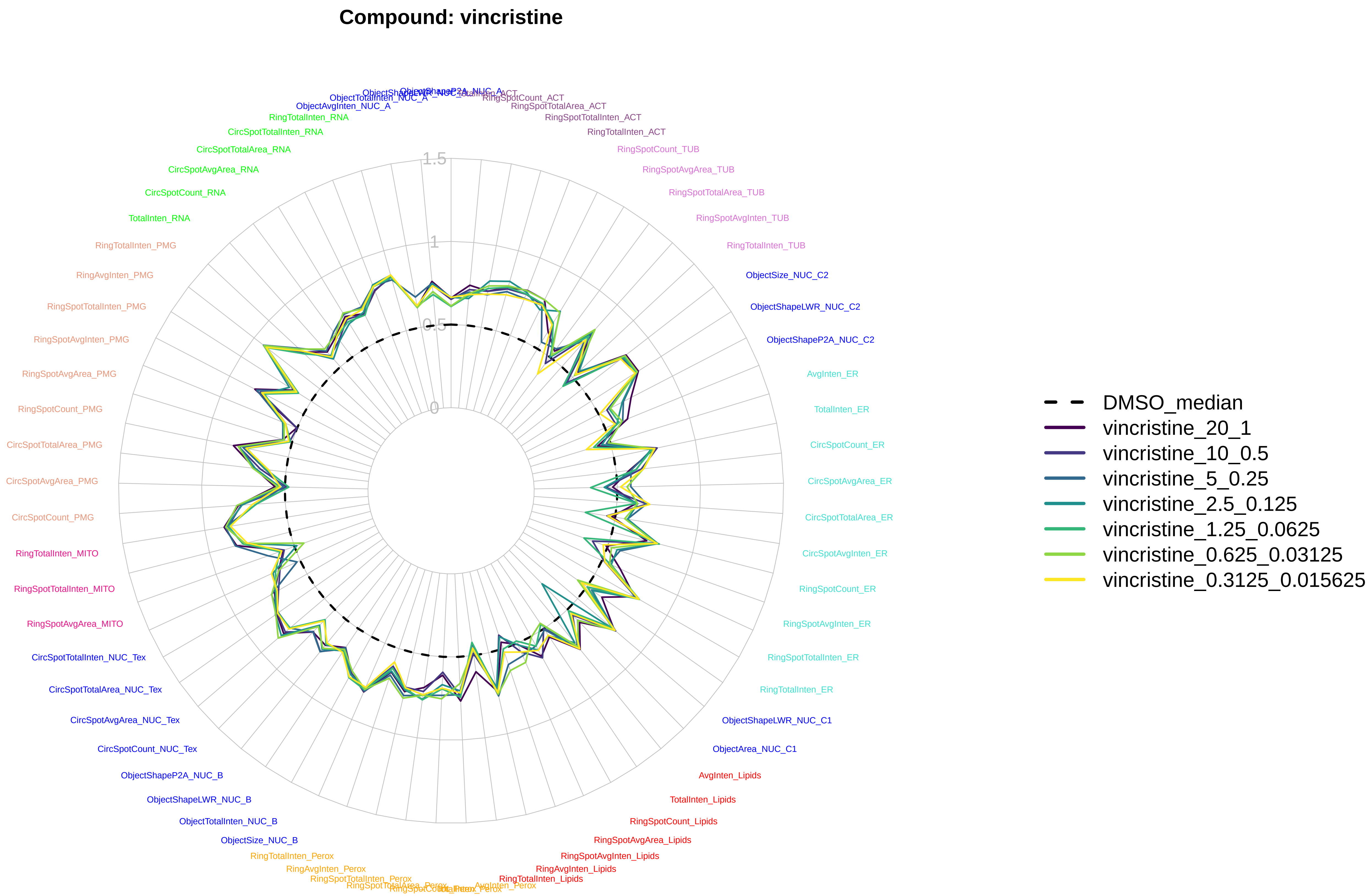
Compound: vincristine
ObjectShapeP2A_NUC_A
ObjectTotalInten_NUC_A
ObjectAvgInten_NUC_A
RingSpotCount_ACT
RingSpotTotalArea_ACT
RingSpotTotalInten_ACT
RingTotalInten_ACT
RingSpotCount_TUB
RingSpotAvgArea_TUB
RingSpotTotalArea_TUB
RingSpotAvgInten_TUB
RingTotalInten_TUB
ObjectSize_NUC_C2
ObjectShapeLWR_NUC_C2
ObjectShapeP2A_NUC_C2
AvgInten_ER
TotalInten_ER
CircSpotCount_ER
CircSpotAvgArea_ER
CircSpotTotalArea_ER
CircSpotAvgInten_ER
RingSpotCount_ER
RingSpotAvgInten_ER
RingSpotTotalInten_ER
RingTotalInten_ER
ObjectShapeLWR_NUC_C1
ObjectArea_NUC_C1
AvgInten_Lipids
TotalInten_Lipids
RingSpotCount_Lipids
RingSpotAvgArea_Lipids
RingSpotAvgInten_Lipids
RingAvgInten_Lipids
RingTotalInten_Lipids
AvgInten_Perox
RingSpotTotalInten_Perox
RingAvgInten_Perox
RingTotalInten_Perox
ObjectSize_NUC_B
ObjectTotalInten_NUC_B
ObjectShapeLWR_NUC_B
ObjectShapeP2A_NUC_B
CircSpotCount_NUC_Tex
CircSpotAvgArea_NUC_Tex
CircSpotTotalArea_NUC_Tex
CircSpotTotalInten_NUC_Tex
RingSpotAvgArea_MITO
RingSpotTotalInten_MITO
RingTotalInten_MITO
CircSpotCount_PMG
CircSpotAvgArea_PMG
CircSpotTotalArea_PMG
RingSpotCount_PMG
RingSpotAvgArea_PMG
RingSpotAvgInten_PMG
RingSpotTotalInten_PMG
RingAvgInten_PMG
RingTotalInten_PMG
TotalInten_RNA
CircSpotCount_RNA
CircSpotAvgArea_RNA
CircSpotTotalArea_RNA
CircSpotTotalInten_RNA
RingTotalInten_RNA
1.5
1
0.5
0
DMSO_median
vincristine_20_1
vincristine_10_0.5
vincristine_5_0.25
vincristine_2.5_0.125
vincristine_1.25_0.0625
vincristine_0.625_0.03125
vincristine_0.3125_0.015625

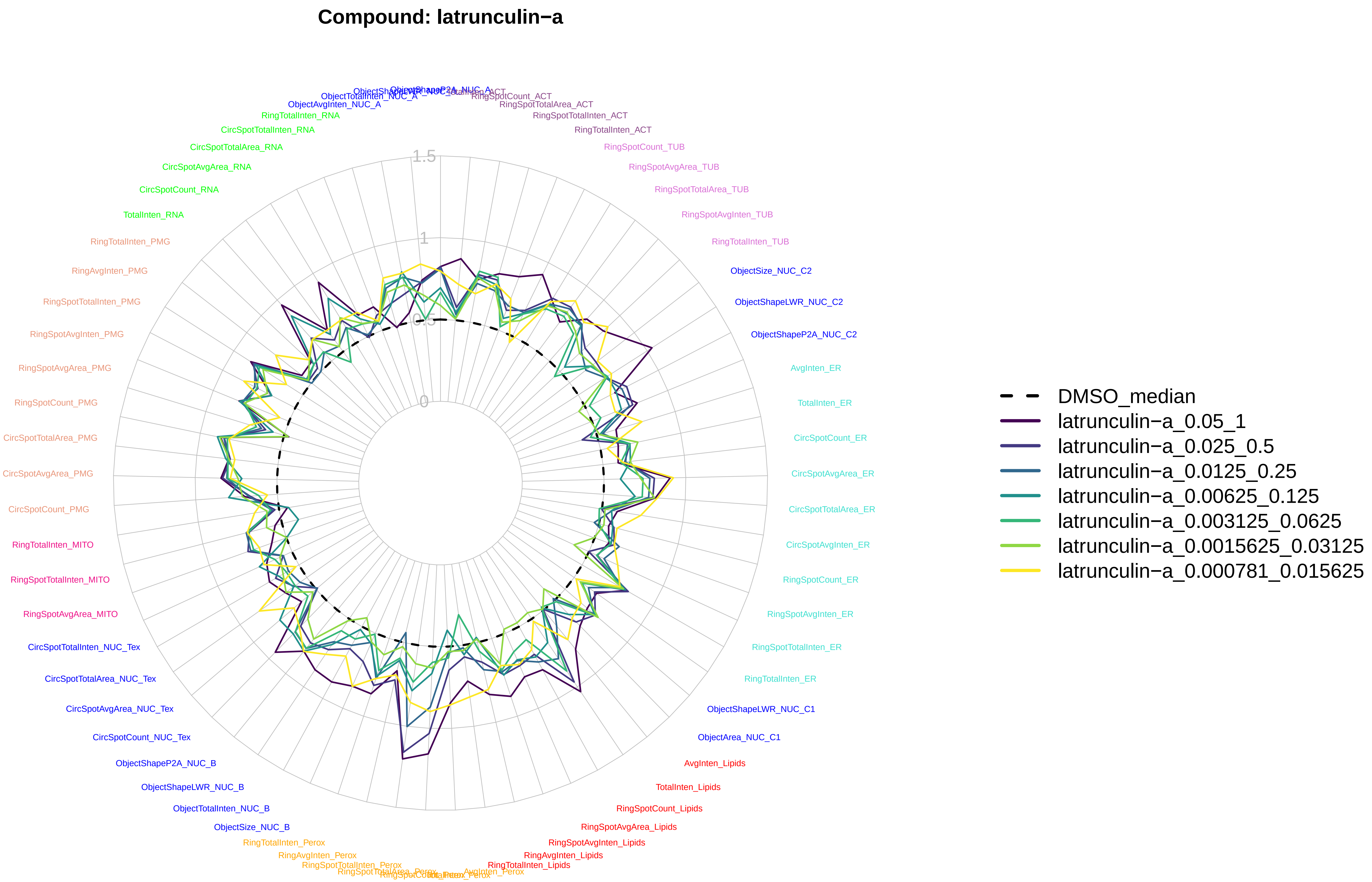

Compound: latrunculin−a
ObjectAvgInten_NUC_A
RingSpotCount_ACT
RingSpotTotalArea_ACT
RingSpotTotalInten_ACT
RingTotalInten_ACT
RingSpotCount_TUB
RingSpotAvgArea_TUB
RingSpotTotalArea_TUB
RingSpotAvgInten_TUB
RingTotalInten_TUB
ObjectSize_NUC_C2
ObjectShapeLWR_NUC_C2
ObjectShapeP2A_NUC_C2
AvgInten_ER
TotalInten_ER
CircSpotCount_ER
CircSpotAvgArea_ER
CircSpotTotalArea_ER
CircSpotAvgInten_ER
RingSpotCount_ER
RingSpotAvgInten_ER
RingSpotTotalInten_ER
RingTotalInten_ER
ObjectShapeLWR_NUC_C1
ObjectArea_NUC_C1
AvgInten_Lipids
TotalInten_Lipids
RingSpotCount_Lipids
RingSpotAvgArea_Lipids
RingSpotAvgInten_Lipids
RingAvgInten_Lipids
RingTotalInten_Lipids
RingTotalInten_Perox
RingAvgInten_Perox
RingSpotTotalInten_Perox
ObjectSize_NUC_B
ObjectTotalInten_NUC_B
ObjectShapeLWR_NUC_B
ObjectShapeP2A_NUC_B
CircSpotCount_NUC_Tex
CircSpotAvgArea_NUC_Tex
CircSpotTotalArea_NUC_Tex
CircSpotTotalInten_NUC_Tex
RingSpotAvgArea_MITO
RingSpotTotalInten_MITO
RingTotalInten_MITO
CircSpotCount_PMG
CircSpotAvgArea_PMG
CircSpotTotalArea_PMG
RingSpotCount_PMG
RingSpotAvgArea_PMG
RingSpotAvgInten_PMG
RingSpotTotalInten_PMG
RingAvgInten_PMG
RingTotalInten_PMG
TotalInten_RNA
CircSpotCount_RNA
CircSpotAvgArea_RNA
CircSpotTotalArea_RNA
CircSpotTotalInten_RNA
RingTotalInten_RNA
1.5
1
0
DMSO_median
latrunculin−a_0.05_1
latrunculin−a_0.025_0.5
latrunculin−a_0.0125_0.25
latrunculin−a_0.00625_0.125
latrunculin−a_0.003125_0.0625
latrunculin−a_0.0015625_0.03125
latrunculin−a_0.000781_0.015625
