# Supplementary material for: A statistical framework for high-content phenotypic profiling using cellular feature distributions
Source: Commun Biol. 2022 Dec 22;5:1409. doi: 10.1038/s42003-022-04343-3 (PMC9780213; doi:10.1038/s42003-022-04343-3)
Supplement: Supplementary file 3 — Description of Supplementary Data [file 42003_2022_4343_MOESM3_ESM.pdf]

Supplementary data files description: Supplementary Data 1 - 6

A statistical framework for high-content phenotypic profiling using cellular feature distributions

Yanthe E. Pearson[1], Stephan Kremb[1], Glenn L. Butterfoss[1], Xin Xie[1], Hala Fahs[1], Kristin C. Gunsalus[1,2*]

[1]Center for Genomics and Systems Biology, New York University Abu Dhabi, P. O. Box 129188, Abu Dhabi, United Arab Emirates

[2]Department of Biology and Center for Genomics and Systems Biology, New York University, New York, NY 10003, USA

* Corresponding author: Kristin C. Gunsalus
Email: kcg1@nyu.edu

File name: Supplementary Data 1
Description: Summary of two-way ANOVA analysis including F-statistic and corresponding p-value for each plate and feature (see Fig. 3b).

File name: Supplementary Data 2
Description: Output summary and metadata of replicate reproducibility analysis of treatment replicates and control-DMSO replicates includes well id, sample size, KS distance (corresponding p-value), EMD distance, Z-score (see Fig. 4c).

File name: Supplementary Data 3
Description: List of raw cell counts (n) corresponding to each measured EMD score calculated for 330 control cell samples, and 455 treatment samples relative to the global control population (see Fig. 5b, c).

File name: Supplementary Data 4
Description: Per-compound metadata from multiple sources,
including compound dilutions, plate location, biological targets, MOA and chemical descriptors.

File name: Supplementary Data 5
Description: Summary of feature analysis includes full feature set, irreproducible features, circ features, redundant (by correlation) features, low relative variance features, and the final set of active features.

File name: Supplementary Data 6
Description: Three sets of treatment profiles and accompanying metadata; Raw EMD profiles include the full 174 feature set, profiles of 52 treatments causing strong cell loss (> 70%) and scaled EMD profiles (log transformed and min-max scaled) with the reduced 69 feature set.
